# DeLTa-Seq: direct-lysate targeted RNA-Seq from crude tissue lysate

**DOI:** 10.1186/s13007-022-00930-x

**Published:** 2022-08-06

**Authors:** Makoto Kashima, Mari Kamitani, Yasuyuki Nomura, Natsumi Mori-Moriyama, Shigeyuki Betsuyaku, Hiromi Hirata, Atsushi J. Nagano

**Affiliations:** 1 Research Institute for Food and Agriculture, Ryukoku University, Yokotani 1-5, Seta Oe-cho, Otsu, Shiga 520-2194 Japan; 2 Department of Chemistry and Biological Science, College of Science and Engineering, Aoyama Gakuin University, Fuchinobe 5-10-1, Chuoku, , Sagamihara 252-5258 Japan; 3 Center for Ecological Research, Kyoto University, Hirano 2-509-3, Otsu, Shiga 520-2113 Japan; 4 Faculty of Agriculture, Ryukoku University, Yokotani 1-5, Seta Oe-cho, Otsu, Shiga 520-2194 Japan; 5 Institute for Advanced Biosciences, Keio University, 403-1 Nipponkoku, Daihouji, Tsuruoka, Yamagata 997-0017 Japan

**Keywords:** Direct-lysate reverse transcription, Targeted RNA-Seq, Reducing reagent, Gene expression analysis

## Abstract

**Background:**

Quantification of gene expression such as RNA-Seq is a popular approach to study various biological phenomena. Despite the development of RNA-Seq library preparation methods and sequencing platforms in the last decade, RNA extraction remains the most laborious and costly step in RNA-Seq of tissue samples of various organisms. Thus, it is still difficult to examine gene expression in thousands of samples.

**Results:**

Here, we developed Direct-RT buffer in which homogenization of tissue samples and direct-lysate reverse transcription can be conducted without RNA purification. The DTT concentration in Direct-RT buffer prevented RNA degradation but not RT in the lysates of several plant tissues, yeast, and zebrafish larvae. Direct reverse transcription on these lysates in Direct-RT buffer produced comparable amounts of cDNA to those synthesized from purified RNA. To maximize the advantage of the Direct-RT buffer, we integrated Direct-RT and targeted RNA-Seq to develop a cost-effective, high-throughput quantification method for the expressions of hundreds of genes: DeLTa-Seq (Direct-Lysate reverse transcription and Targeted RNA-Seq). The DeLTa-Seq method could drastically improve the efficiency and accuracy of gene expression analysis. DeLTa-Seq analysis of 1056 samples revealed the temperature-dependent effects of jasmonic acid and salicylic acid in *Arabidopsis thaliana*.

**Conclusions:**

The DeLTa-Seq method can realize large-scale studies using thousands of animal, plant, and microorganism samples, such as chemical screening, field experiments, and studies focusing on individual variability. In addition, Direct-RT is also beneficial for gene expression analysis in small tissues from which it is difficult to purify enough RNA for the experiments.

**Supplementary Information:**

The online version contains supplementary material available at 10.1186/s13007-022-00930-x.

## Background

Quantification of gene expression is a popular approach to study various biological phenomena [1–8]. Recently, large-scale transcriptome analysis by RNA-Seq of hundreds of samples has emerged in plant biology [9–12]. Although the cost of sequencing has reduced drastically in the last decade [13], the labor requirements of the library preparation step obstruct the routine use of RNA-Seq. To overcome the difficulties, several high-throughput RNA-Seq library preparation methods have been developed [14–19]. Particularly, two methods developed based on the 3′ RNA-Seq protocols for single-cell RNA-Seq, namely Lasy-Seq [19] and BRB-Seq [18], enable the early pooling of samples, thereby reducing labor and achieving a cost of approximately two dollars per sample. In this situation, RNA extraction remains the most laborious and costly step in RNA-Seq. It is more expensive to purify RNA from plant samples than from cultured animal cells, particularly because plants tend to contain large amounts of polysaccharides and have high RNase activity [20]. To obtain high-quality RNA from plant samples, samples are usually homogenized and lysed in a buffer containing guanidine hydrochloride to denature RNase, followed by RNA purification with phenol chloroform extraction, a silica column, or magnetic beads that selectively bind to nucleic acid. If these RNA extraction steps can be skipped, the required cost and labor for RNA-Seq will be substantially reduced, which will facilitate the use of RNA-Seq in large-scale studies with thousands of samples. Although several studies have shown that RT can be directly obtained from the lysate of cultured animal cells in cell-lysis buffers [21–23], to our knowledge, no study has achieved direct lysate RT from crude tissue lysates. A previous study showed that reducing reagents such as dithiothreitol (DTT) irreversibly deactivate intrinsic RNase in wheat [24]. However, whether DTT can protect RNA in the lysate of other species without inhibition of reverse transcription has not been examined.

There is a trade-off between comprehensive gene quantification and the required amount of sequence data [25]. For example, in the case of the human transcriptome, it is estimated that ∼ 40 million reads are required for the reliable quantification of moderately abundant transcripts, and as many as 500 million reads are required to comprehensively quantify rare transcripts such as transcription factors [26, 27]. In some cases, it is sufficient to focus on the limited number of genes of interest. When we focus on the limited number of genes, we can measure gene expression more accurately with a small number of reads. This will enable us to examine biological phenomena with higher resolution of time, space, perturbation, and individual variability. Thus, cost-effective methods of gene quantification such as targeted RNA-Seq and related techniques that can quantify hundreds of transcripts [28–31] are required to understand the complex behaviors of biological systems.

Jasmonic acid (JA) and salicylic acid (SA) are majorly involved in defense and stress responses in plants [32]. Although defense responses of plants could vary under high temperature due to global warming [33, 34], little is known about the changes that occur in gene expression levels related to JA and SA with temperature [35]. This could be partially because a large number of samples need to be analyzed, which is a laborious and costly process.

Here, we showed that a high concentration of DTT can inhibit intrinsic RNase in plant and animal crude tissue lysates, but does not inhibit reverse transcriptase. Using Direct-RT buffer containing DTT, we succeeded in direct-lysate RT from plant, yeast, and animal samples and RNA-Seq using the lysate. Finally, we combined direct-lysate RT and targeted RNA-Seq into a new method called DeLTa-Seq, which can drastically reduce the cost and labor of RNA-Seq, whilst maintaining accuracy in quantification. DeLTa-Seq will pave the way for large-scale research.

## Results

### Reducing regents inhibit RNA degradation in plant, yeast, and animal lysates

For direct-lysate RT from plant tissues, we trialed three reagents [CL buffer [23], CellAmp (TaKaRa, Kusatsu, Japan), and SuperPrep (TOYOBO, Osaka, Japan)] designed for direct-lysate RT from cultured mammalian cells. *Arabidopsis thaliana* seedlings were homogenized in these reagents, followed by a 1 h incubation at 22 °C. RNA was purified from the lysates and checked by electrophoresis. Frozen rice (*Oryza sativa*) leaves were also homogenized with zirconia beads and dissolved in the reagents. Incubation and RNA purification were conducted as described above. In both cases, no peaks of 18S or 28S rRNA were detected, but short fragments of degraded RNAs were observed (Additional file 4: Fig. S1). The electropherograms indicated that these reagents for direct-lysate RT from cultured mammalian cells cannot inhibit RNA degradation in plant lysates.

Bioanalyzer electropherograms of purified RNA from plants and zebrafish larva lysates homogenized in CL buffer and buffers from the CellAmp and SuperPrep kits.

**Fig. 1:**
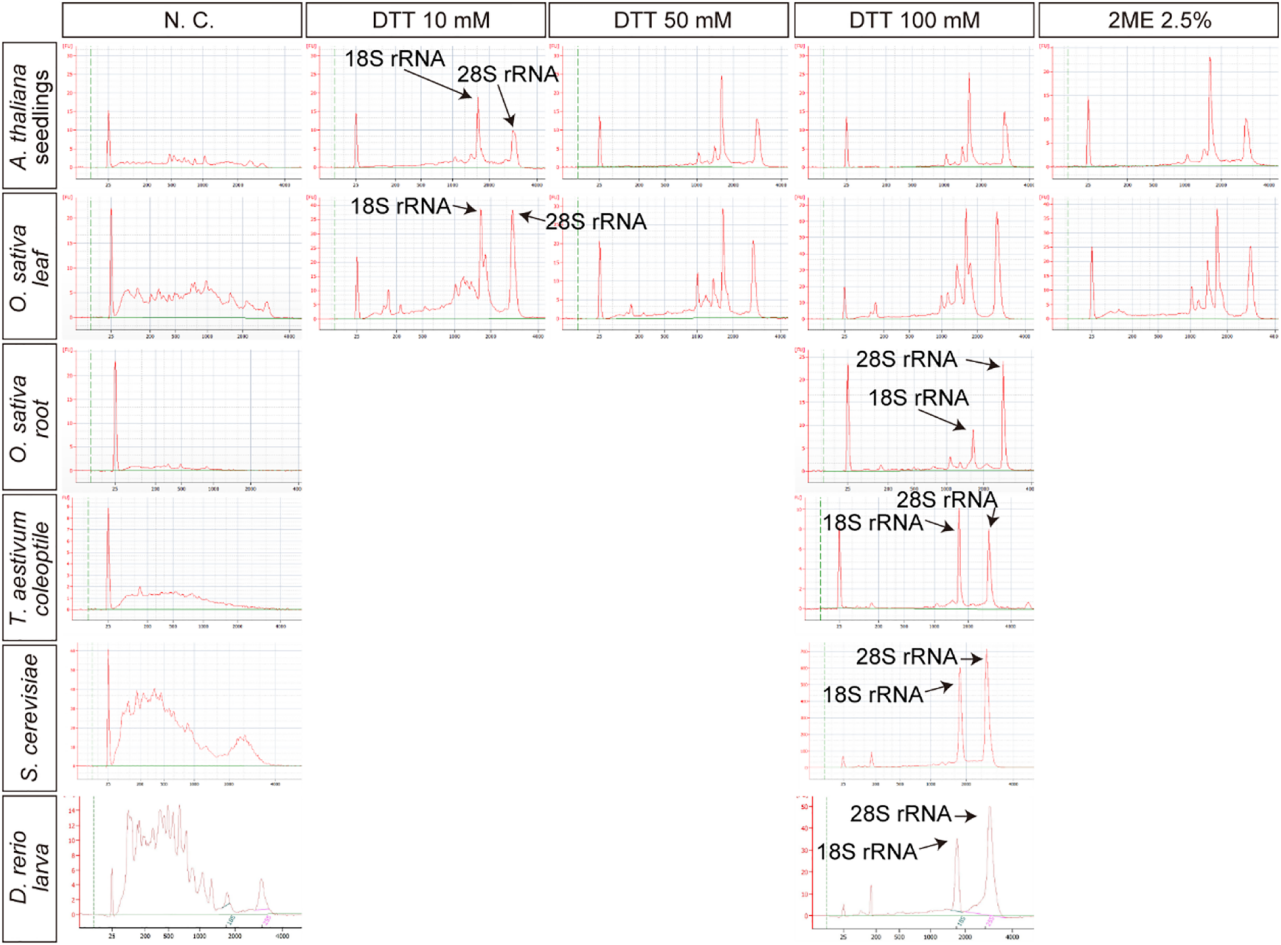
Reducing reagents inhibited RNA degradation in plant and animal tissue lysate. Bioanalyzer electropherograms of purified RNA from tissue lysates (*A. thaliana* seedlings, *O. sativa* leaves, *O. sativa* root, *T. aestivum* coleoptile, *S. cerevisiae*, and *D. rerio*) containing the reducing reagents dithiothreitol (DTT) and 2-mercaptoethanol (2ME). The columns indicate the concentrations of the reducing reagents in the homogenization buffers. N.C. represents no reducing reagents in the buffers (negative control)

In a previous study [24], inactivation of RNase in an extract prepared from dark-grown wheat required 0.5 mM DTT or more than 0.014% 2-mercaptoethanol (2ME) [24]. Thus, we examined the ability of reducing reagents to protect RNA in plant lysates from RNase. We prepared 90 mM Tris–HCl (pH 7.6) containing 10 mM, 50 mM, or 100 mM DTT or 2.5% 2ME, and homogenized *A. thaliana* seedlings in these buffers. Following a 1 h incubation at 22 °C, RNA was purified from the lysates, followed by electrophoresis to check the quality. No peaks were observed in the electropherogram of purified RNA from the lysate in the negative control buffer (no reducing reagents; Fig. 1), indicating that RNA was degraded in the lysate. In contrast, clear peaks of rRNAs were observed in the electropherograms of purified RNA from lysates containing DTT or 2ME. In the 10 mM DTT buffer, slight degradation of rRNA was observed in the region shorter than the 18S rRNA (Fig. 1). Degradation of RNA was inhibited in the buffers with higher concentrations of DTT or 2.5% 2ME (RIN 7.7–8.5, rRNA ratio[25S/18S] > 0.7) (Fig. 1). In addition, we examined the effect of adding reducing regents to the lysates of *O. sativa* leaf and root and *Triticum aestivum* coleoptile with the first leaf. Frozen rice leaves and wheat coleoptiles were homogenized with zirconia beads, and Tris–HCl buffers containing the reducing regents were added. As for *A. thaliana,* after a 1 h incubation at 22 °C, degradation of rRNA was observed in the lysates in the buffers without any reducing reagents and with 10 mM DTT, but not 50 mM or 100 mM DTT or 2.5% 2ME (RIN 7.1–9.0, rRNA ratio[25S/18S] > 0.9) (Fig. 1). Furthermore, we examined whether 100 mM DTT can protect RNA from degradation in the lysates of yeast and animal tissue (zebrafish larvae). A pellet of liquid-cultured *Saccharomyces cerevisiae* was homogenized with zirconia beads, and 2 days post-fertilization zebrafish larvae were homogenized with a pestle in Tris–HCl buffer with or without 100 mM DTT, followed by a 1 h incubation at 22 °C and purification of total RNA. As expected, clear rRNA peaks were observed in the RNA from lysates with DTT but not from those without DTT (Fig. 1). It is noted that the commercial reagents for cultured animal cells could not prevent RNA degradation in the lysate of zebrafish (Additional file 4: Fig. S1). We therefore concluded that a high concentration of reducing reagents can protect RNA from intrinsic RNase.

### Moderate concentrations of reducing reagents do not inhibit reverse transcription

**Fig. 2:**
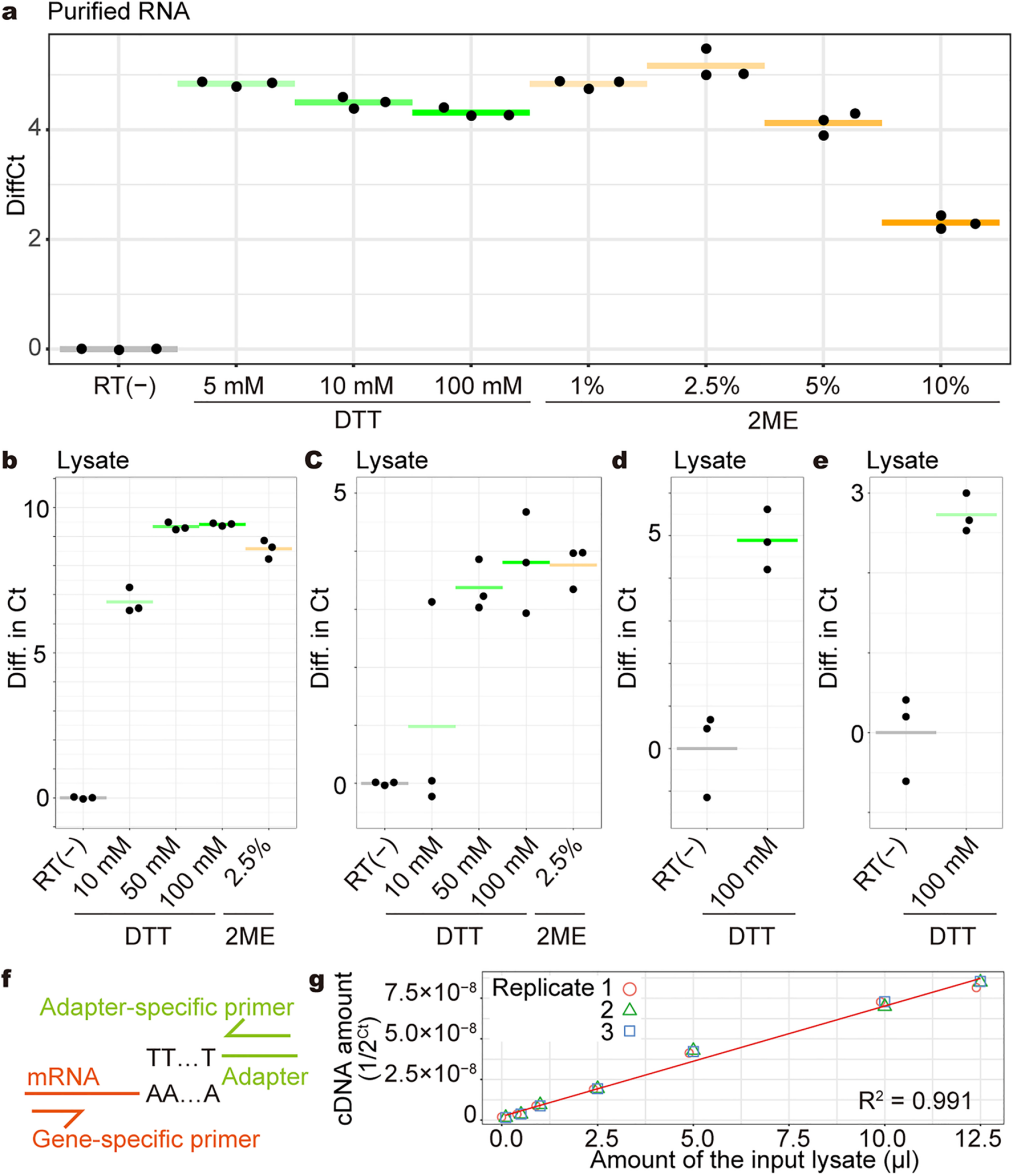
Quantification of cDNA synthesized by direct-lysate reverse transcription. a qPCR results for the *Os03g0836000* transcript in the cDNA synthesized from mixtures of purified *O. sativa* total RNA and the reducing reagents dithiothreitol (DTT) and 2-mercaptoethanol (2ME). The horizontal axis indicates the concentrations of the reducing regents in the RT reaction mixes. The vertical axis indicates the differences in Ct value compared to the RT(−) negative control. Each point indicates a value of each replicate. Each horizontal line indicates the average of each condition. **b**–**e** qPCR results for *AT3G18780* transcript (**b**), *Os03g0836000* transcript (**c**, **d**), and *HP620998.1* transcript (**e**) in cDNA synthesized from lysate of *A. thaliana* seedlings (**b**), the youngest fully-expanded leaves of *O. sativa* (**c**), *O. sativa* roots (**d**), and *T. aestivum* coleoptile (**e**). The horizontal axis indicates the concentrations of the reducing reagents in the homogenization buffers. The vertical axis indicates the differences in Ct value compared to the RT(−) negative control. Each point indicates a value of each replicate. Each horizontal line indicates the average of each condition. Same amount of total RNA was used as cDNA template according to RNA concentrations in lysates (250 ng of *A. thaliana* and *O. sativa* or 140 ng of *T. aestivum*). **f** A schematic diagram of the primer sets for cDNA detection.** g** qPCR results for *Os03g0836000* transcript in cDNA synthesized from lysate of *O. sativa* leaves. In the direct-lysate reverse transcription, 0.1, 0.5, 1, 2.5, 5, 10 and 12.5 µL of the lysate were used. Three technical replicates were prepared. The red line is a linear regression line

Next, we evaluated the efficiency of RT in the presence of reducing reagents. We added several different concentrations of DTT or 2ME to RT reaction mixes and performed RT of purified total RNA from *O. sativa*. Then, qPCR for the *Os03g0836000* transcript coding for an actin protein was conducted. In the manufacturer’s protocol for RTase, the addition of 5 mM DTT to RT reactions is recommended to protect the RT enzyme from oxidization, therefore 5 mM DTT was a positive control in this experiment. RT products with 5 mM DTT showed a 4.4 lower Ct value than the negative control (Fig. 2a). As the concentration of DTT increased, the Ct value slightly increased (Fig. 2a). The same tendency was observed for 2ME, but 10% 2ME drastically diminished the efficiency of the RT reaction (Fig. 2a). These results indicate that a high concentration of reducing reagent (e.g., 10% 2ME) inhibits the RT reaction, but a moderate concentration of reducing reagent does not.

To assess whether the addition of the reducing regents into homogenization buffer permits RT from plant lysates, we conducted direct lysate RT of *A. thaliana* seedlings, *O. sativa* leaf and root, and *T. aestivum* coleoptile, and quantified their actin cDNAs (*AT3G18780*, *Os03g0836000*, and *HP620998.1*, respectively) (Fig. 2b–e). Because the genomic DNA remained in the lysates, we conducted RT with dT primers harboring an adapter sequence at the 5′ end (Additional file 3: Table S1), followed by RT-qPCR with gene-specific and adapter-specific primers to exclusively detect cDNA synthesized by the direct-lysate RT (Fig. 2f). Amplification of the target cDNAs was observed in lysates containing reducing reagents (Fig. 2b–e) but not in the RT(−) negative controls. Consistent with the RNA degradation levels in the lysates (Fig. 2a), the lysate in 10 mM DTT buffer showed less cDNA production than the others (Fig. 2b, c). For all plant lysates, the 100 mM DTT buffer showed high production of actin cDNAs (Fig. 2b–e). In addition, to check the linearity of cDNA synthesis by direct-lysate RT, we conducted direct-lysate RT of varied amounts of *O. sativa* leaf lysate and quantified the cDNA using qPCR. The series of direct-lysate RT showed good linearity between the input amount of the lysate and the cDNA production (R^2^ = 0.991, Fig. 2g). Thus, Direct-RT buffer (100 mM DTT/90 mM Tris–HCl (pH 7.6)) was used for the subsequent direct-lysate RT experiments.

### Direct-lysate RT can produce cDNA comparable to RT from purified RNA

**Fig. 3:**
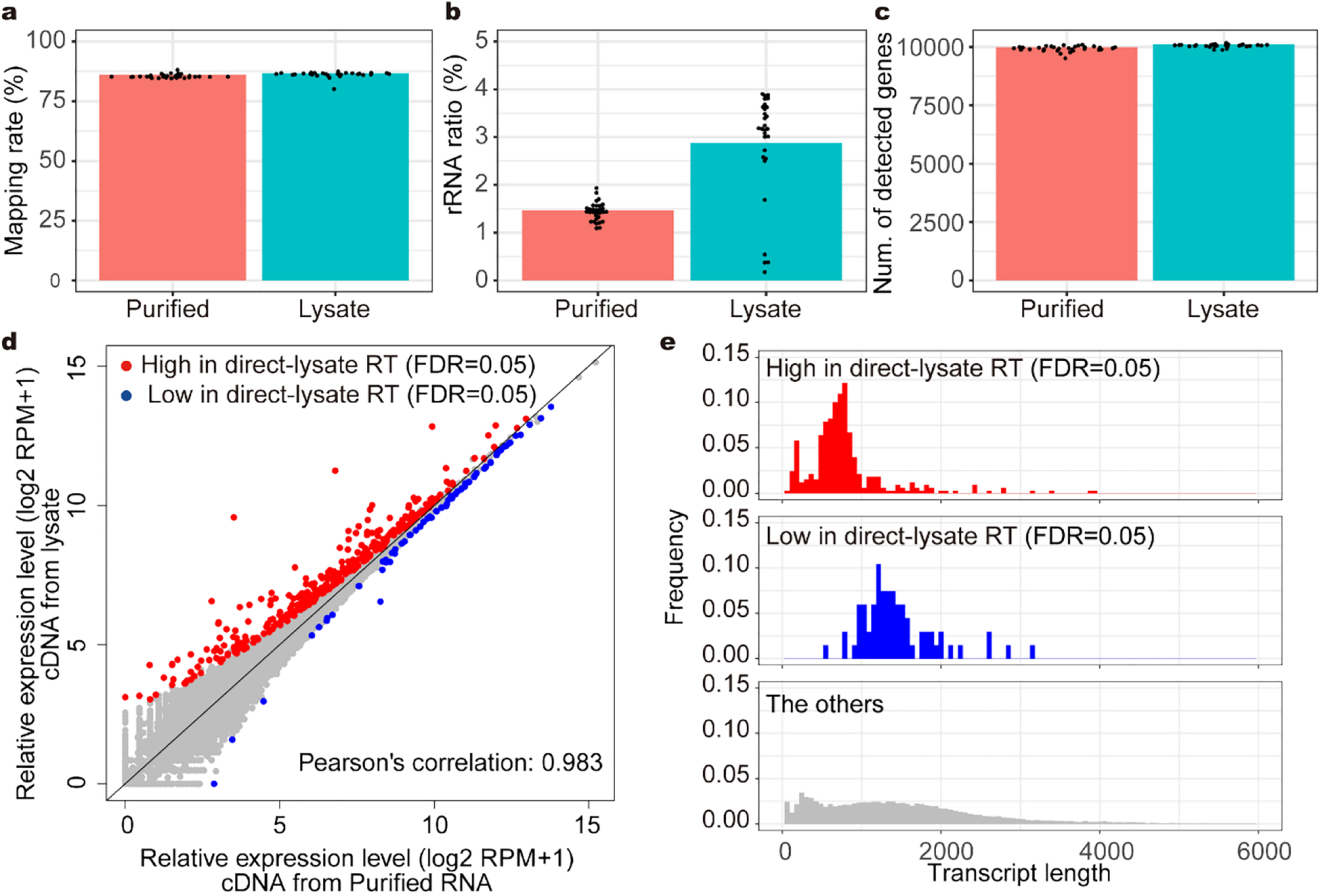
Direct-lysate reverse transcription generates cDNA comparable to that from reverse transcription of purified RNA. The results of 3′ RNA-Seq of purified RNA and lysate of *A. thaliana* seedlings. A total of 100,000 subsampled reads for each technical replicate were used for the following analyses. **a** Boxplot of the mapped read ratio to 100,000 reads. **b** Boxplot of the rRNA read ratio to 100,000 reads. **c** Boxplot of the number of detected genes. Each point indicates the value of each technical replicate. **d** Scatter plot of log2 RPM + 1 of each gene. The average of technical replicates was plotted. Differentially quantified genes (DQGs) were detected between RNA-Seq for purified RNA and lysate (FDR = 0.05). **e** Transcript length distribution of DQGs with higher rpm values in the method using lysate and other genes. Histograms from 0 to 6000 nt are shown

We examined bias in direct lysate RT compared with RT of purified total RNA. We compared the results of 3′ RNA-Seq of purified RNA and lysate. The lysate of *O. sativa* leaves was divided into two aliquots. One was purified and used for 3′ RNA-Seq library preparation, whereas the other was directly used for 3′ RNA-Seq library preparation. We sequenced 29 and 30 technical replicates for cDNA from the purified RNA and lysate, respectively. To remove noise derived from total read-number variation, we analyzed 100,000 reads subsampled from each technical replicate. The mapping rate onto the transcriptome reference in RNA-Seq of the lysate was approximately 1% higher than that of the purified RNA (Fig. 3a). In addition, the ratio of rRNA reads of the lysate was approximately 1.7% higher than that of the purified RNA (Fig. 3b). The numbers of detected genes in the purified RNA and lysate were almost the same (Fig. 3c). Comparison of each gene expression level showed that almost all genes were quantified equally in both methods, although 396 of 38,194 genes were differentially quantified (Fig. 3d). Among them, 329 genes were quantified at a higher amount in the lysate than in the purified RNA (Fig. 3d). The transcript length of the highly quantified genes tended to be short; the average length of the highly quantified transcripts was 799 nt, while the average length of all transcripts in the reference was 1537 nt (Fig. 3e). It is possible that direct-lysate RT could efficiently produce cDNA of short transcripts in the lysate of *A. thaliana* seedlings and/or that some of the short transcripts might be diminished through RNA purification. In addition, we conducted the same comparison of RNA-Seq of lysate and purified RNA from budding yeast and zebrafish larvae. Among 6713 genes in yeast, 37 and 48 genes were highly and lowly quantified, respectively, in RNA-Seq of the lysate (Additional file 4: Fig. S2a). Among 57,775 genes in zebrafish larvae, 6913 and 1048 genes were highly and lowly quantified in RNA-Seq of the lysate (Additional file 4: Fig. S2b). Unlike *A. thaliana* lysate, no bias in the transcript length was observed in either yeast or zebrafish lysates (Additional file 4: Fig. S2c, d). We concluded that the direct-lysate RT method can produce comparable or even higher amounts of cDNA to that synthesized from purified RNA, although RT efficiencies of some transcripts could be specifically altered by the composition of lysates.

### Direct-lysate targeted RNA-Seq (DeLTa-Seq)

**Fig. 4:**
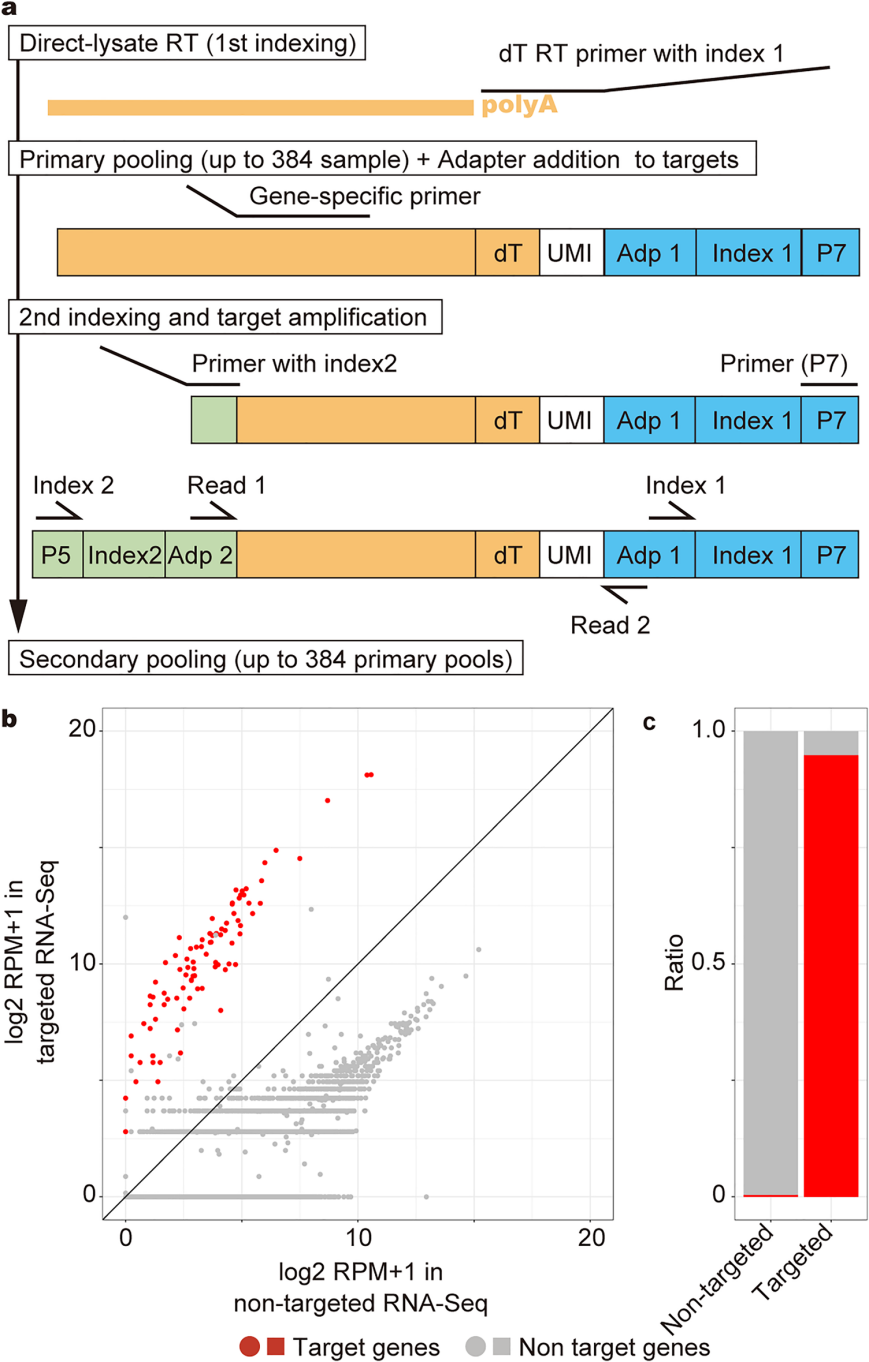
Successful application of DeLTa-Seq with *A. thaliana* seedlings. **a** A schematic diagram of DeLTa-Seq library preparation. P7 and P5 are adapter sequences binding with the oligonucleotides on the flow cells supplied by Illumina. Adp 1 and Adp 2 are adapter sequences for the sequencing. UMI indicates unique molecular identifier. **b** Scatter plot of log2 RPM + 1 values of each gene in targeted RNA-Seq and non-targeted RNA-Seq of lysate of *A. thaliana* seedlings. **c** The bar graph represents the ratio of reads corresponding to target and non-target genes in targeted RNA-Seq and non-targeted RNA-Seq

To develop a cost-effective and accurate gene quantification method applicable with direct-lysate RT, we attempted to utilize targeted amplicon RNA-Seq [28]. We designed gene-specific primers for all *A. thaliana* genes (https://sites.google.com/view/delta-seq/). Here, we focused on the 100 marker genes used in a previous study [36]. We integrated the PCR-based targeted RNA-Seq with direct-lysate RT in a novel technique named “Direct-Lysate Targeted RNA-Seq” (DeLTa-Seq) (Fig. 4a). First, 1st index 1 was added by direct-lysate RT with an oligo-dT primer harboring a unique molecular identifier (UMI), followed by sample pooling (up to 384 samples). Second, part of the adapter sequence was added to the cDNA of target genes using gene-specific primers harboring a part of the adapter sequence. Third, 2nd indexing and target amplification were conducted using PCR. If needed, up to 384 libraries can be pooled, which enables simultaneous sequencing of up to 147,456 samples. To evaluate target enrichment by DeLTa-Seq, we prepared two libraries, non-targeted RNA-seq and targeted RNA-Seq (DeLTa-Seq), from the same lysate of *A. thaliana* seedlings. Sequencing of these libraries generated 6,694,751 reads of the non-targeted RNA-Seq and 203,450 reads of DeLTa-Seq. All target genes were quantified at higher levels in targeted RNA-Seq than in non-targeted RNA-Seq (Fig. 4b). The ratio of reads corresponding to the target genes increased from 0.45% in the non-targeted RNA-Seq to 94.9% in the targeted RNA-Seq; more than 200-fold enrichment for the target cDNA was achieved even with direct-lysate RT (Fig. 4c).

### Targeted RNA-Seq works with a low amount of input RNA

**Fig. 5:**
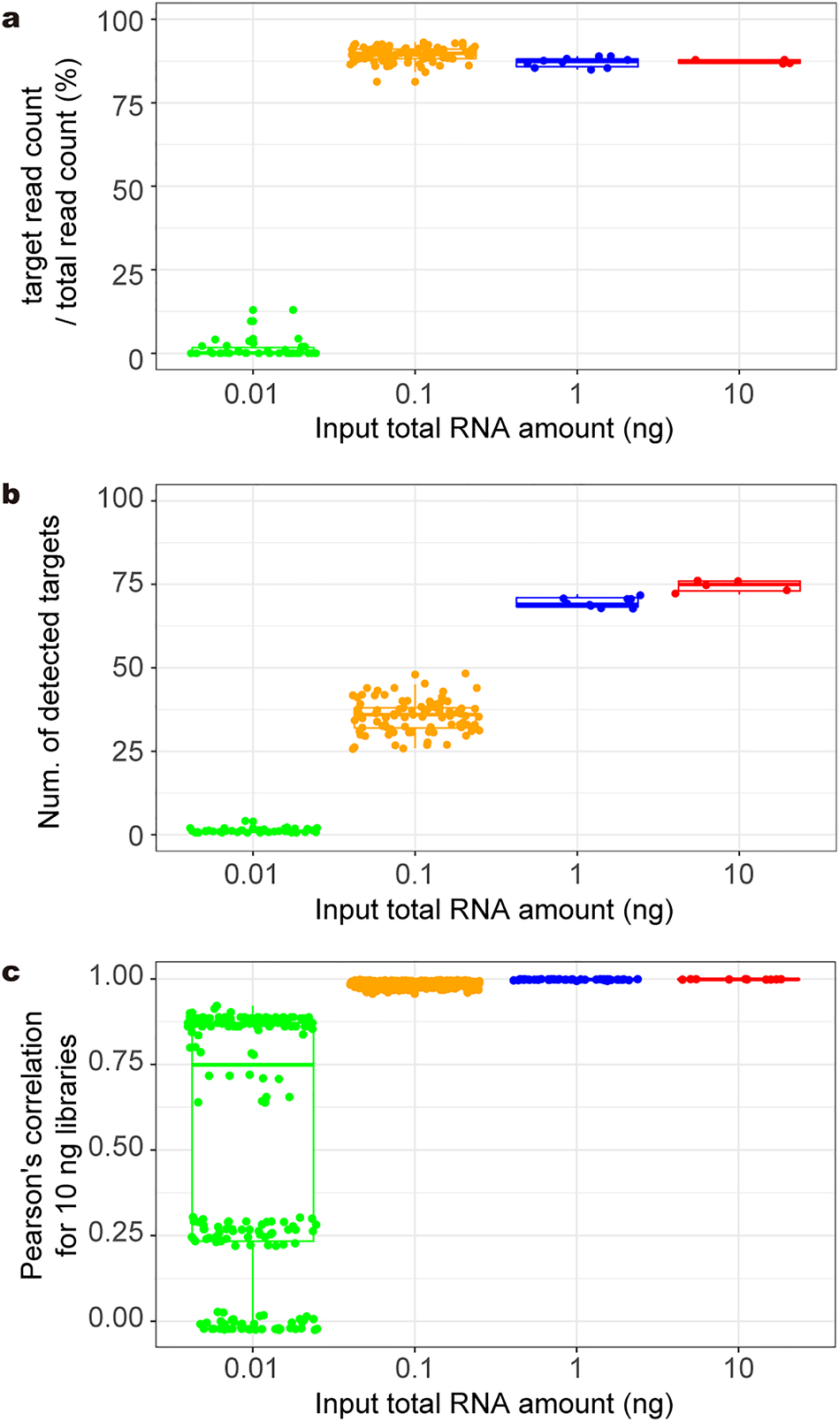
Required amount of input RNA for targeted RNA-Seq. **a**–**c** The result of targeted RNA-Seq using 0.01 ng (n = 96), 0.1 ng (n = 96), 1 ng (n = 10), and 10 ng (n = 5) of purified *A. thaliana* RNA. A subsample of 10,000 reads from each library was used for the analyses. Boxplots of the ratio of target reads per total read (**a**), the numbers of detected target genes (**b**) and Pearson’s correlation coefficients for the results of each input amount compared to that of 10 ng RNA (**c**). Each point indicates the value of each technical replicate

Next, we examined the required amount of input RNA for targeted RNA-Seq. We prepared targeted RNA-Seq libraries from 0.01, 0.1, 1, and 10 ng of the same purified *A. thaliana* total RNA with 96, 96, 10, and 5 technical replicates, respectively. After sequencing, 10,000 reads were subsampled from each replicate. The mean ratios of the target reads to the subsampled reads were 89.5%, 87.1%, and 87.3% in the libraries with 0.1, 1, and 10 ng inputs, respectively, compared to 1.31% in the libraries with 0.01 ng input (Fig. 5a). While 86 genes were detected in total, the number of detected target genes was saturated in the libraries with more than 0.1 ng input (69.6 and 74.4 genes on average with 1 and 10 ng input, respectively), whereas very few target genes (1.26 genes on average) were detected in the libraries with 0.01 ng input (Fig. 5b). Pearson’s correlation coefficient of log2 RPM + 1 of the target genes in each library to each 10-ng-input library was also saturated in the libraries with more than 0.1 ng input (Fig. 5c). Based on these results, targeted RNA-Seq requires around 1 ng of total RNA input, and would be applicable for small tissues.

### Targeted RNA-Seq improved the reproducibility and cost of gene quantification

**Fig. 6:**
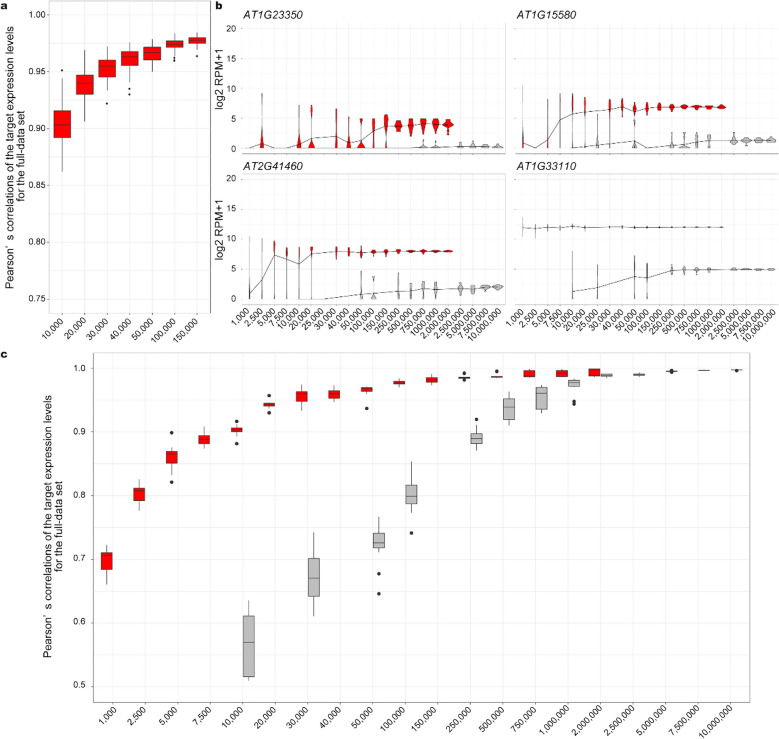
Reproducibility and required UMI counts for targeted and non-targeted RNA-Seq. **a** Plot of Pearson’s correlation coefficients of the target log2 RPM + 1 between each of the subsampled 96 technical replicates and the full targeted RNA-Seq dataset. The replicates were prepared using a single tube of purified RNA from *A. thaliana* seedlings. **b** Plots of examples of the target log2 RPM + 1 in each size of subsampled sets from 12 pseudo-technical replicates of targeted and non-targeted RNA-Seq results. The same purified RNA was used for the preparation of 96 technical replicates of non-targeted RNA-Seq. Then, groups of 8 RNA-Seq results were merged, resulting in 12 pseudo-technical replicates. **c** Plot of Pearson’s correlation coefficients of the target log2 RPM + 1 between each subsampled set from the 12 pseudo-technical replicates and the full dataset of targeted and non-targeted RNA-Seq. **a**–**c** Red indicates targeted RNA-Seq, gray indicates non-targeted RNA-Seq, horizontal axis labels indicate the numbers of subsampled UMI counts

To examine the reproducibility and performance of mRNA quantification by targeted RNA-Seq, we prepared 96 technical replicates of both targeted and non-targeted RNA-Seq libraries using a single tube of RNA from *A. thaliana* seedlings and sequenced them using a HiSeq. In our targeted RNA-Seq, we used 10-base unique molecular identifiers (UMI), which can theoretically distinguish a million RNA molecules from each target transcript [30, 37]. All reads were used after UMI preprocessing, resulting in 96 technical replicates of non-targeted RNA-Seq of 1786180 UMI counts on average and 96 technical replicates of targeted RNA-Seq of 344215 UMI counts on average. The average UMI conversion efficiencies were 0.805 in non-targeted RNA-Seq and 0.434 in targeted RNA-Seq (Additional file 4: Fig. S3). We merged all UMI counts from targeted and non-targeted RNA-Seq (33044648 and 171473284 UMI counts, respectively) to be used as ‘full data’. First, to evaluate the reproducibility of our targeted RNA-Seq protocol, we subsampled from 10,000 to 150,000 UMI counts from each technical replicate of targeted RNA-Seq. Pearson’s correlation coefficients of log2 RPM + 1 of the target genes in each subsampled dataset for the full data were calculated. With 150,000 UMI counts, 96 replicates showed high reproducibility (the average of Pearson’s correlation coefficient for the full data was 0.977) (Fig. 6a). Next, to examine quantification performance, we merged every eight technical replicates of non-targeted and targeted RNA-Seq into one, resulting in 12 pseudo-technical replicates. Then, we subsampled from 1000 to 2,000,000 UMI counts of targeted RNA-Seq and from 10,000 to 10,000,000 UMI counts of non-targeted RNA-Seq from each pseudo-technical replicate. The reproducibility of quantification of each target gene was almost saturated at 250,000 UMI counts in the targeted RNA-Seq, and at 5,000,000 UMI counts in the non-targeted RNA-Seq (Fig. 6b). Similarly, Pearson’s correlation coefficients of log2 RPM + 1 of the target genes in each subsampled dataset for the full data were saturated at 250,000 and 5,000,000 UMI counts in the targeted and non-targeted RNA-Seq, respectively (Fig. 6c). Thus, targeted RNA-Seq (DeLTa-Seq) would enable us to conduct cost-effective and accurate quantification of target gene expression by reducing the required amount of sequence data.

### DeLTa-Seq revealed temperature-dependent effects of jasmonic acid and salicylic acid

**Fig. 7:**
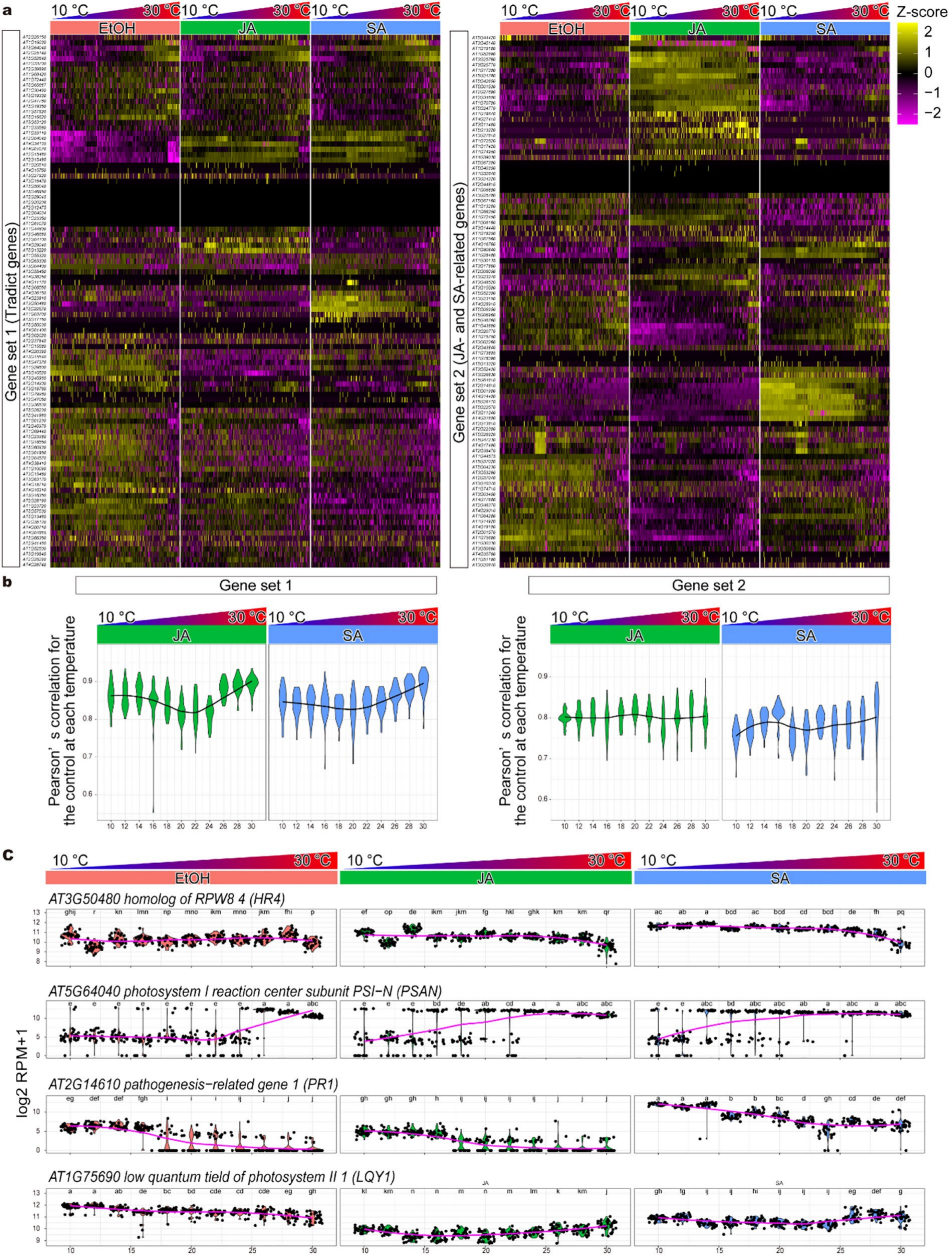
Effects of jasmonic acid and salicylic acid on *A. thaliana* seedlings at ambient temperatures. **a** Heatmaps of scaled RPM of targets genes in *A. thaliana* seedlings treated with EtOH, jasmonic acid, and salicylic acid. **b** Plots of Pearson’s correlation coefficients of target log2 RPM + 1 between each sample and average expression of EtOH-treated samples at the same temperatures. **c** Plots of examples of the target log2 RPM + 1 in EtOH-, jasmonic-acid- and salicylic-acid-treated *A. thaliana* seedlings. Magenta lines represent the trend curves of each target log2 RPM + 1 with the ambient temperatures. Alphabets indicate results of Tukey's test (FDR = 0.05)

Finally, we examined the effects of JA and SA on *A. thaliana* seedlings under different temperatures with DeLTa-Seq. Seven days after sowing, *A. thaliana* seedlings were treated with 0.33 µM JA, 0.33 µM SA, and 1% EtOH (control) at every 2 °C rise from 10 °C to 30 °C for 6 h. These treatments were conducted with 32 replicates. A total of 1,056 samples were analyzed with DeLTa-Seq. In addition to Tradict gene sets [36] (gene set 1), we used “gene set 2”, which is composed of 98 genes related to JA and SA pathways (Additional file 3: Table S4). A total of 44,948 and 47,185 UMI counts for 65.9 and 64.1 target genes, on average, were obtained by DeLTa-Seq analysis of gene set 1 and set 2, respectively (Fig. 7a; Additional files 1, 2: Data 1 and 2). DeLTa-Seq captured common (e.g. *AT2G04040.1*) and specific downstream (e.g. *AT1G44800.1*) genes related to JA and SA as well as temperature-dependent changes in gene expression (Fig. 7a). Interestingly, correlation analysis between average log2 RPM + 1 of the control at each temperature and each sample showed that the effects of JA and SA decreased as the temperature increased (Fig. 7a, b). In gene set 1, which could represent genome-wide transcriptional states of *A. thaliana* [36], correlation coefficients for the control at each temperature remarkably increased at 26 °C in JA-treated plants, while correlation coefficients gradually increased at temperatures greater than 22 °C in SA-treated plants (Fig. 7b); this shows the difference in sensitivity to temperature of JA- and SA-induced responses. In gene set 2, correlation coefficients for the control at each temperature did not change in JA-treated plants, while they increased at ≥ 22 °C in SA-treated plants (Fig. 7b), indicating that SA-pathway is more sensitive to temperature than JA-pathway. In addition, DeLTa-Seq analysis revealed various patterns of gene expression in response to temperature changes under JA and SA treatments. For example, *AT3G50480* (*HR 4*) of gene set 1 was upregulated under SA but not under JA treatment, and the effects of SA gradually decreased at ≥ 26 °C and disappeared at 30 °C (Fig. 7c). In the control, *AT5G64040* (*PSAN*) of gene set 1 was drastically up-regulated at ≥ 26 °C, while it was up-regulated in JA- and SA-treated plants even at ≥ 14 °C (Fig. 7c), suggesting that JA and SA can affect temperature-response of gene expression. *AT2G14610* (*PR 1*) of gene set 2, which is a well-established marker gene for immunity controlled by SA [38], was down-regulated at ≥ 18 °C in the control and JA-treated plants (Fig. 7c). In SA-treated plants, this trend was also observed, but the expression was still higher even at 30 °C than that in the control and JA-treated plants at 10 °C (Fig. 7c). This could contribute to pathogen resistance in SA-treated plants under high temperature. Finally, the expression of *AT1G75690* (*LQY1*), which codes for a Zn finger protein involved in repair and reassembly of PSII complexes [39], gradually decreased as the temperature increased (Fig. 7). Conversely, its expression gradually increased in both JA- and SA-treated plants as the temperature increased; however, its overall expression level was lower in JA-treated plants than the control (Fig. 7). These results show that JA and SA switch the negative effect of temperature to a positive effect for *LQY1* expression.

## Discussion

Direct-lysate RT is utilized in analyses of cultured animal cells to avoid the labor required for RNA purification, followed by gene expression analysis techniques such as RT-qPCR and RNA-Seq [21, 40]. In these direct-lysate RT methods, RNase inhibitors are usually added to the lysis buffers to inhibit RNA degradation by intrinsic RNase. Commercial RNase inhibitors are expensive and therefore it is not realistic to use them in large-scale preparation of lysates from crude plant and animal tissues. Notably, we demonstrated that these methods cannot be applied to plant and animal tissues (Additional file 4: Fig. S1). In this study, we developed a Direct-RT buffer that facilitates cost-effective direct-lysate RT of crude plant, yeast, and animal samples. DTT can reduce the disulfide bonds required for the activity of RNase in lysates [24]. Because direct-lysate RT does not involve the loss of RNA associated with purification methods, this technique is also beneficial for gene expression analysis in small tissues from which it is difficult to purify enough RNA for the experiments. Furthermore, DTT may contribute to long-term maintenance of endogenous RNase inhibitor activity during storage at − 20 °C [23, 41]. Thus, our Direct-lysate RT technique will be useful not only for gene expression analysis but also for RNA storage.

Comparison of the non-targeted RNA-Seq results from purified RNA and lysate revealed that some transcripts were differentially reverse transcribed in the direct-lysate RT compared to the RT of the purified RNA (Fig. 3d; Additional file 4: Fig. S2). For *A. thaliana* seedlings, the amount of cDNA generated from rRNA in the direct-lysate RT was double that in the RT with purified RNA, but less than 4% of the rRNA amount (Fig. 3b). Previous studies have shown that nonspecific priming from the A-rich region of rRNA with the dT primer occurs in the RT step [19, 42]. Considering that the salt concentration will be higher in the lysate than in the purified RNA solution, and that salt increases the base-pairing stability of nucleic acids [43], nonspecific priming by the dT primer against non-polyA RNA such as rRNA would occur in direct-lysate RT more than in RT with purified RNA. In addition, direct-lysate RT in the lysate of *A. thaliana* seedlings might produce more cDNA of relatively short transcripts (Fig. 3e). Given that numerous transcripts were differentially quantified in the zebrafish lysate (Additional file 4: Fig. S2), it is possible that the composition of the lysates affected the binding between the transcripts and the magnetic beads, causing bias in the RNA purification step. We recommend comparison of the RNA-Seq of a lysate with that of purified RNA as a pilot experiment.

In this study, we examined how much input RNA was required for accurate gene quantification. At least 0.1 ng total RNA per sample was required for stable quantification of the target genes (Fig. 5b, c), but targeted-RNA-Seq with 0.1 ng total RNA resulted in abundant useless reads (Fig. 5a). The quantity of the 100 target RNAs in 0.01 ng of total RNA could be less than 0.1 pg (1% of total RNA), which is equal to the mRNA amount in one cell [44], and this resulted in few detected targets (Fig. 5b), as observed in single-cell RNA-Seq. Single-cell RNA-Seq can only capture a small fraction of the transcriptome of each cell and the count of most genes is zero—a phenomenon known as dropouts [45]. Considering that one cell contains 10 pg of total RNA [44], the requirement of DeLTa-Seq will be met by small tissue samples consisting of around 100 cells.

A high resolution of time, space, perturbation, and stochastic variation is required to understand complex biological systems via transcriptome analysis. It is difficult to apply a non-targeted RNA-Seq strategy to thousands of samples because of the associated sequencing costs. Although the required read counts for robust quantification by targeted RNA-Seq depend on the expression levels of target transcripts, our study indicated that 150,000 UMI counts could be enough for targeted RNA-Seq to quantify target transcripts (Pearson’s correlation coefficient > 0.98) (Fig. 6). Given the high throughput of recent Illumina DNA sequencers and our UMI conversion efficiencies (Additional file 4: Fig. S3), we can sequence thousands of samples per lane, resulting in cost-effective quantification of genes of interest. For example, with the HiSeq 4000 system (Illumina, San Diego, CA, USA), which can generate 300 million reads per lane, two thousand samples can be sequenced simultaneously. These advantages, coupled with the lack of any RNA purification step, mean that DeLTa-Seq can drastically reduce cost and labor involved in mRNA quantification.

DeLTa-Seq enabled us to examine the effects of JA and SA under varying temperature conditions. Various gene expression patterns were revealed under JA- and SA-treatments and different temperature conditions (Fig. 7). SA and JA not only additively up- or down-regulated the gene expression but also acted synergistically in response to temperature as observed for *AT5g64040* and *AT1G75690* (Fig. 7c). Interestingly, our results showed that genes downstream of SA are less tolerant to high temperature than those downstream of JA (Fig. 7b). Although JA and SA are thought to function antagonistically [46], our results showed that the up-regulation of common downstream genes, such as *AT1G80840* and *AT1G28480*, which codes for a pathogen-induced transcription factor [47, 48] and a glutaredoxin family protein regulating protein redox state and involved in JA/SA cross-talk [49], respectively was diminished at high temperature by SA but not by JA (Fig. 7a; Additional file 2: Data 2). There is a possibility that JA treatment could partially compensate for the defense response that is diminished by an incompetent SA pathway under high temperature. In addition to defense responses, it is known that both JA and SA can contribute to the improvement of basal thermotolerance [50]. *AT5g64040*, which codes for photosystem I (PS I) reaction center subunit PSAN, was up-regulated at ≥ 26 °C in the control (Fig. 7c) and at ≥ 16 °C under JA- and SA-treatments. PSNA is involved in electron transfer from plastocyanin to P700 + , the oxidized reaction center of PS I, thus enhancing NADPH production and yield of photosynthesis [51]. Considering that heat stress enhances electron transfer to PS I in various photosynthetic organisms [52], up-regulation of *AT5g64040* prior to heat-stress could be important in increasing basal thermotolerance in JA- and SA-treated plants. As discussed above, this study provided novel insights into the interactions between JA and SA pathways and temperature. Further studies will reveal the detailed mechanisms and biological roles of JA and SA under varying temperature conditions.

## Conclusions

DeLTa-Seq can be applied to animals, plants, and microorganisms and is useful not only for large-scale studies handling thousands of samples, such as field transcriptome and chemical screening, but also for in-depth studies of specific biological systems. For example, a researcher can conduct a limited number of comprehensive RNA-Seq analyses and find genes of interest. DeLTa-Seq can measure the expressions of the genes of interest in many samples required for in-depth analysis. Notably, in our protocol, the same cDNA can be shared for both non-targeted and targeted RNA-Seq. This advantage enables the researcher to select non-targeted or targeted RNA-Seq according to the aim of each round. DeLTa-Seq will achieve cost-effective and accurate quantification of these genes in numerous samples. The DeLTa-Seq technique will open up a novel field of research in both plant and animal biology.

## Methods

### Plant materials

Seeds of *Oryza sativa* L. *japonica* ‘Koshihikari’ were sown in nursery trays then cultivated for two weeks under a 16-h light, 30 °C /8-h dark, 20 °C cycle.

Seeds of *Arabidopsis thaliana* L. (Col-0, CS70000) were sterilized in 90% EtOH. Then, they were sown on 0.5 × Murashige and Skoog medium with 0.25% gellan gum (Wako, Osaka, Japan), 0.05% (v/v) PPM-100 (Plant Cell Technology, Washington, D.C., USA), 1.25 mM MES-KOH (pH 5.7), and 0.5% sucrose followed by cultivation for 21 days at 20 °C under a 16-h light/8-h dark cycle.

Seeds of *Triticum aestivum* L. ‘Chinese Spring’ were sown on 200 μL zirconia beads YTZ-1 (AS-ONE, Osaka, Japan) with 110 μL nuclease-free water in 2 mL microtubes LT-0200 (INA OPTICA, Tokyo, Japan), followed by cultivation for 3 days under 24-h dark at 4 °C and then 8 days under 24-h light at 20 °C.

### Budding yeast material

*S. cerevisiae* BY4742 strain was cultured in synthetic complete (SC) medium at 28 °C. Yeast in the exponential phase was used in this study.

### Animal material

Fertilized eggs of zebrafish (*Danio rerio*) were obtained from a cross of lab stock AB provided by the Zebrafish International Resource Center (ZIRC) at the University of Oregon. They were maintained at 28 °C under dark conditions until sampling. Larvae were sacrificed at 48 h post-fertilization.

### Preparation of purified *O. sativa *and *A. thaliana* RNA

Two weeks after seeding, the youngest fully expanded leaves of *O. sativa* were frozen in liquid nitrogen and stored at -80 °C prior to RNA isolation for RNA-Seq. Frozen samples were homogenized with zirconia beads YTZ-4 (AS-ONE, Osaka, Japan) using TissueLyser II (Qiagen, Hilden, Germany), and total RNA was extracted using the Maxwell 16 LEV Plant RNA Kit (Promega, Madison, WI, USA) and the Maxwell 16 Automated Purification System (Promega). The concentration of RNA was measured using a QuantiFluor RNA System (Promega) and Quantus Fluorometer (Promega, Madison, WI, USA).

Seven days after seeding, bulked seedlings of *A. thaliana* were homogenized with zirconia beads YTZ-4 using a TissueLyser II (Qiagen, Hilden, Germany), and total RNA was extracted using the Maxwell 16 LEV Plant RNA Kit (Promega, Madison, WI, USA) and the Maxwell 16 Automated Purification System (Promega, Madison, WI, USA). The RNA concentration was measured using a Quantus Fluorometer (Promega, Madison, WI, USA).

### Preparation of lysate

A maximum of 9 seedlings of *A. thaliana* were homogenized with zirconia beads YTZ-4 using a TissueLyser II in 400 µL of 90 mM Tris–HCl (pH 7.6) containing reducing regents (10, 50, or 100 mM DTT or 2.5% 2ME), CL buffer [50 mM Tris–HCl pH 7.6, 0.1% Igepal CA-630, 150 mM NaCl, and 1 mM DTT] [23] or CellAmp Processing Buffer (TaKaRa), or SuperPrep Lysis Solution (TOYOBO, Osaka, Japan). DTT solution was freshly prepared or stored at − 20 °C. According to the SuperPrep manufacturer’s instructions, 76 µL Stop Solution (TOYOBO) and 4 µL RNase inhibitor (TOYOBO) were added following incubation for 5 min at 22 °C.

Each youngest fully expanded leaf of *O. sativa* was frozen in liquid nitrogen and stored at -80 °C until homogenization. Each frozen sample was homogenized with zirconia beads YTZ-4 using a TissueLyser II. Then, 400 µL of the same buffers used for *A. thaliana* seedlings were added, and immediately, the samples were mixed well by vortexing. According to the SuperPrep manufacturer’s instructions, 76 µL Stop Solution and 4 µL RNase inhibitor were added following incubation for 5 min at 22 °C.

Each coleoptile with the first leaf of *T. aestivum* was frozen in liquid nitrogen and stored at − 80 °C until homogenization. Each frozen sample was homogenized with zirconia beads YTZ-4 using a TissueLyser II. Then, 400 µL of Direct-RT buffer was added, and immediately, the samples were mixed well by vortexing.

*S. cerevisiae* in the exponential phase was homogenized with zirconia beads YTZ-1 and MS-100R (TOMY, Tokyo, Japan) in 350 µL of 90 mM Tris–HCl (pH 7.6) containing 100 mM DTT.

Three *D. rerio* larvae were homogenized in 50 µL of the same buffers used for *A. thaliana* seedlings with a Biomasher II (Nippi, Tokyo, Japan). According to the SuperPrep manufacturer’s instructions, 9.5 µL Stop Solution and 0.5 µL RNase inhibitor were added following incubation for 5 min at 22 °C.

The concentration of RNA in each lysate was measured using the Quant-iT RNA Assay Kit (Thermo Fisher Scientific, Waltham, MA, USA) and the Infinite M1000 PRO plate reader (TECAN, Zurich, Switzerland).

### Purification of RNA from lysates

The lysate was incubated for 1 h at 22 °C. Then, a 2.5 × volume of AMpure XP beads (Beckman Coulter, Brea, CA, USA) was added to the lysate and the mixture was purified according to the manufacturer’s instructions. The RNA was eluted with an equal volume of RNase-free water to the lysate. One microliter of the purified RNA solution was electrophoresed using a Bioanalyzer 2100 with Agilent RNA nano or pico kit (Agilent Technologies, Santa Clara, CA, USA) to check for quality.

### Direct-lysate reverse transcription

RT reactions were conducted with 1 μL of 2 μM RT primer (CAGAAGACGGCATACGAGATGCGTCTACGTGACTGGAGTTCAGACGTGTGCTCTTCCGATCNNNNNNTTTTTTTTTTTTTTTTTTV), 0.4 μL of 25 mM dNTP (Advantage UltraPure dNTP Combination Kit; TaKaRa), 4.0 μL of 5 × SSIV Buffer (Thermo Fisher Scientific), 0.1 μL of SuperScript IV reverse transcriptase (200 U/μL, Thermo Fisher Scientific), lysate, and nuclease-free water to make a volume of 20 μL. In order to suppress production of cDNA from rRNA [19], reverse transcription was carried out at 65 °C for 10 min, followed by incubation at 80 °C for 15 min to inactivate the enzyme.

### Quantitative PCR analysis of cDNA (RT-qPCR)

For Fig. 2, RT was performed using 250 ng (*A. thaliana* and *O. sativa*) or 140 ng (*T. aestivum*) of total RNA in the lysate. Then, the *A. thaliana* and *O. sativa* reaction mix was diluted 20 times with nuclease-free water. The *T. aestivum* reaction mix was used for subsequent experiments without dilution. The cDNA amount of *AT3G18780*, *Os03g0836000*, or *HP620998.1* was measured by qPCR analysis. The composition of the qPCR mixture is described below: 2 µL diluted cDNA solution, gene-specific primer (CTTGCACCAAGCAGCATGAA for *AT3G18780* or GTGTGTCGGTACTTTCGTCG for *Os03g0836000*, TGACCGTATGAGCAAGGAG for *HP620998.1*) [53], P7 primer for the adapter sequence added in the RT step (CAAGCAGAAGACGGCATACGAGAT), and 5 µL of KAPA SYBR FAST qPCR Master Mix (2 ×) (KAPA BIOSYSTEMS, Wilmington, MA, USA). qPCR was conducted using a LightCycler 480 System II (Roche Diagnostics, Basel, Switzerland) with the following program: enzyme activation at 95 °C for 3 min, 40 cycles of 95 °C for 3 s and 60 °C for 30 s for amplification.

### Non-targeted RNA-Seq

For Fig. 3, Non-targeted RNA-Seq was conducted according to the Lasy-Seq ver. 1.1 protocol [19] (https://sites.google.com/view/lasy-seq/). RT reactions were conducted with 1 μL of 2 μM RT primer (CAGAAGACGGCATACGAGATxxxxxxxxGTGACTGGAGTTCAGACGTGTGCTCTTCCGATCNNNNNNTTTTTTTTTTTTTTTTTTVT (xxxxxxxx indicates index sequences for multiplex sequencing; see Additional file 3: Table S1), 0.4 μL of 25 mM dNTP, 4.0 μL of 5 × SSIV Buffer, 2.0 μL of 100 mM DTT, 0.1 μL of SuperScript IV reverse transcriptase, template RNA (lysate or purified RNA solution) and nuclease-free water to make a volume of 20 μL. In order to suppress production of cDNA from rRNA [19], reverse transcription was carried out at 65 °C for 10 min, followed by incubation at 80 °C for 15 min to inactivate the enzyme. Then, all RT mixtures of samples were pooled and purified with Wizard SV Gel and PCR Clean-Up System (Promega, Madison, WI, USA) according to the manufacturer’ s instructions, except for the use of 80% EtOH instead of Membrane Wash Solution. The purified cDNA was eluted with 30 μL of nuclease-free water. Second strand synthesis was conducted on the pooled samples (30 μL) with 4 μL of 10 × blue buffer (Enzymatics, Beverly, MA, USA), 2 μL of 2.5 mM dNTP (Advantage UltraPure dNTP Combination Kit), 1 μL of 100 mM DTT, 1 μL of RNase H (5 U/μL, Enzymatics), and 2 μL of DNA polymerase I (10 U/μL, Enzymatics). The reaction was conducted at 16 °C for 2 h and kept at 4 °C until the next reaction. To avoid the carryover of large amounts of rRNAs, the mixture was treated with 1 µL of RNase T1 (1 U/µL, Thermo Fisher Scientific) and 1 µL of RNase A (10 ng/µL, NIPPON GENE, Tokyo, Japan) at 37 °C for 5 min and then stored at 4 °C until the next reaction. Purification was conducted with a 0.8 × volume of AMpure XP beads according to the manufacturer’s instructions, followed by elution with 36 µL nuclease-free water. Fragmentation, end-repair, and A-tailing were conducted in a mixture of 17.5 µL of dsDNA, 2.5 µL of 10 × Fragmentation Buffer (Enzymatics), and 5 µL of 5 × WGS Fragmentation Mix (Enzymatics). The mixture was kept at 4 °C until the next reaction. The reaction was conducted at 4 °C for 1 min, 32 °C for 7 min, 65 °C for 30 min, and then the mixture was kept at 4 °C until the next reaction. The adapter for the next ligation step was prepared by annealing 100 mM of A*C*C*GAGATCTACACACTCTTTCCCTACACGACGCTCTTCCGA*T*C*T and /5Phos/G*A*T*CGGAAGAGCGTCGTGTTAAATGTA*T*A*T (* signifies a phosphorothioate bond, /5Phos/ signifies a phosphorylation) using a thermal cycler with the following program: 95 °C for 2 min, gradually cooled to 45 °C (0.1 °C/s), followed by 45 °C for 5 min. The following were added to the above reaction mixture: 10 µl of 5 × Ligation buffer (Enzymatics), 8 µL of nuclease-free water, and 2 µL of 4 mM annealed adapter, followed by 5 µL of T4 DNA ligase (Enzymatics). The ligation was conducted at 20 °C for 15 min. The adapter-ligated DNA was purified twice with 0.8 × volume of AMpure XP beads, followed by elution with 17 µL of nuclease-free water. To optimize the PCR cycle for library amplification, qPCR was conducted with 3.5 µL of the adapter-ligated DNA, 5 µL of KAPA HiFi HotStart ReadyMix (KAPA BIOSYSTEMS), 0.5 µL of EvaGreen dye (20 × in water; Biotium, Fremont, CA, USA), 0.5 µL of 10 µM 5 × WGS_Pr_R1-5′ primer (AATGATACGGCGACCACCGAGATCTACACTCGTCGGCAGCGTC) and 0.5 µL of 10 µM PCR_P7 primer (CAAGCAGAAGACGGCATACGAGAT). The reaction was carried out using a LightCycler 480 II at 95 °C for 5 min, then 30 cycles of 98 °C for 20 s, 60 °C for 15 s, 72 °C for 40 s, followed by 72 °C for 3 min, and then held at 4 °C. The optimal number of PCR cycles was the cycle corresponding to the middle of the exponential phase. Then, the library was amplified with 12 µL of the adapter-ligated DNA, 15 µL of KAPA HiFi HotStart ReadyMix, 1.5 µL of 10 µM 5 × WGS_Pr_R1-5′ primer and 1.5 µL of 10 µM PCR_P7 primer. The reaction was carried out at 95 °C for 5 min, then the optimal number of cycles of 98 °C for 20 s, 60 °C for 15 s, 72 °C for 40 s, followed by 72 °C for 3 min, and then held at 4 °C. The amplified library was purified with an equal volume of AMpure XP beads and eluted with 15 µL of nuclease-free water. One microliter of the library was electrophoresed using a Bioanalyzer 2100 with Agilent High Sensitivity DNA kit to check for quality. Sequencing of 50-bp single-ends was performed using a HiSeq (Illumina, San Diego, CA, USA).

### Design of gene-specific primers for *A. thaliana*

We designed gene-specific primers for *A. thaliana* cDNA sequences using Primer3 [54], with the following parameters: Concentration of Monovalent Cations = 40 mM, Concentration of Divalent Cations = 6 mM, Annealing Oligo Concentration = 25 nM, Primer Size Min/Opt/Max = 20/23/25, Primer Tm Min/Opt/Max = 60/65.5/70, Primer GC% Min/Opt/Max = 30/50/55, Max Tm Difference = 5, Product Size Ranges = 289–889, Number To Return = 5, PRIMER_RIGHT_INPUT = CAAGCAGAAGACGGCATACGAGAT, PRIMER_MAX_END_STABILITY. Candidate gene-specific primers were then concatenated as follows: TCGTCGGCAGCGTCAGATGTGTATAAGAGACAG + gene-specif-primer. All candidate gene-specific primers for all *A. thaliana* genes are available at https://sites.google.com/view/delta-seq/).

### Direct-lysate targeted RNA-Seq (DeLTa-Seq)

For Fig. 4, RT reactions and sample pooling were conducted according to the above protocol for non-targeted RNA-Seq, followed by elution of pooled cDNA with 40 µL of nuclease-free water. The eluted cDNA (20 µL) was treated with 1 µL of RNase T1 and 1 µL of RNase A. The reaction was conducted at 37 °C for 5 min and then kept at 4 °C until the next reaction. Purification was conducted with a 0.8 × volume of AMpure XP beads according to the manufacturer’s instructions, followed by elution with 10 µL nuclease-free water. The adapter sequence was added to the target transcripts in the library amplification step. The reaction mixture contained 4 µL of the RNase-treated cDNA solution, 5 µL of KAPA HiFi HotStart ReadyMix, and 1 µL of gene-specific primer mix (total 100 µM, see Additional file 3: Table S2). The reaction was conducted at 95 °C for 5 min, 98 °C for 20 s, 60 °C for 15 s, 72 °C for 40 s and then kept at 4 °C until the next reaction. Purification was conducted with a 0.8 × volume of AMpure XP beads according to the manufacturer’s instructions, followed by elution with 12 µL nuclease-free water. To optimize the PCR cycle for library amplification, qPCR was conducted with 3.5 µL of the adapter-added DNA, 5 µL of KAPA HiFi HotStart ReadyMix, 0.5 µL of EvaGreen dye (20 × in water), 0.5 µL of 10 µM 2nd-index primer (AATGATACGGCGACCACCGAGATCTACACxxxxxxxxACACTCTTTCCCTACACGA, xxxxxxxx indicates index sequences for multiplex sequencing, see Additional file 3: Table S3), and 0.5 µL of 10 µM PCR_P7 primer (CAAGCAGAAGACGGCATACGAGAT). The reaction was carried out using a LightCycler 480 II at 95 °C for 5 min, then 30 cycles of 98 °C for 20 s, 60 °C for 15 s, 72 °C for 40 s, followed by 72 °C for 3 min, and then held at 4 °C. The optimal number of PCR cycles was the cycle corresponding to the middle of the exponential phase. Then, the library was amplified with 7 µL of the adapter-added DNA, 10 µL of KAPA HiFi HotStart ReadyMix, 1 µL of 10 µM 2nd index primer (Additional file 3: Table S2), and 1 µL of 10 µM PCR_P7 primer. The reaction was carried out at 95 °C for 5 min, then the optimal number of cycles of 98 °C for 20 s, 60 °C for 15 s, 72 °C for 40 s, followed by 72 °C for 3 min, and then held at 4 °C. The amplified library was purified with an equal volume of AMpure XP beads and eluted with 15 µL of 10 mM Tris–HCl (pH 7.6). One microliter of the library was electrophoresed using a Bioanalyzer 2100 with Agilent High Sensitivity DNA kit to check for quality. Sequencing of 140-bp single-ends was carried out with a MiSeq system (Illumina, San Diego, CA, USA). The sequences of the first 50-bp of the 5′-end were used in the subsequent analysis.

An update of the DeLTa-Seq protocol is available at our website (https://sites.google.com/view/delta-seq/).

### Targeted RNA-Seq

For Fig. 5, RNA was extracted from the bulked homogenate of approximately 10 *A. thaliana* seedlings 14 days after sowing. Then, 0.01, 0.1, 1, and 10 ng of the purified RNA were used for reverse transcription with 96, 96, 10, and 5 technical replicates, respectively. The following experiments were conducted as described in the ‘Direct-lysate targeted RNA-Seq (DeLTa-Seq)’ section.

### Preparation and sequencing of 96 technical replicates of non-targeted and targeted RNA-Seq

For Fig. 6, RNA was extracted from the bulked homogenate of approximately 250 *A. thaliana* seedlings 7 days after sowing. RT reactions, pooling, and purification were conducted as described in the ‘Non-targeted RNA-Seq for Fig. 3’ section, except that 250 ng of template RNA was used for each technical replicate. The pooled cDNA was eluted with 50 μL of nuclease-free water in the purification step.

For non-targeted RNA-Seq, 20 μL of pooled cDNA was used. The following steps were conducted as described in the ‘Non-targeted RNA-Seq for Fig. 3’ section with some changes. Following RNase treatment, dsDNA was eluted with 10 µL nuclease-free water. Optimization of the PCR amplification cycle was conducted with 4 µL of the adapter-ligated dsDNA. Then, the library was amplified using 7 µL of the adapter-ligated DNA. The amplified library was purified and eluted with 15 µL of 10 mM Tris–HCl (pH 7.6).

For targeted RNA-Seq, the following experiments were conducted as described in the ‘Direct-lysate targeted RNA-Seq (DeLTa-Seq) for Fig. 4’ section.

Equal amounts of non-targeted and targeted RNA-Seq libraries were mixed based on qPCR-based quantification with Kapa Library Quantification Kit (KAPA BIOSYSTEMS). Sequencing of 150-bp paired-ends was performed using a HiSeq.

### Subsampling of RNA-Seq reads

Subsampling of RNA-Seq reads was conducted with ‘seqtk’ (https://github.com/lh3/seqtk) to normalize total reads. The seed parameter ‘-s’ was specified differently in each subsampling.

### Calculation and normalization of RNA-Seq count data

All obtained reads were processed with Trimmomatic (version 0.3.3) [55] using the following parameters: TOPHRED33 ILLUMINACLIP: TruSeq3-SE.fa:2:30:10 LEADING:19 TRAILING:19 SLIDINGWINDOW:30:20 AVGQUAL:20 MINLEN:40. This procedure removed adapter sequences (ILLUMINACLIP:TruSeq3-PE.fa:2:30:10) and leading and trailing low quality or N bases (below quality Phred score 19) (LEADING:19 TRAILING:19). In addition, the reads were trimmed when the average quality per base dropped below 20 with a 30-base wide sliding window (SLIDINGWINDOW:30:20). Finally, trimmed reads with length > 39 nucleotides and average quality score > 19 were output. The trimmed reads were then mapped to the *O. sativa* reference sequences of IRGSP-1.0_transcript [56, 57], rRNAs, and transcripts coded in the mitochondria and chloroplast genomes (GenBank: NC_001320.1 and NC_011033.1), the *A. thaliana* reference sequence of Araport11 representative transcripts [58], the *S. cerevisiae* cDNA R64-1-1 reference sequence of ensemble [59], or *D. rerio* GRCz11.cdna.all reference sequences of ensemble [59] with RSEM (version 1.3.0) [60], using Bowtie (version 1.1.2) [61] with default parameters.

When using UMI in the analysis, all obtained paired reads were processed using dynacomkobe/biodocker_rnaseq_pipeline:ver.0.2.0 with the default parameters, with quality trimming using Trimmomatic, and quantification with RSEM and Bowtie.

The expected counts of each gene in the RSEM outputs were used with R (version 3.4.2) [62] in the following analysis. The R script used in this study was deposited in https://github.com/naganolab/DeLTa-Seq.git. Briefly, read counts were normalized by calculating read per million (RPM) using the total read count of all transcripts as denominators. Differentially quantified genes (FDR = 0.05) were detected as described by Sun et al. [63].

### DeLTa-Seq analysis of *A. thaliana* seedlings treated with EtOH, jasmonic acid, and salicylic acid

A seed of *A. thaliana* was sowed in 100 µL of 0.5 × Murashige and Skoog medium in each well of a 96-well plate and grown at 20 °C under continuous light for 7 days after vernalization treatment. Each of the 11 96-well plates was incubated at a different temperature (10, 12, 14, 16, 18, 20, 22, 24, 26, 28 and 30 °C) for 18 h. Then, each 96-well plate was divided into three parts of 32 wells each, and each part was filled with 50% EtOH, or 16.5 mM of JA or SA stock solution, forming a final concentration of 1%, 0.33 mM, and 0.33 mM, respectively; the plates were incubated for an additional 6 h. After incubation, medium was immediately discarded using 96 channel pipette (epMotion 96 (Eppendorf, Hamburg, Germany)), and *A. thaliana* seedlings were homogenized twice in 90 s with 200 µL of 90 mM Tris–HCl (pH 7.6) containing 100 mM DTT using TissueLyser II (Qiagen, Hilden, Germany). DeLTa-seq libraries were created as described above with minor modifications. RT products were obtained from 5 µL of lysate as the template. Pooled RT products were divided into two aliquots and each sample was allowed to react with either of the two gene specific primer mixes, Tradict targeting genes (Additional file 3: Table S2) or SA/JA-related genes (Additional file 3: Table S4).

### Heatmap analysis of DeLTA-Seq results

Heatmap of scaled log2 RPM + 1 of target genes was illustrated using NormalizeData, ScaleData, and DoHeatamap in R package Seurat (version 4.05) [64].

### Tukey's test

Tukey's test was conducted with multcomp R package (version 1.4-19) with the default parametors [65].

## Supplementary Information

**Additional file 1: Data 1** Expressions of gene set 1 in EtOH-, jasmonic-acid-, and salicylic-acid-treated *A. thaliana* seedlings. Magenta lines represent trend curves of each target log2 RPM + 1 with the ambient temperatures.

**Additional file 2: Data 2** Expressions of gene set 2 in EtOH-, jasmonic-acid-, and salicylic-acid-treated *A. thaliana* seedlings. Magenta lines represent trend curves of each target log2 RPM + 1 with the ambient temperatures.

**Additional file 3:** **Table S1** List of the RT primers harboring 1st indexes. **Table S2** List of the gene-specific primers for gene set 1 (Tradict target gene set). **Table S3**. List of the PCR primers harboring 2nd indexes. **Table S4**. List of the gene-specific primers for gene set 2 (JA- and SA-related genes).

**Additional file 4**: **Fig. S1. **Performance of previously reported buffers for mammalian cultured cells in direct-lysate reverse transcription of *A. thaliana*, *O. sativa*, and *D. rerio*. **Fig. S2. **Comparison of RNA-Seq results of purified RNA and lysate of *S. cerevisiae *and *D. rerio*. **Fig. S3. **UMI conversion efficiencies of 96 technical replicates of non-targeted and targeted RNA-Seq.

## Data Availability

The datasets generated and analyzed during the current study are available in the SRA repository (PRJNA643885 and PRJNA813371).
