# Supplementary material for: DeLTa-Seq: direct-lysate targeted RNA-Seq from crude tissue lysate
Source: Plant Methods. 2022 Aug 6;18:99. doi: 10.1186/s13007-022-00930-x (PMC9356424; doi:10.1186/s13007-022-00930-x)
Supplement: Supplementary file 1 — Additional file 1: Data 1 Expressions of gene set 1 in EtOH-, jasmonic-acid-, and salicylic-acid-treated A. thaliana seedlings. Magenta lines represent trend curves of each target log2 RPM + 1 with the ambient temperatures. [file 13007_2022_930_MOESM1_ESM.pdf]

## AT2G26150.2

heat shock transcription factor A2

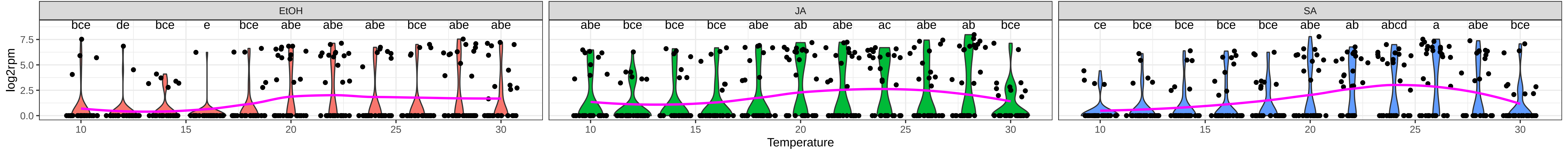

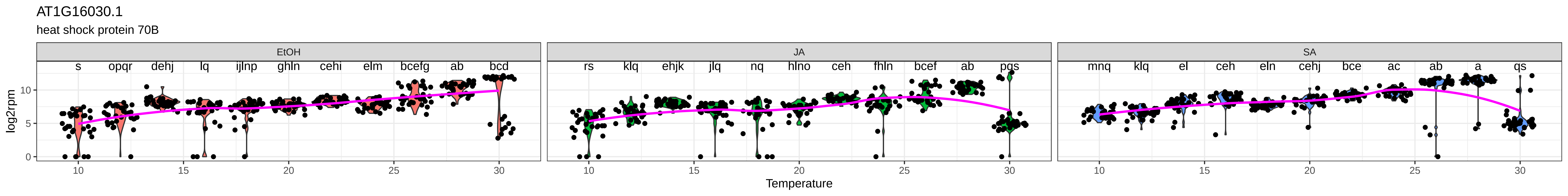

AT1G16030.1
heat shock protein 70B
EtOH
JA
SA
s
opqr
dehj
lq
ijlnp
ghln
cehi
elm
bcefg
ab
bcd
rs
klq
ehjk
jlq
nq
hlno
ceh
fhln
bcef
ab
pqs
mnq
klq
el
ceh
eln
cehj
bce
ac
ab
a
qs
log2rpm
0
5
10
10
15
20
25
30
Temperature

AT5G64040.1

photosystem I reaction center subunit PSI−N, chloroplast, putative / PSI−N, putative (PSAN)

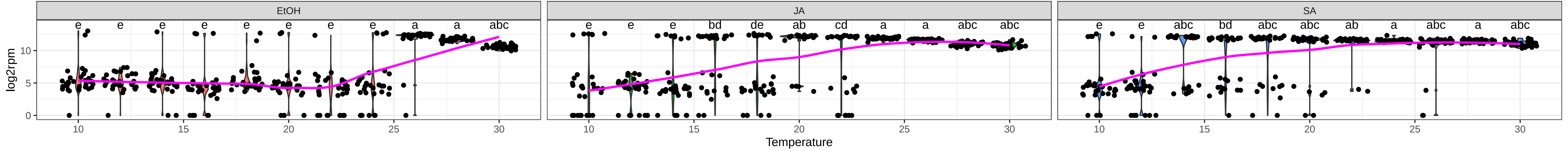

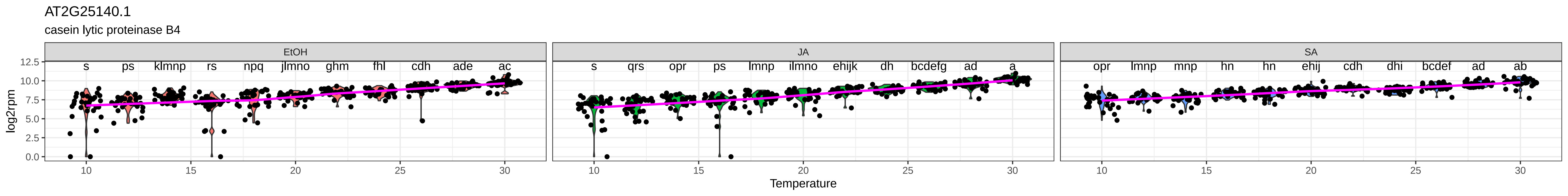

AT2G25140.1
casein lytic proteinase B4
EtOH
JA
SA
s
ps
klmnp
rs
npq
jlmno
ghm
fhl
cdh
ade
ac
s
qrs
opr
ps
lmnp
ilmno
ehijk
dh
bcdefg
ad
a
opr
lmnp
mnp
hn
hn
ehij
cdh
dhi
bcdef
ad
ab
log2rpm
12.5
10.0
7.5
5.0
2.5
0.0
10
15
20
25
30
Temperature

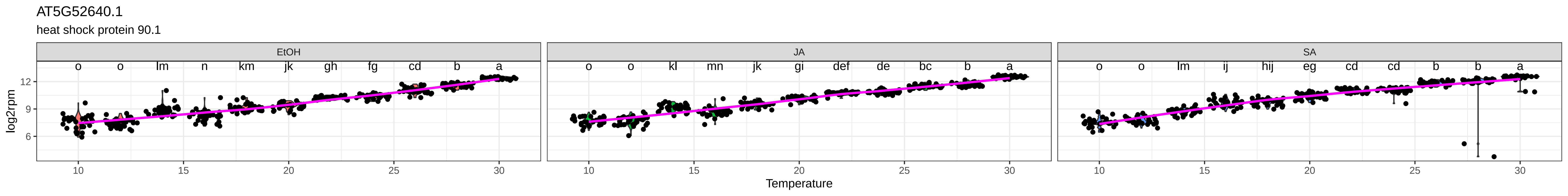

AT5G52640.1
heat shock protein 90.1
EtOH
JA
SA
o o lm n km jk gh fg cd b a
o o kl mn jk gi def de bc b a
o o lm ij hij eg cd cd b b a
log2rpm
12
9
6
10
15
20
25
30
Temperature

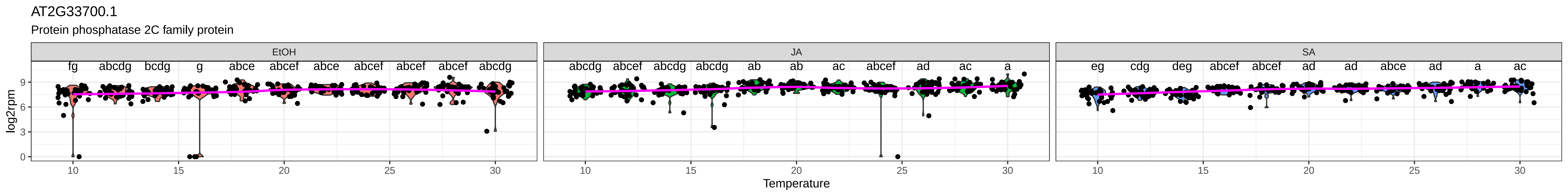
AT2G33700.1
Protein phosphatase 2C family protein
EtOH
JA
SA
fg
abcdg
bcdg
g
abce
abcef
abce
abcef
abcef
abcef
abcdg
abcdg
abcef
abcdg
abcdg
ab
ab
ac
abcef
ad
ac
a
eg
cdg
deg
abcef
abcef
ad
ad
abce
ad
a
ac
log2rpm
9
6
3
0
10
15
20
25
30
Temperature

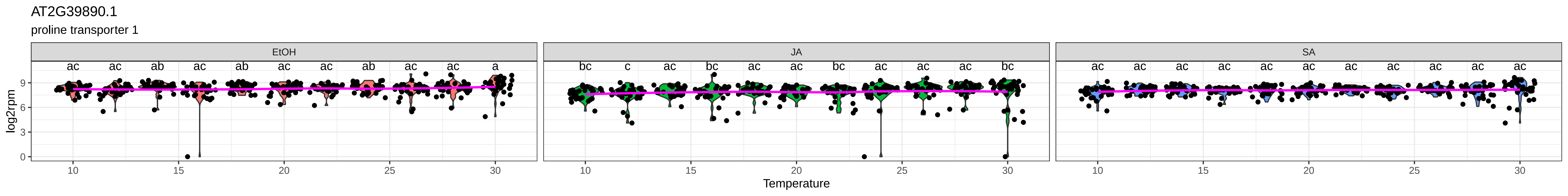

AT2G39890.1
proline transporter 1
EtOH
JA
SA
ac ac ab ac ab ac ac ab ac ac a
bc c ac bc ac ac bc ac ac ac bc
ac ac ac ac ac ac ac ac ac ac ac
log2rpm
0
3
6
9
10
15
20
25
30
Temperature

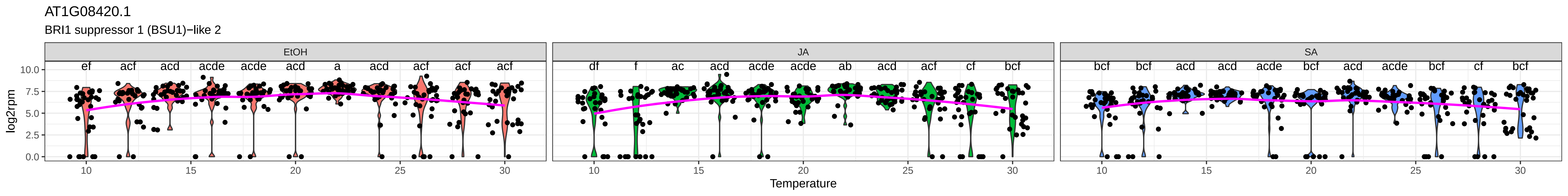

AT1G08420.1
BRI1 suppressor 1 (BSU1)−like 2
EtOH
JA
SA
ef acf acd acde acde acd a acd acf acf acf
df f ac acd acde acde ab acd acf cf bcf
bcf bcf acd acd acde bcf acd acde bcf cf bcf
log2rpm
0.0
2.5
5.0
7.5
10.0
10 15 20 25 30
Temperature

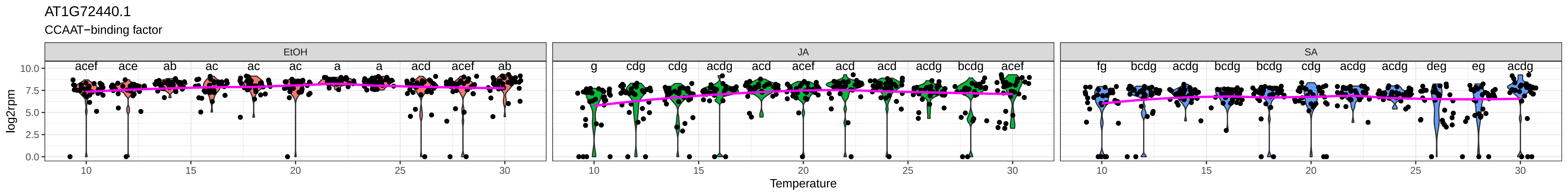

AT1G72440.1
CCAAT−binding factor
EtOH
JA
SA
acef ace ab ac ac ac a a acd acef ab
g cdg cdg acdg acd acef acd acd acdg bcdg acef
fg bcdg acdg bcdg bcdg cdg acdg acdg deg eg acdg
log2rpm
10.0
7.5
5.0
2.5
0.0
10 15 20 25 30
Temperature

# AT5G65687.1

Major facilitator superfamily protein

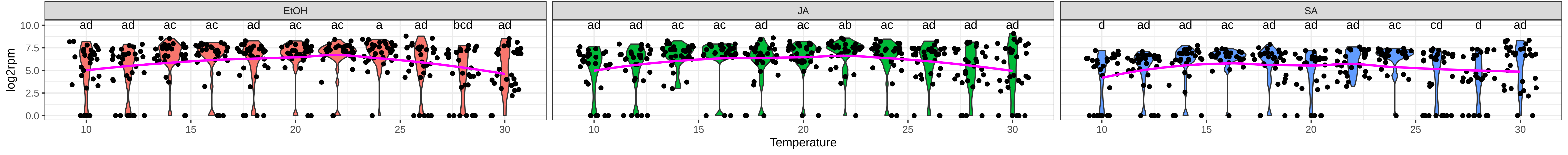

AT1G30490.1

Homeobox−leucine zipper family protein / lipid−binding START domain−containing protein

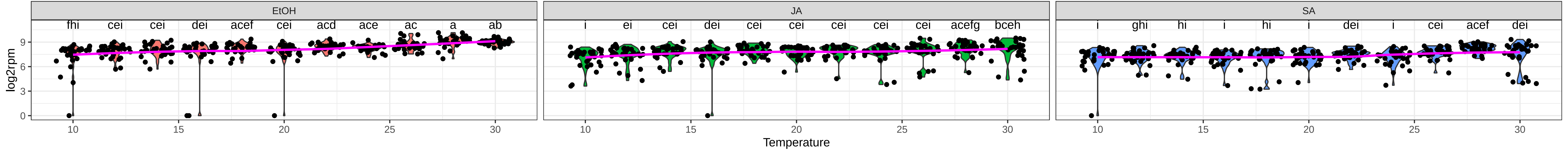

AT5G19330.1

ARM repeat protein interacting with ABF2

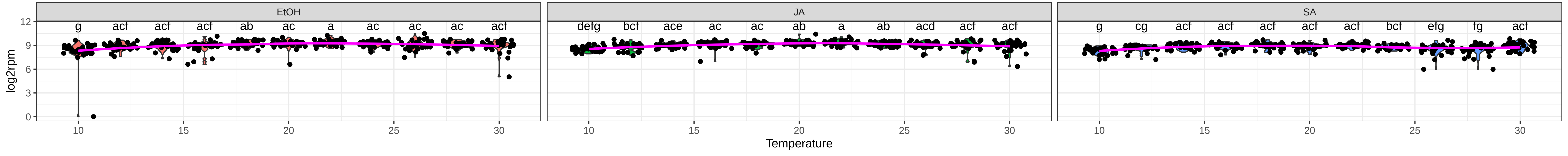

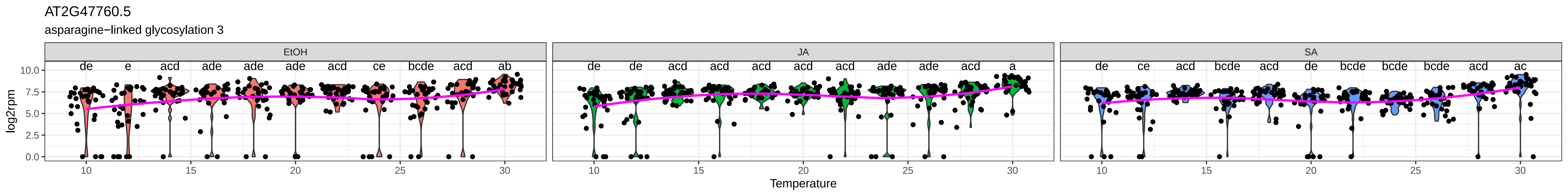

AT2G47760.5
asparagine−linked glycosylation 3
EtOH
JA
SA
de e acd ade ade ade acd ce bcde acd ab
de de acd acd acd acd acd ade ade acd a
de ce acd bcde acd de bcde bcde bcde acd ac
log2rpm
10.0
7.5
5.0
2.5
0.0
10 15 20 25 30
Temperature

AT5G19350.1

RNA−binding (RRM/RBD/RNP motifs) family protein

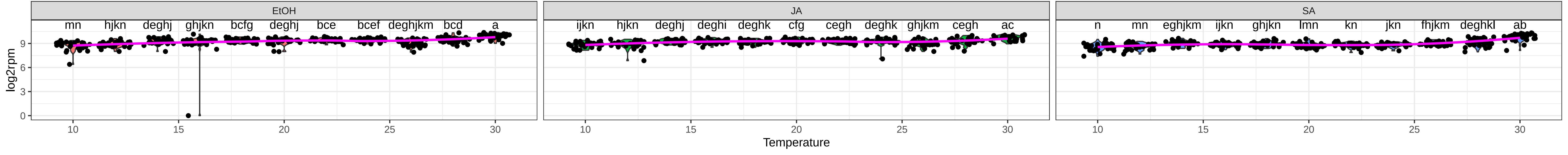

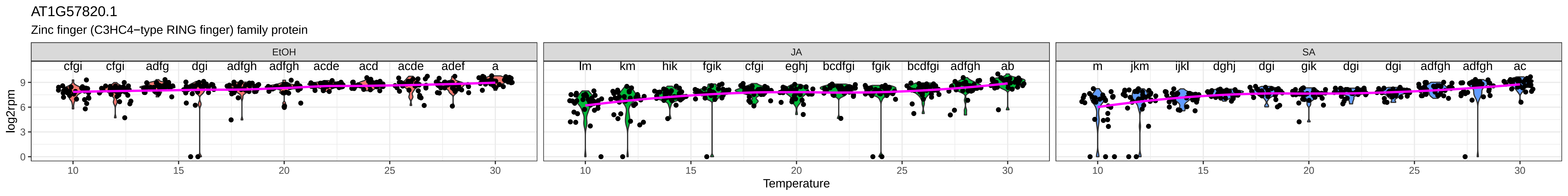
AT1G57820.1
Zinc finger (C3HC4−type RING finger) family protein
EtOH
JA
SA
cfgi cfgi adfg dgi adfgh adfgh acde acd acde adef a
lm km hik fgik cfgi eghj bcdfgi fgik bcdfgi adfgh ab
m jkm ijkl dghj dgi gik dgi dgi adfgh adfgh ac
log2rpm
9
6
3
0
10 15 20 25 30
Temperature

AT5G16620.1

hydroxyproline−rich glycoprotein family protein

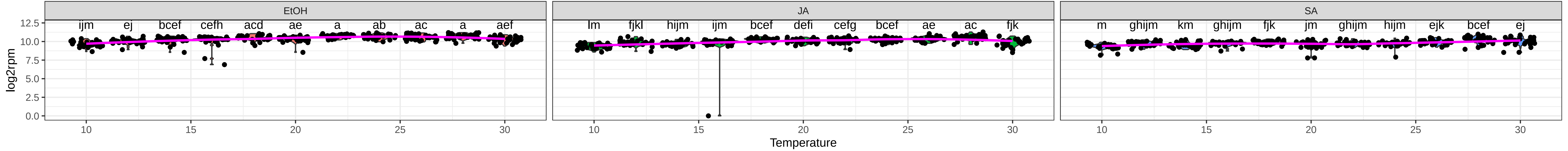

AT3G53120.1

Modifier of rudimentary (Mod(r)) protein

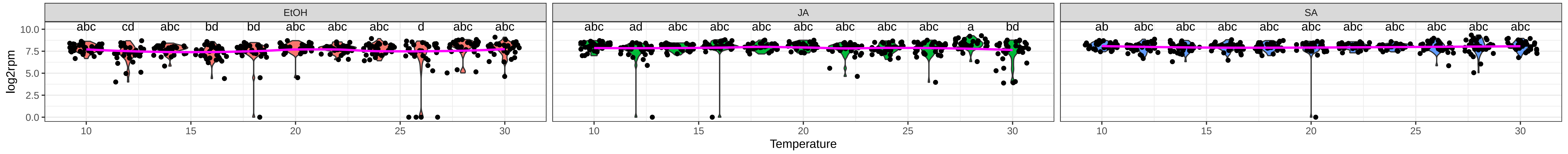

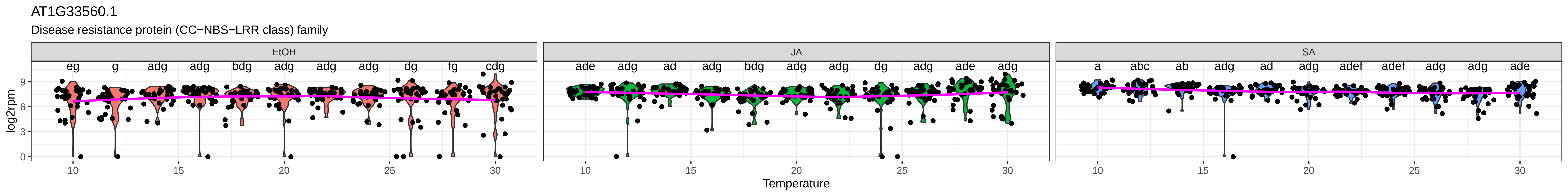

AT1G33560.1
Disease resistance protein (CC−NBS−LRR class) family
EtOH
JA
SA
eg g adg adg bdg adg adg adg dg fg cdg
ade adg ad adg bdg adg adg dg adg ade adg
a abc ab adg ad adg adef adef adg adg ade
log2rpm
Temperature

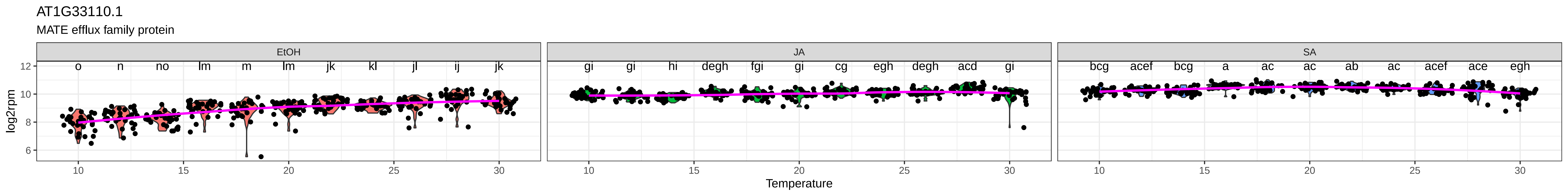

AT1G33110.1
MATE efflux family protein
EtOH
JA
SA
o n no lm m lm jk kl jl ij jk
gi gi hi degh fgi gi cg egh degh acd gi
bcg acef bcg a ac ac ab ac acef ace egh
log2rpm
12
10
8
6
10 15 20 25 30
Temperature

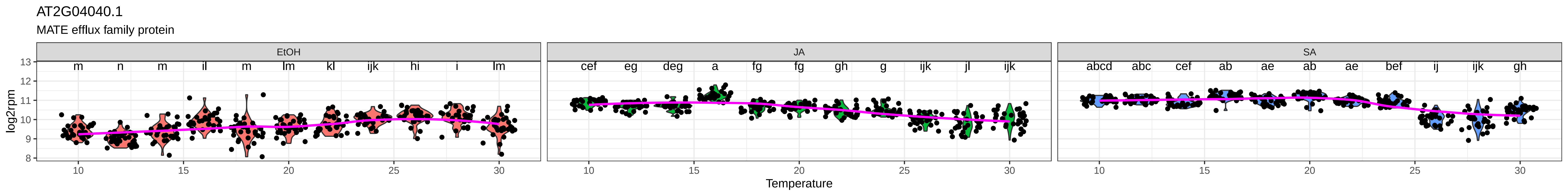
AT2G04040.1
MATE efflux family protein
EtOH
JA
SA
m
n
m
il
m
lm
kl
ijk
hi
i
lm
cef
eg
deg
a
fg
fg
gh
g
ijk
jl
ijk
abcd
abc
cef
ab
ae
ab
ae
bef
ij
ijk
gh
log2rpm
13
12
11
10
9
8
10
15
20
25
30
Temperature

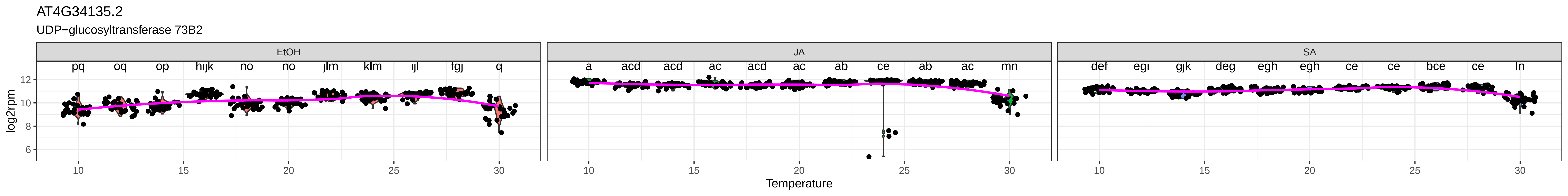
AT4G34135.2
UDP−glucosyltransferase 73B2
EtOH
JA
SA
pq oq op hijk no no jlm klm ijl fgj q
a acd acd ac acd ac ab ce ab ac mn
def egi gjk deg egh egh ce ce bce ce ln
log2rpm
12
10
8
6
10 15 20 25 30
Temperature

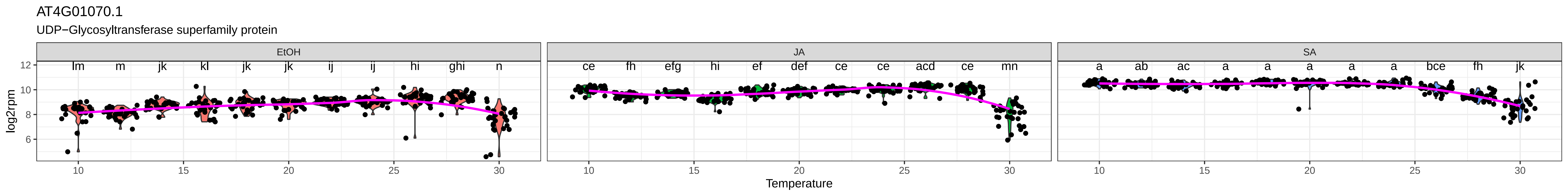
AT4G01070.1
UDP−Glycosyltransferase superfamily protein
EtOH
JA
SA
lm m jk kl jk jk ij ij hi ghi n
ce fh efg hi ef def ce ce acd ce mn
a ab ac a a a a a bce fh jk
log2rpm
12
10
8
6
10
15
20
25
30
Temperature

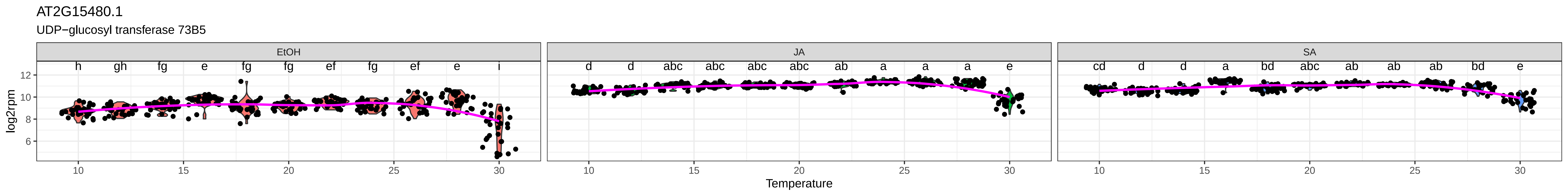
AT2G15480.1
UDP−glucosyl transferase 73B5
EtOH
JA
SA
h gh fg e fg fg ef fg ef e i
d d abc abc abc abc ab a a a e
cd d d a bd abc ab ab ab bd e
log2rpm
12
10
8
6
10 15 20 25 30
Temperature

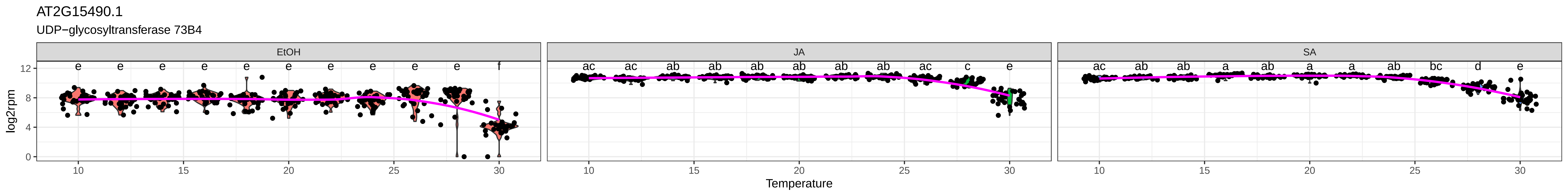
AT2G15490.1
UDP−glycosyltransferase 73B4
EtOH
JA
SA
e e e e e e e e e e f
ac ac ab ab ab ab ab ab ac c e
ac ab ab a ab a a ab bc d e
log2rpm
0
4
8
12
10
15
20
25
30
Temperature

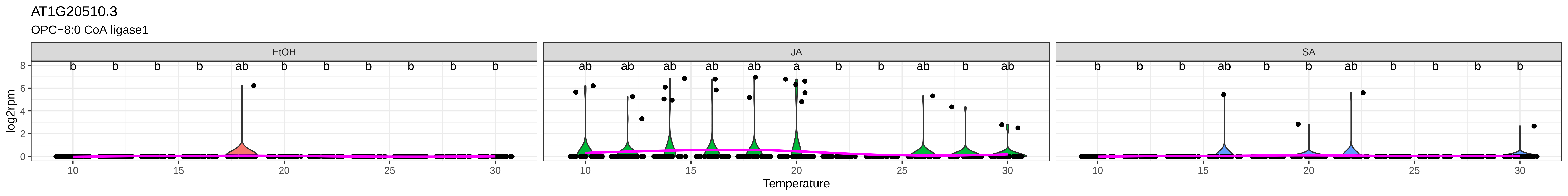

AT1G20510.3
OPC−8:0 CoA ligase1
EtOH
JA
SA
b b b b ab b b b b b b
ab ab ab ab ab a b b ab b ab
b b b ab b b ab b b b b
log2rpm
0
2
4
6
8
10
15
20
25
30
Temperature

AT4G15750.1

Plant invertase/pectin methylesterase inhibitor superfamily protein

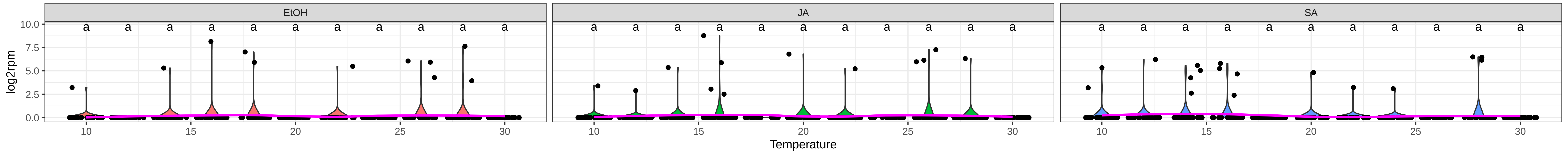

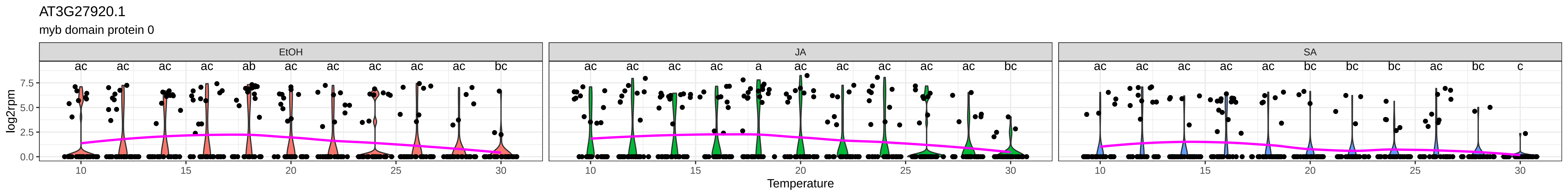

AT3G27920.1
myb domain protein 0
EtOH
JA
SA
ac ac ac ac ab ac ac ac ac ac bc
ac ac ac ac a ac ac ac ac ac bc
ac ac ac ac ac bc bc bc ac bc c
log2rpm
0.0
2.5
5.0
7.5
10 15 20 25 30
Temperature

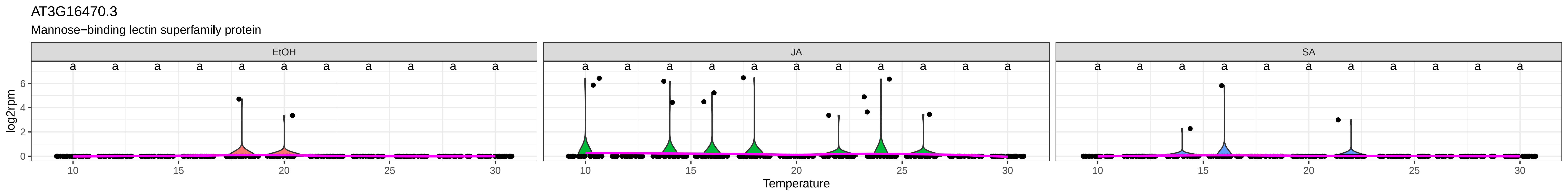

AT3G16470.3
Mannose−binding lectin superfamily protein
EtOH
JA
SA
log2rpm
Temperature
0
2
4
6
10
15
20
25
30
a

AT5G50040.1

Plant invertase/pectin methylesterase inhibitor superfamily protein

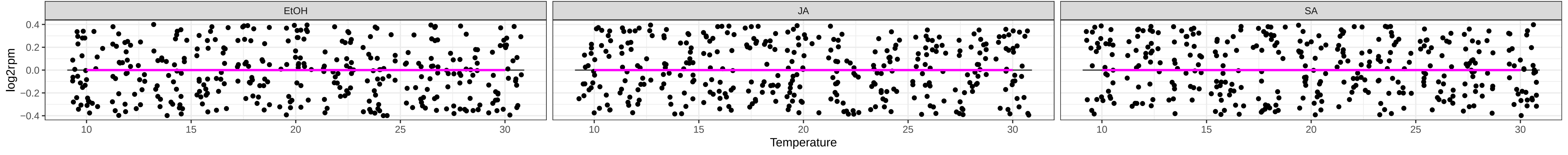

AT5G46950.1

Plant invertase/pectin methylesterase inhibitor superfamily protein

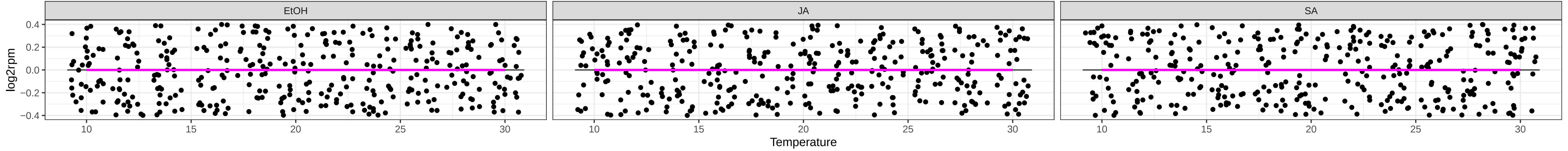

AT2G29045.1

low−molecular−weight cysteine−rich 62

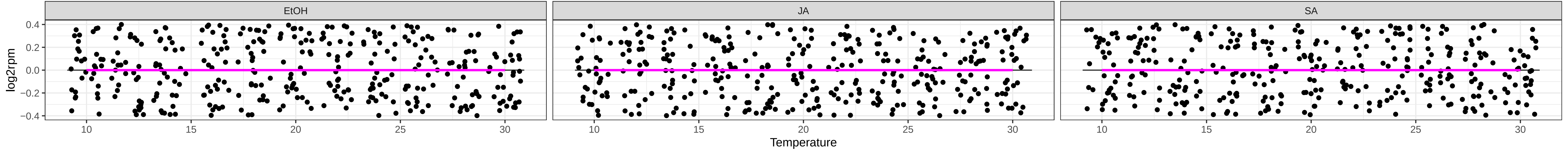

AT2G20208.1

low−molecular−weight cysteine−rich 60

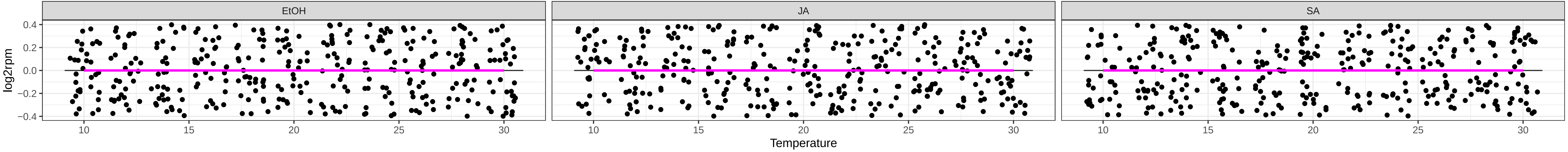

AT2G12475.1

Defensin−like (DEFL) family protein

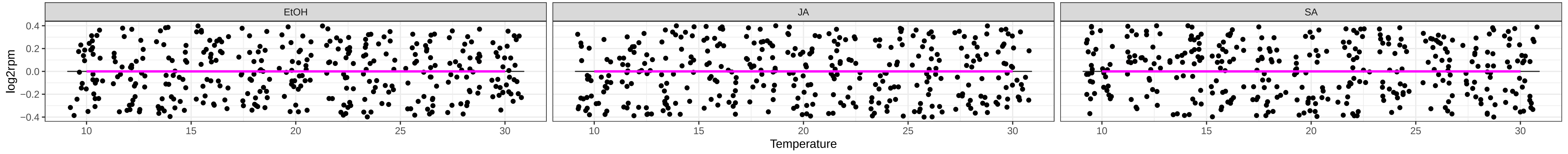

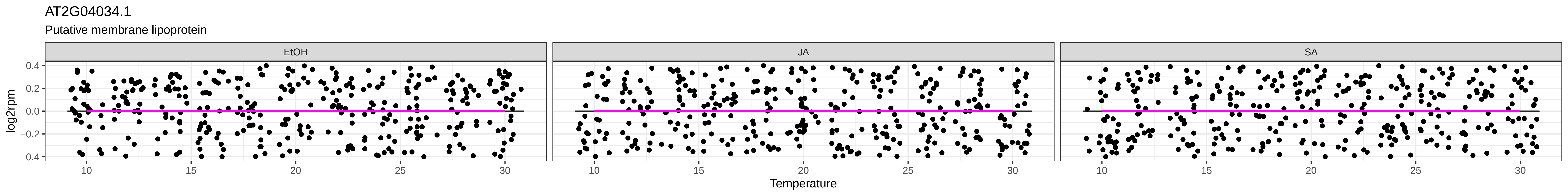

AT2G04034.1
Putative membrane lipoprotein
EtOH
JA
SA
log2rpm
0.4
0.2
0.0
-0.2
-0.4
10
15
20
25
30
Temperature

AT1G23350.1

Plant invertase/pectin methylesterase inhibitor superfamily protein

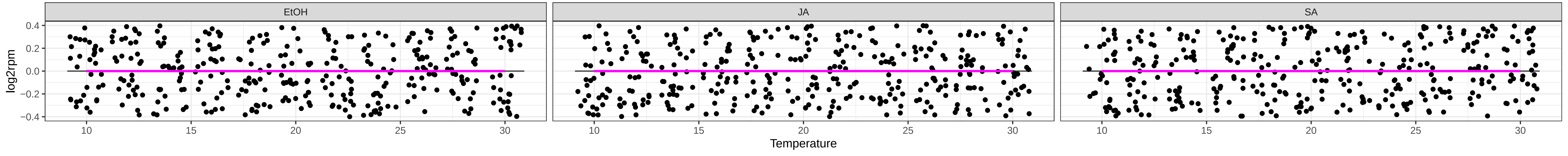

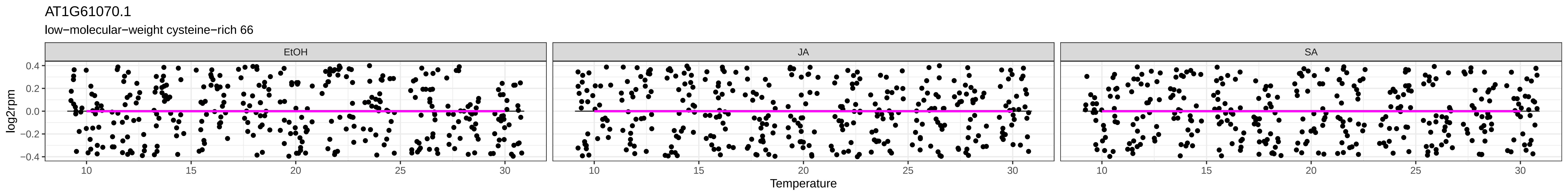

AT1G61070.1
low−molecular−weight cysteine−rich 66
EtOH
JA
SA
log2rpm
0.4
0.2
0.0
−0.2
−0.4
10
15
20
25
30
Temperature

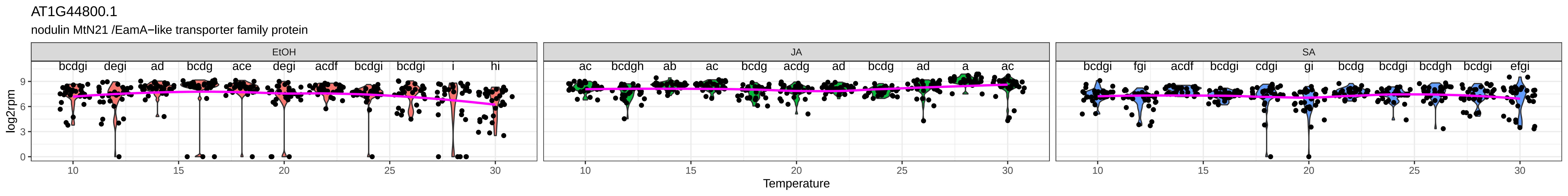
AT1G44800.1
nodulin MtN21 /EamA−like transporter family protein
EtOH
JA
SA
bcdgi degi ad bcdg ace degi acdf bcdgi bcdgi i hi
ac bcdgh ab ac bcdg acdg ad bcdg ad a ac
bcdgi fgi acdf bcdgi cdgi gi bcdg bcdgi bcdgh bcdgi efgi
log2rpm
0
3
6
9
10
15
20
25
30
Temperature

AT2G46680.1

homeobox 7

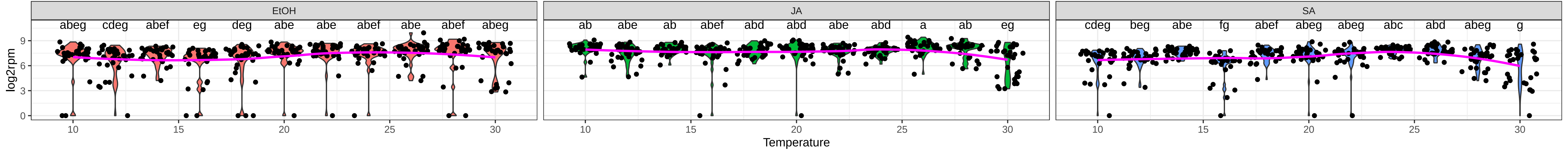

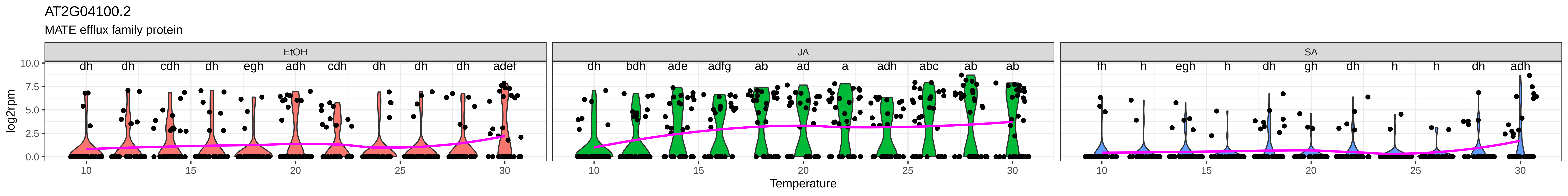
AT2G04100.2
MATE efflux family protein
EtOH
JA
SA
dh
dh
cdh
dh
egh
adh
cdh
dh
dh
dh
adef
dh
bdh
ade
adfg
ab
ad
a
adh
abc
ab
ab
fh
h
egh
h
dh
gh
dh
h
h
dh
adh
log2rpm
0.0
2.5
5.0
7.5
10.0
10
15
20
25
30
Temperature

AT4G29040.1

regulatory particle AAA−ATPase 2A

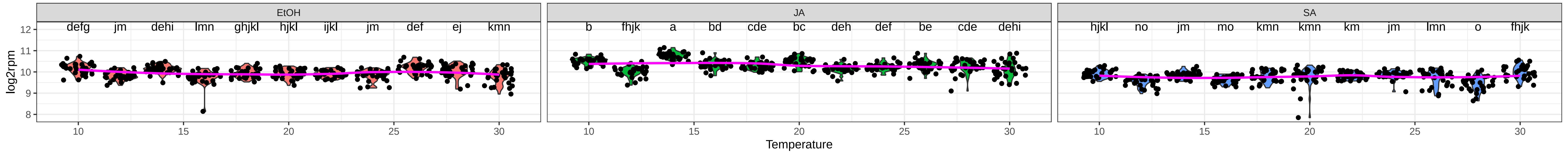

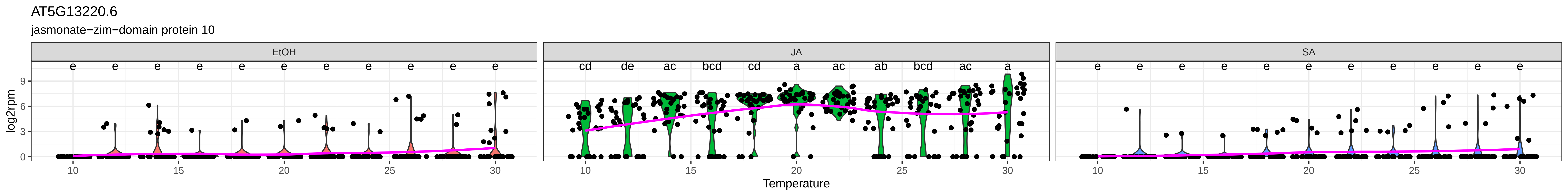
AT5G13220.6
jasmonate−zim−domain protein 10
EtOH
JA
SA
log2rpm
Temperature
e e e e e e e e e e e
cd de ac bcd cd a ac ab bcd ac a
e e e e e e e e e e e
0
3
6
9
10
15
20
25
30

AT1G55320.1

acyl−activating enzyme 18

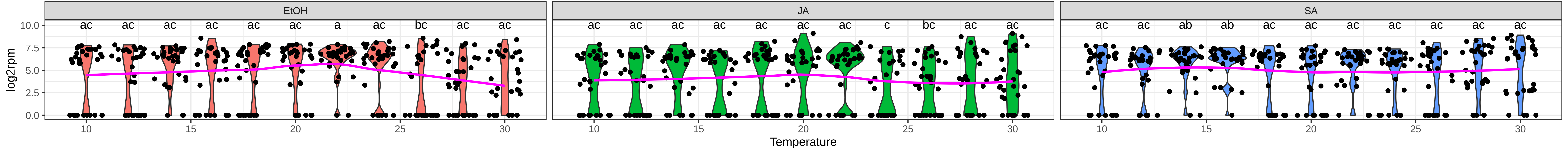

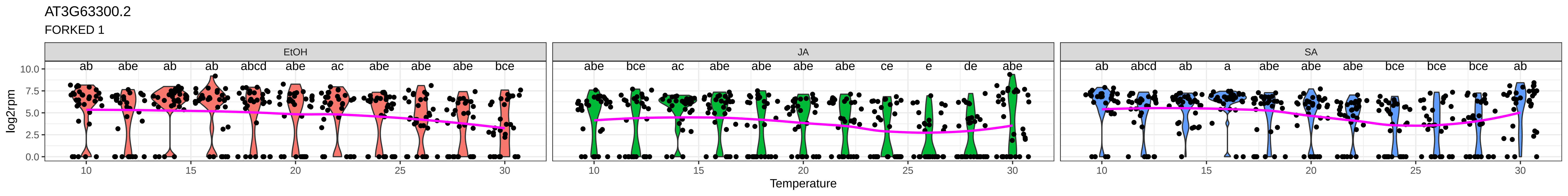
AT3G63300.2
FORKED 1
EtOH
JA
SA
ab abe ab ab abcd abe ac abe abe abe bce
abe bce ac abe abe abe abe ce e de abe
ab abcd ab a abe abe abe bce bce bce ab
log2rpm
0.0
2.5
5.0
7.5
10.0
10
15
20
25
30
Temperature

AT3G04400.1

Ribosomal protein L14p/L23e family protein

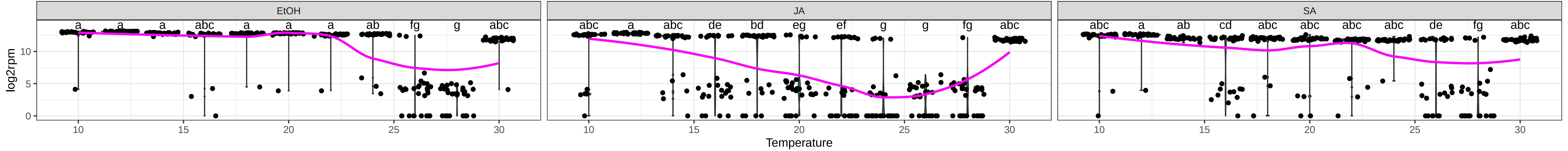

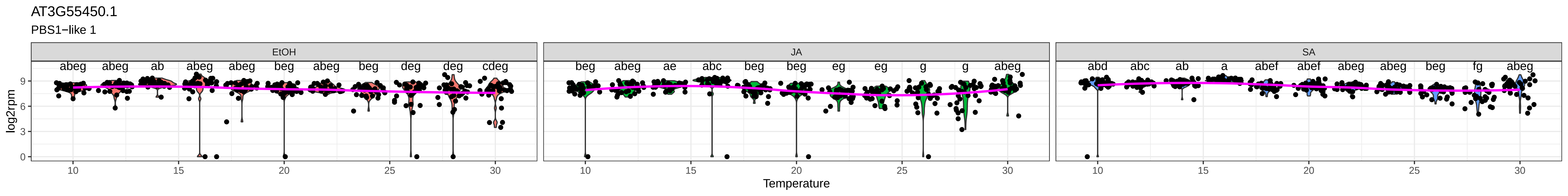
AT3G55450.1
PBS1−like 1
EtOH
JA
SA
abeg abeg ab abeg abeg beg abeg beg deg deg cdeg
beg abeg ae abc beg beg eg eg g g abeg
abd abc ab a abef abef abeg abeg beg fg abeg
log2rpm
0
3
6
9
10
15
20
25
30
Temperature

AT4G38250.1

Transmembrane amino acid transporter family protein

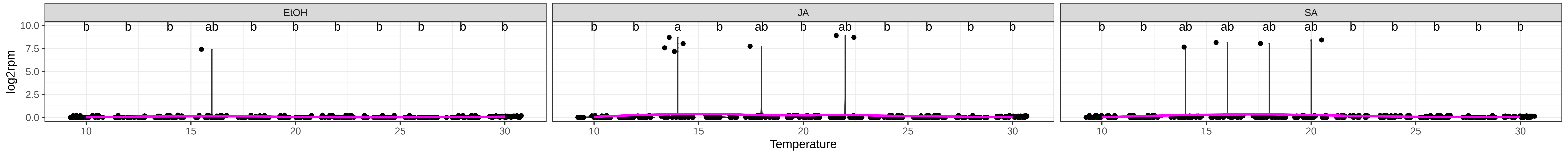

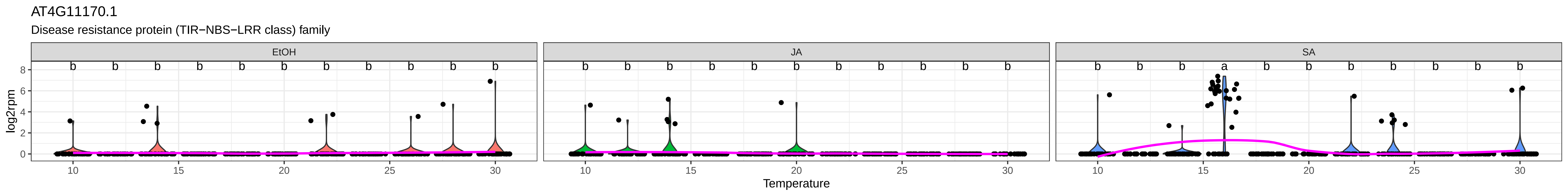
AT4G11170.1
Disease resistance protein (TIR−NBS−LRR class) family
EtOH
JA
SA
log2rpm
Temperature
b b b b b b b b b b b
b b b b b b b b b b b
b b b a b b b b b b b
0
2
4
6
8
10
15
20
25
30

AT5G08550.1

GC−rich sequence DNA−binding factor−like protein

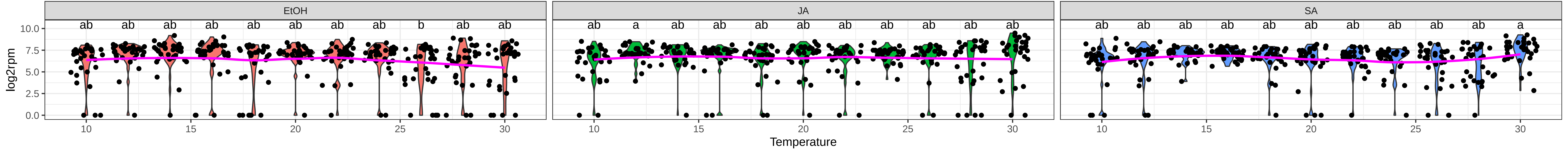

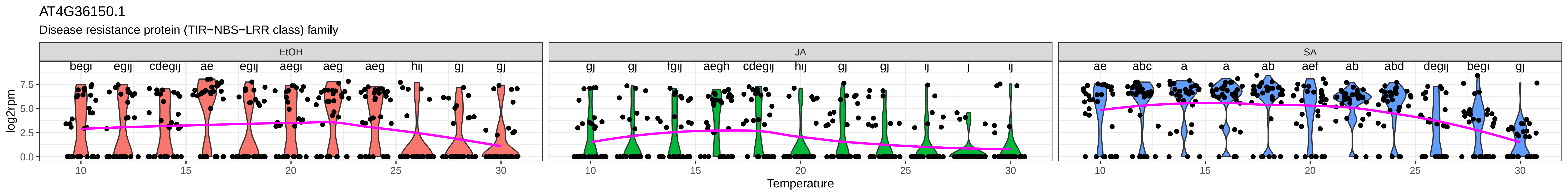

AT4G36150.1
Disease resistance protein (TIR−NBS−LRR class) family
EtOH
JA
SA
begi egij cdegij ae egij aegi aeg aeg hij gj gj
gj gj fgij aegh cdegij hij gj gj ij j ij
ae abc a a ab aef ab abd degij begi gj
log2rpm
0.0
2.5
5.0
7.5
10 15 20 25 30
Temperature

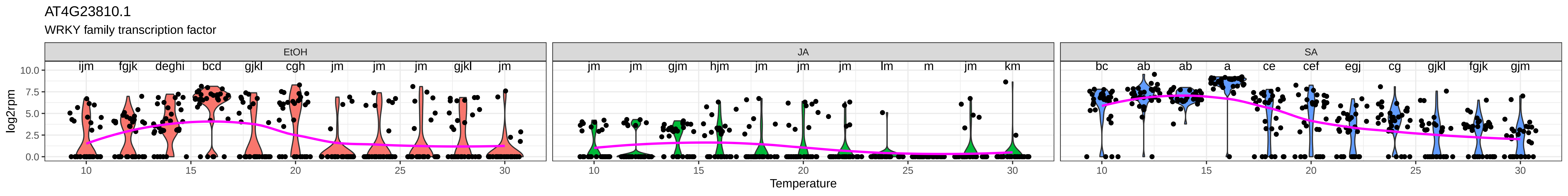

AT4G23810.1
WRKY family transcription factor
EtOH
JA
SA
ijm fgjk deghi bcd gjkl cgh jm jm jm gjkl jm
jm jm gjm hjm jm jm jm lm m jm km
bc ab ab a ce cef egj cg gjkl fgjk gjm
log2rpm
10.0
7.5
5.0
2.5
0.0
10 15 20 25 30
Temperature

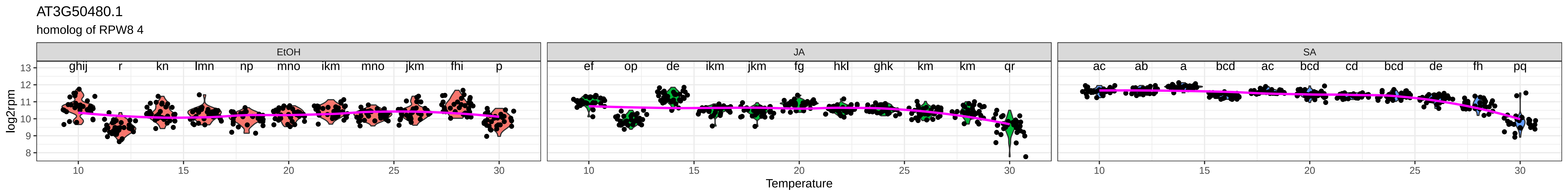

AT3G50480.1
homolog of RPW8 4
EtOH
JA
SA
ghij r kn lmn np mno ikm mno jkm fhi p
ef op de ikm jkm fg hkl ghk km km qr
ac ab a bcd ac bcd cd bcd de fh pq
log2rpm
Temperature

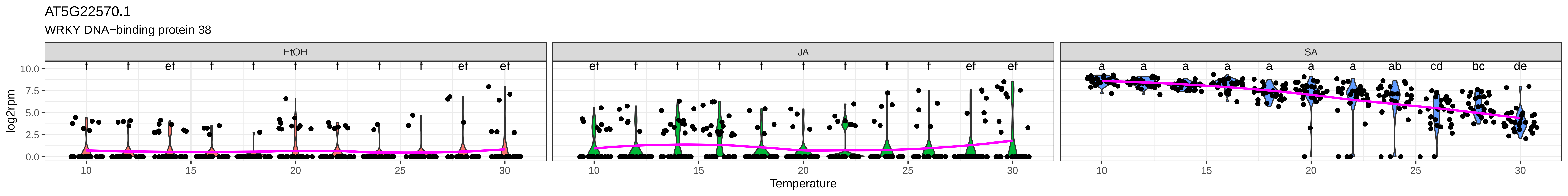
AT5G22570.1
WRKY DNA−binding protein 38
EtOH
JA
SA
f f ef f f f f f f ef ef
ef f f f f f f f f ef ef
a a a a a a a ab cd bc de
log2rpm
0.0
2.5
5.0
7.5
10.0
10
15
20
25
30
Temperature

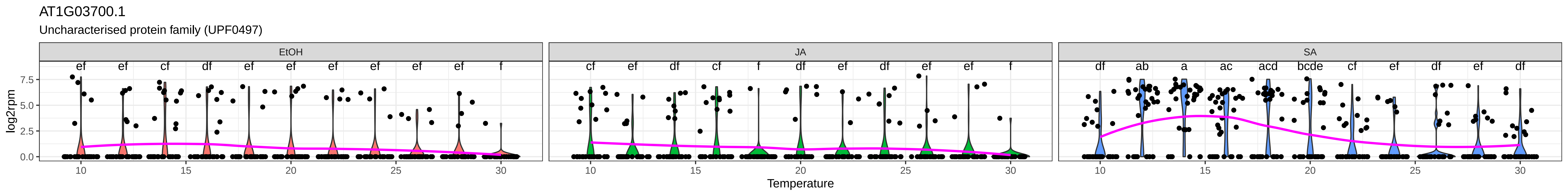
AT1G03700.1
Uncharacterised protein family (UPF0497)
EtOH
JA
SA
ef ef cf df ef ef ef ef ef ef f
cf ef df cf f df ef df ef ef f
df ab a ac acd bcde cf ef df ef df
log2rpm
0.0
2.5
5.0
7.5
10
15
20
25
30
Temperature

AT3G17150.1

Plant invertase/pectin methylesterase inhibitor superfamily protein

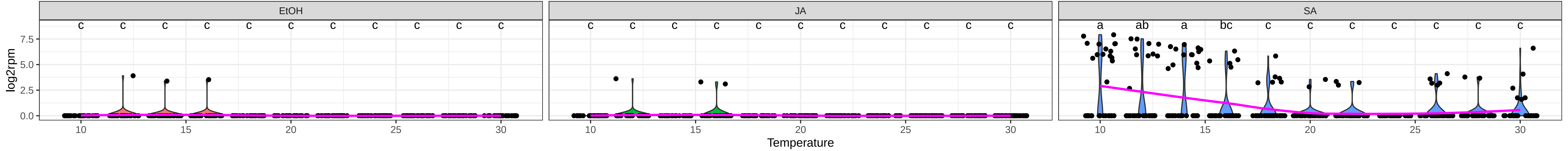

AT5G50030.1

Plant invertase/pectin methylesterase inhibitor superfamily protein

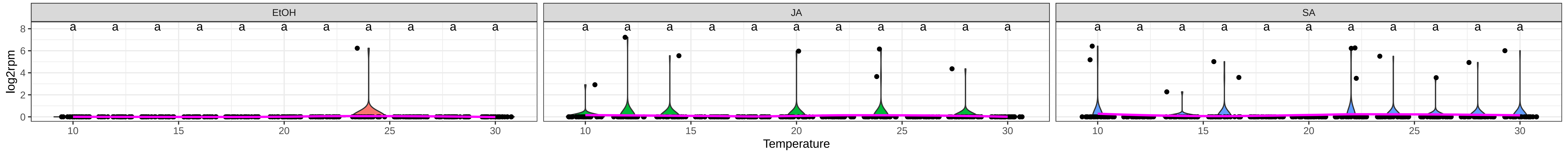

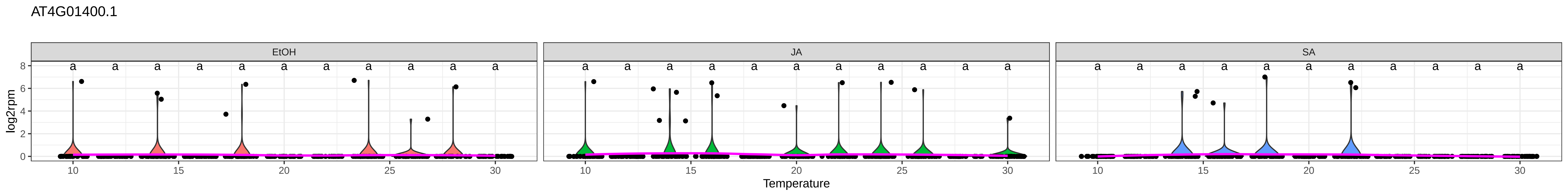

AT4G01400.1
EtOH
JA
SA
a
log2rpm
Temperature

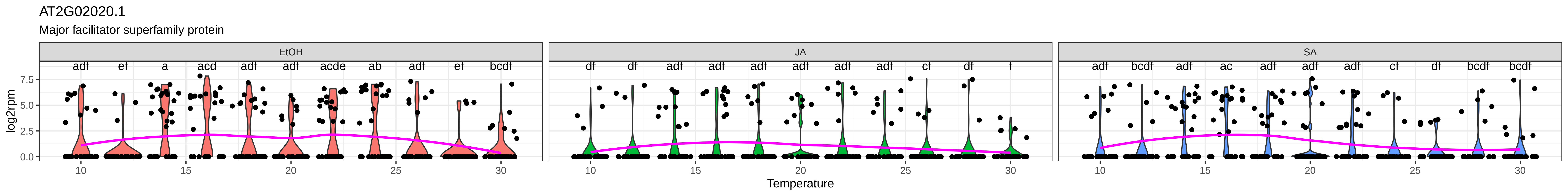
AT2G02020.1
Major facilitator superfamily protein
EtOH
JA
SA
adf
ef
a
acd
adf
adf
acde
ab
adf
ef
bcdf
df
df
adf
adf
adf
adf
adf
adf
cf
df
f
adf
bcdf
adf
ac
adf
adf
adf
cf
df
bcdf
bcdf
log2rpm
7.5
5.0
2.5
0.0
10
15
20
25
30
Temperature

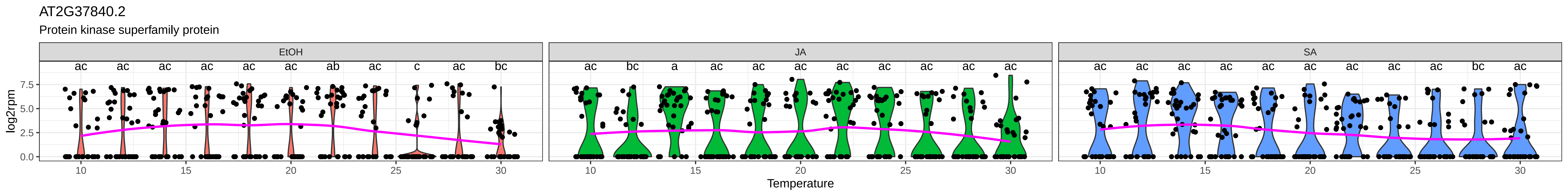
AT2G37840.2
Protein kinase superfamily protein
EtOH
JA
SA
ac ac ac ac ac ac ab ac c ac bc
ac bc a ac ac ac ac ac ac ac ac
ac ac ac ac ac ac ac ac ac bc ac
log2rpm
0.0
2.5
5.0
7.5
10
15
20
25
30
Temperature

AT1G15580.1

indole−3−acetic acid inducible 5

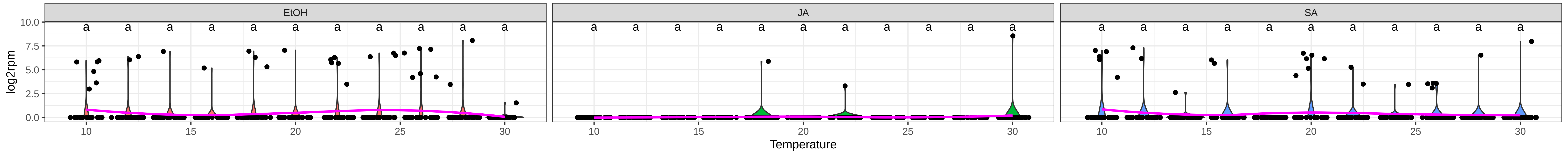

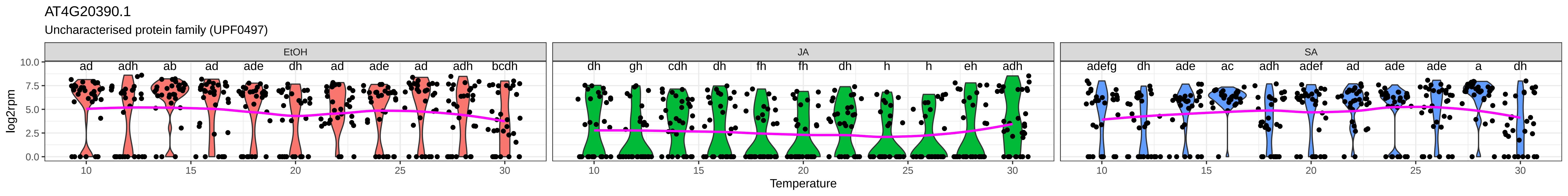

AT4G20390.1
Uncharacterised protein family (UPF0497)
EtOH
JA
SA
ad
adh
ab
ad
ade
dh
ad
ade
ad
adh
bcdh
dh
gh
cdh
dh
fh
fh
dh
h
h
eh
adh
adefg
dh
ade
ac
adh
adef
ad
ade
ade
a
dh
log2rpm
10.0
7.5
5.0
2.5
0.0
10
15
20
25
30
Temperature

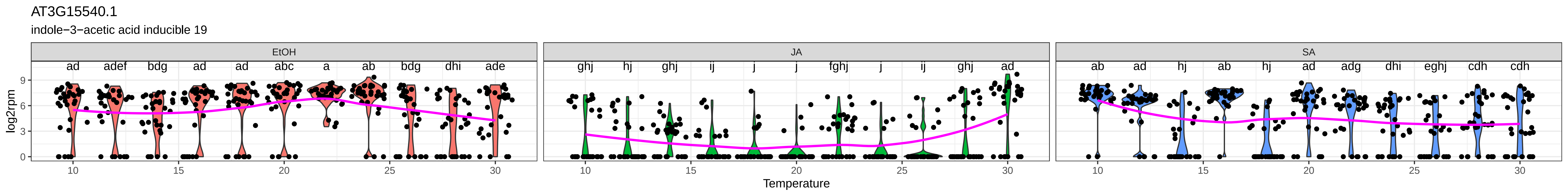
AT3G15540.1
indole−3−acetic acid inducible 19
EtOH
JA
SA
ad adef bdg ad ad abc a ab bdg dhi ade
ghj hj ghj ij j j fghj j ij ghj ad
ab ad hj ab hj ad adg dhi eghj cdh cdh
log2rpm
0
3
6
9
10
15
20
25
30
Temperature

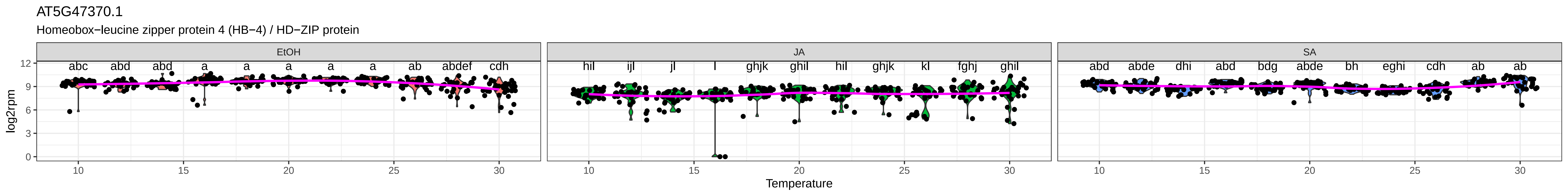
AT5G47370.1
Homeobox−leucine zipper protein 4 (HB−4) / HD−ZIP protein
EtOH
JA
SA
abc
abd
abd
a
a
a
a
a
ab
abdef
cdh
hil
ijl
jl
l
ghjk
ghil
hil
ghjk
kl
fghj
ghil
abd
abde
dhi
abd
bdg
abde
bh
eghi
cdh
ab
ab
log2rpm
12
9
6
3
0
10
15
20
25
30
Temperature

AT1G29500.1

SAUR−like auxin−responsive protein family

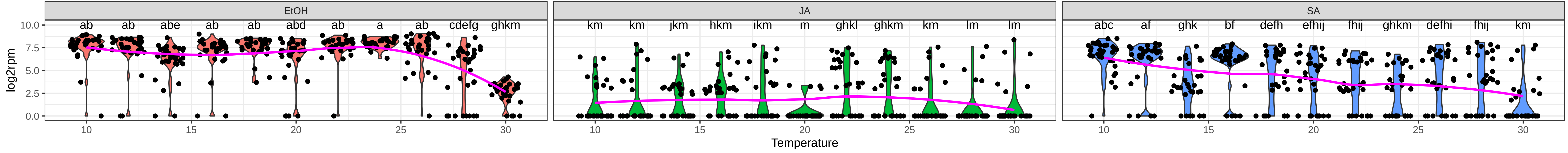

AT3G10520.1

haemoglobin 2

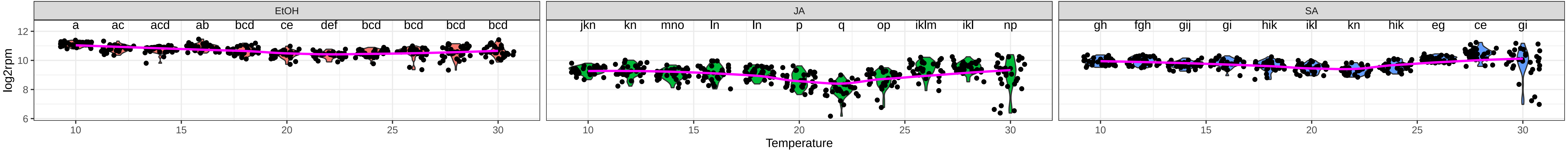

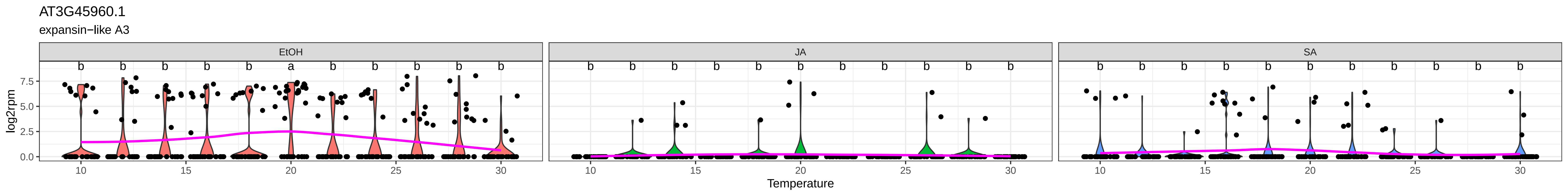

AT3G45960.1
expansin−like A3
EtOH
JA
SA
b b b b b a b b b b b
b b b b b b b b b b b
b b b b b b b b b b b
log2rpm
0.0
2.5
5.0
7.5
10
15
20
25
30
Temperature

AT2G14900.1

Gibberellin−regulated family protein

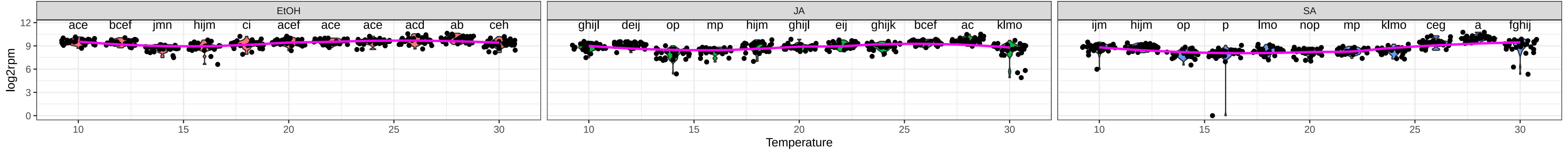

AT3G18760.1

Translation elongation  factor EF1B/ribosomal protein S6 family protein

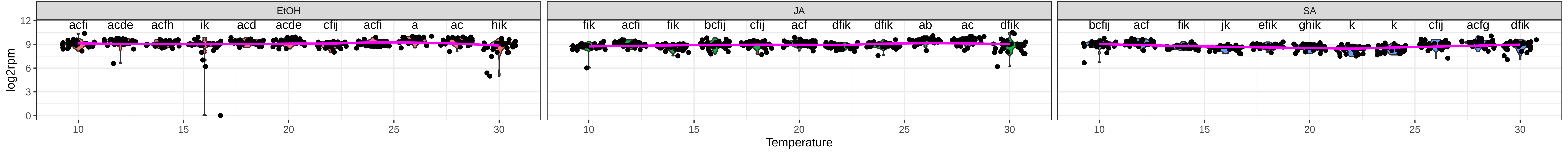

AT1G79960.1

ovate family protein 14

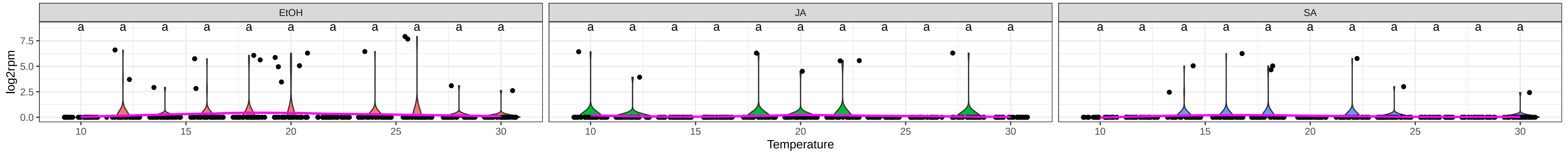

AT2G47050.1

Plant invertase/pectin methylesterase inhibitor superfamily protein

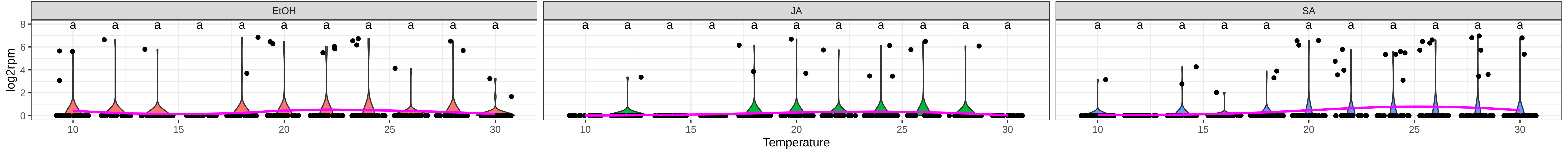

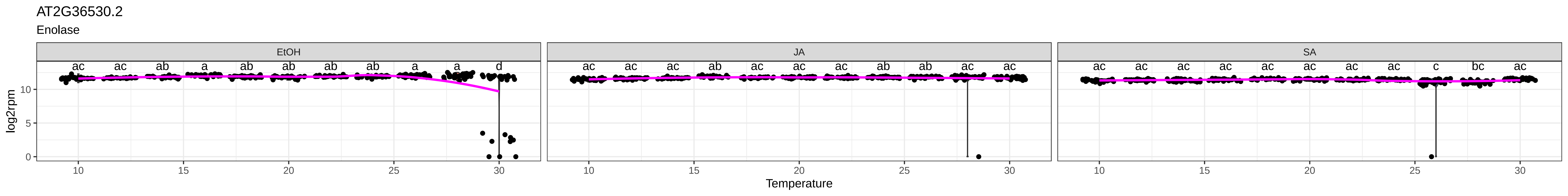

AT2G36530.2
Enolase
EtOH
JA
SA
ac ac ab a ab ab ab ab a a d
ac ac ac ab ac ac ac ab ab ac ac
ac ac ac ac ac ac ac ac c bc ac
log2rpm
0
5
10
10
15
20
25
30
Temperature

AT5G26230.1

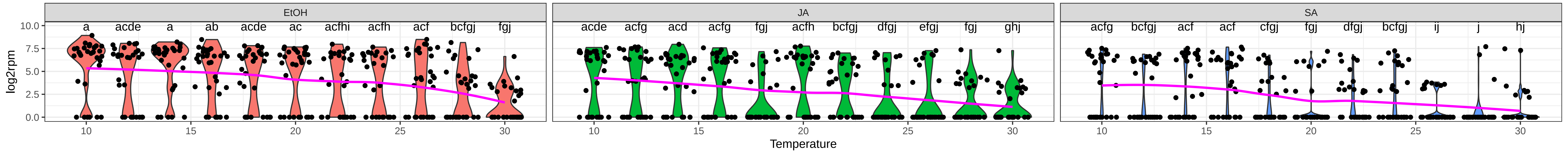
EtOH
JA
SA
a
acde
a
ab
acde
ac
acfhi
acfh
acf
bcfgj
fgj
acde
acfg
acd
acfg
fgj
acfh
bcfgj
dfgj
efgj
fgj
ghj
acfg
bcfgj
acf
acf
cfgj
fgj
dfgj
bcfgj
ij
j
hj
log2rpm
10.0
7.5
5.0
2.5
0.0
10
15
20
25
30
Temperature

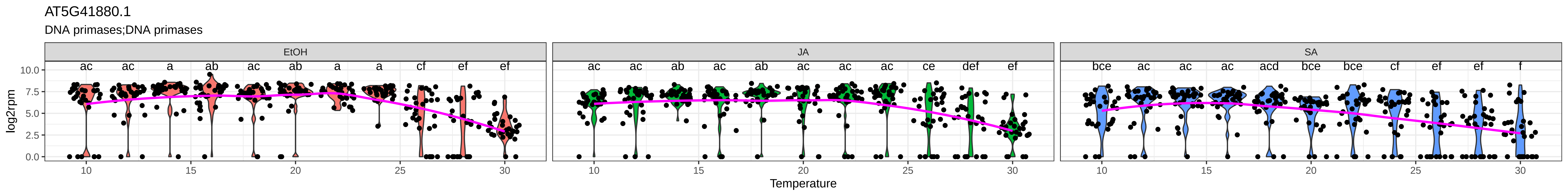

AT5G41880.1
DNA primases;DNA primases
EtOH
JA
SA
ac ac a ab ac ab a a cf ef ef
ac ac ab ac ab ac ac ac ce def ef
bce ac ac ac acd bce bce cf ef ef f
log2rpm
0.0
2.5
5.0
7.5
10.0
10 15 20 25 30
Temperature

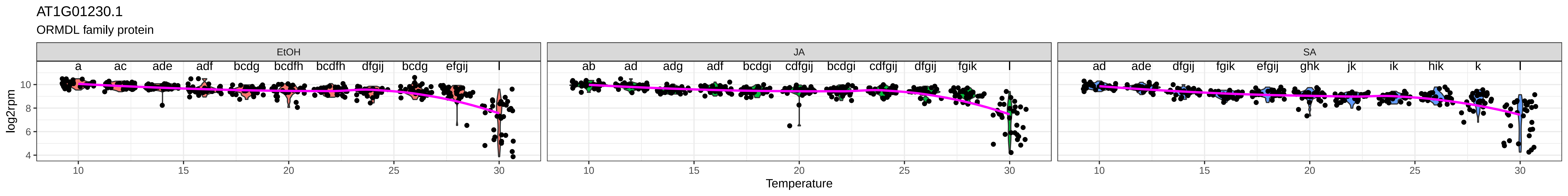
AT1G01230.1
ORMDL family protein
EtOH
JA
SA
a
ac
ade
adf
bcdg
bcdfh
bcdfh
dfgij
bcdg
efgij
l
ab
ad
adg
adf
bcdgi
cdfgij
bcdgi
cdfgij
dfgij
fgik
l
ad
ade
dfgij
fgik
efgij
ghk
jk
ik
hik
k
l
log2rpm
Temperature
10
8
6
4
10
15
20
25
30

AT2G45970.1

cytochrome P450, family 86, subfamily A, polypeptide 8

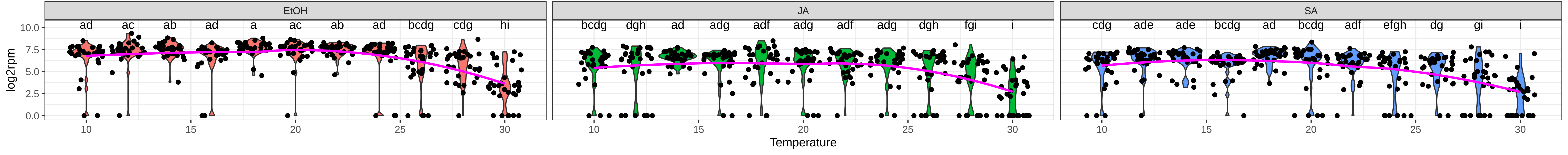

AT1G69440.1

Argonaute family protein

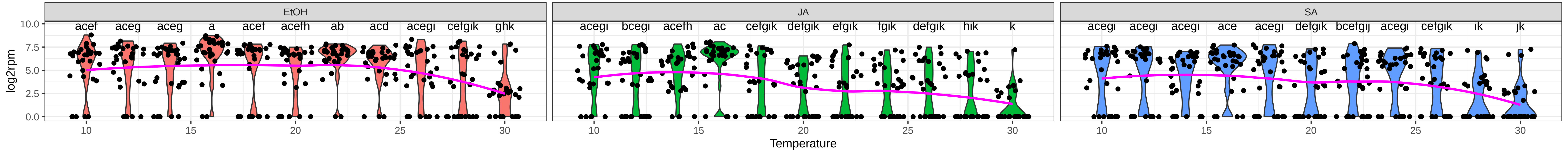

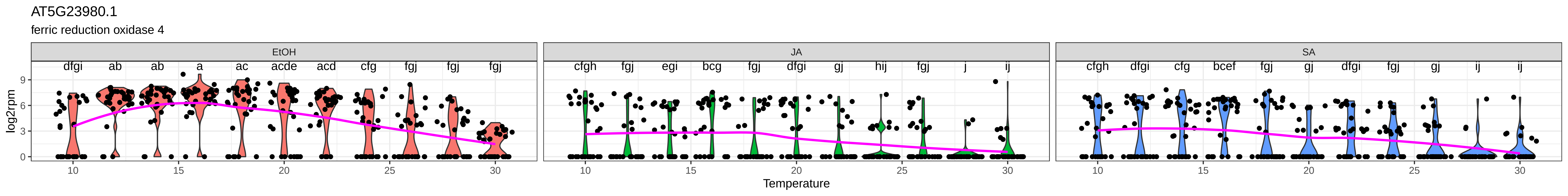
AT5G23980.1
ferric reduction oxidase 4
EtOH
JA
SA
dfgi ab ab a ac acde acd cfg fgj fgj fgj
cfgh fgj egi bcg fgj dfgi gj hij fgj j ij
cfgh dfgi cfg bcef fgj gj dfgi fgj gj ij ij
log2rpm
0
3
6
9
10
15
20
25
30
Temperature

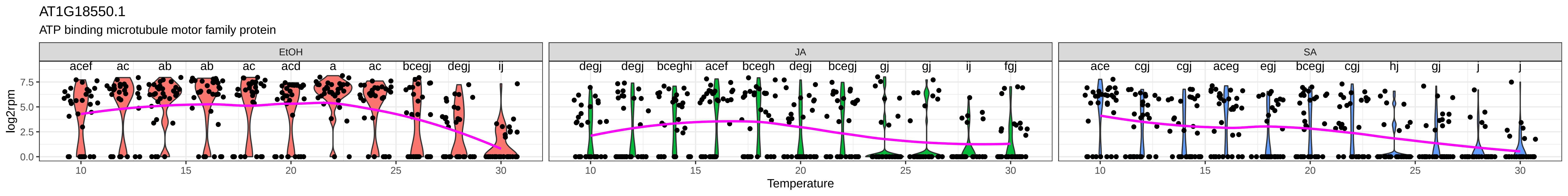
AT1G18550.1
ATP binding microtubule motor family protein
EtOH
JA
SA
acef ac ab ab ac acd a ac bcegj degj ij
degj degj bceghi acef bcegh degj bcegj gj gj ij fgj
ace cgj cgj aceg egj bcegj cgj hj gj j j
log2rpm
7.5
5.0
2.5
0.0
10
15
20
25
30
Temperature

AT5G60930.2

P−loop containing nucleoside triphosphate hydrolases superfamily protein

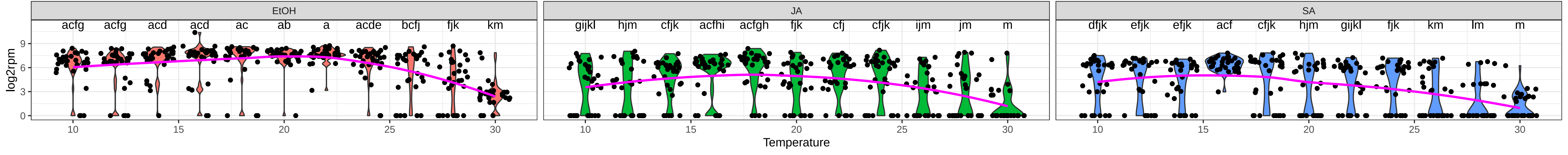

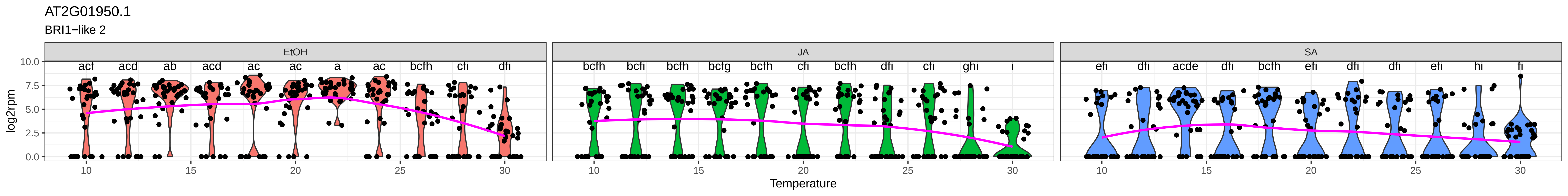

AT2G01950.1
BRI1−like 2
EtOH
JA
SA
acf
acd
ab
acd
ac
ac
a
ac
bcfh
cfi
dfi
bcfh
bcfi
bcfh
bcfg
bcfh
cfi
bcfh
dfi
cfi
ghi
i
efi
dfi
acde
dfi
bcfh
efi
dfi
dfi
efi
hi
fi
log2rpm
10.0
7.5
5.0
2.5
0.0
10
15
20
25
30
Temperature

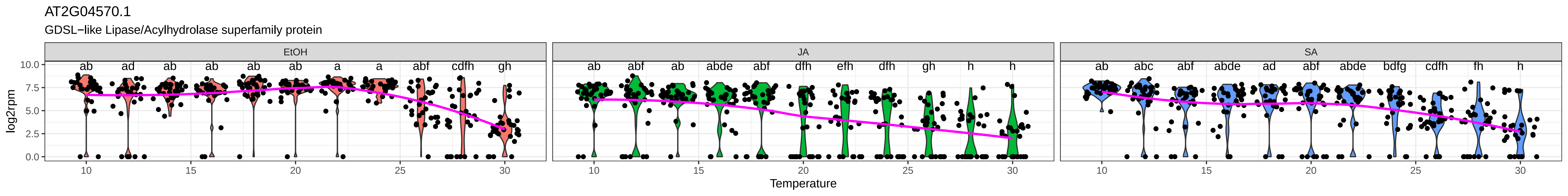
AT2G04570.1
GDSL−like Lipase/Acylhydrolase superfamily protein
EtOH
JA
SA
ab ad ab ab ab ab a a abf cdfh gh
ab abf ab abde abf dfh efh dfh gh h h
ab abc abf abde ad abf abde bdfg cdfh fh h
log2rpm
0.0
2.5
5.0
7.5
10.0
10 15 20 25 30
Temperature

AT4G38410.1

Dehydrin family protein

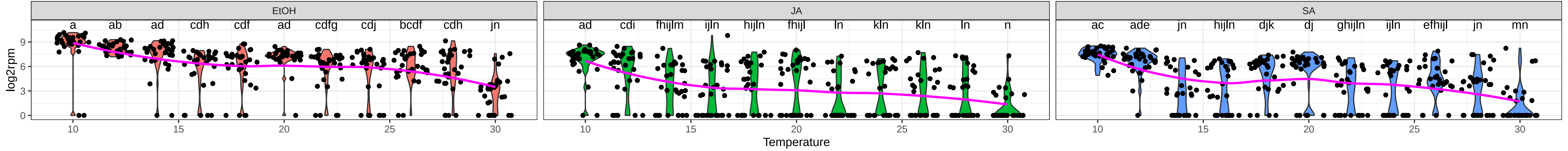

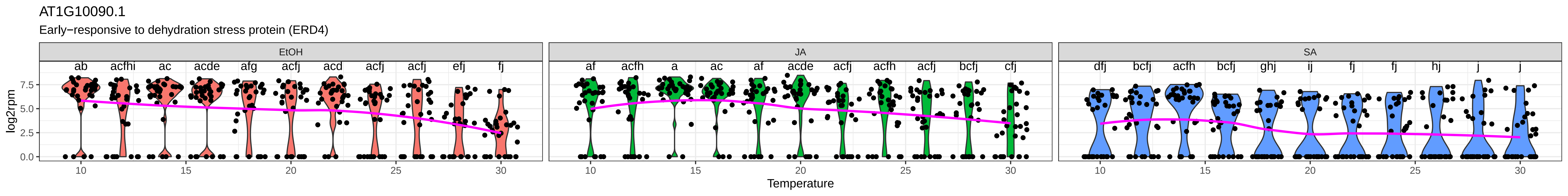
AT1G10090.1
Early−responsive to dehydration stress protein (ERD4)
EtOH
JA
SA
ab acfhi ac acde afg acfj acd acfj acfj efj fj
af acfh a ac af acde acfj acfh acfj bcfj cfj
dfj bcfj acfh bcfj ghj ij fj fj hj j j
log2rpm
0.0
2.5
5.0
7.5
10
15
20
25
30
Temperature

AT3G15460.1

Ribosomal RNA processing Brix domain protein

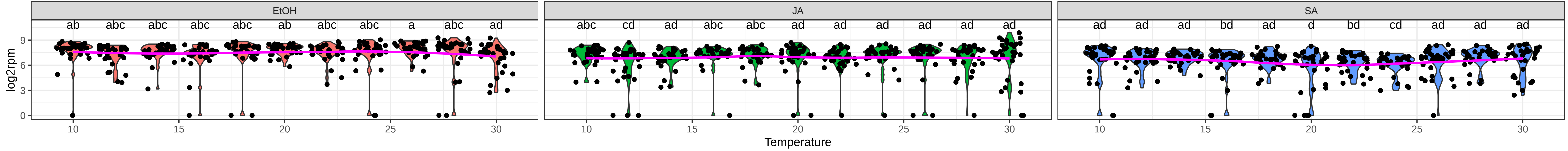

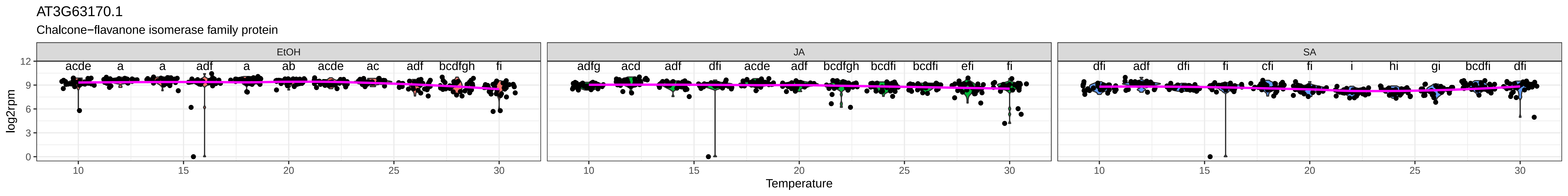
AT3G63170.1
Chalcone−flavanone isomerase family protein
EtOH
JA
SA
acde a a adf a ab acde ac adf bcdfgh fi
adfg acd adf dfi acde adf bcdfgh bcdfi bcdfi efi fi
dfi adf dfi fi cfi fi i hi gi bcdfi dfi
log2rpm
0
3
6
9
12
10
15
20
25
30
Temperature

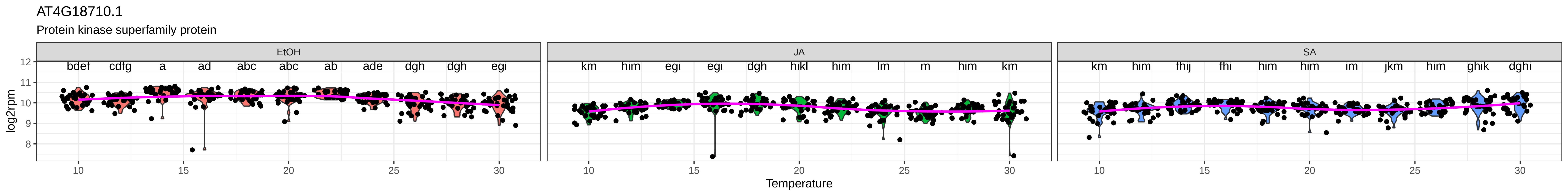

AT4G18710.1
Protein kinase superfamily protein
EtOH
JA
SA
bdef cdfg a ad abc abc ab ade dgh dgh egi
km him egi egi dgh hikl him lm m him km
km him fhij fhi him him im jkm him ghik dghi
log2rpm
12
11
10
9
8
10
15
20
25
30
Temperature

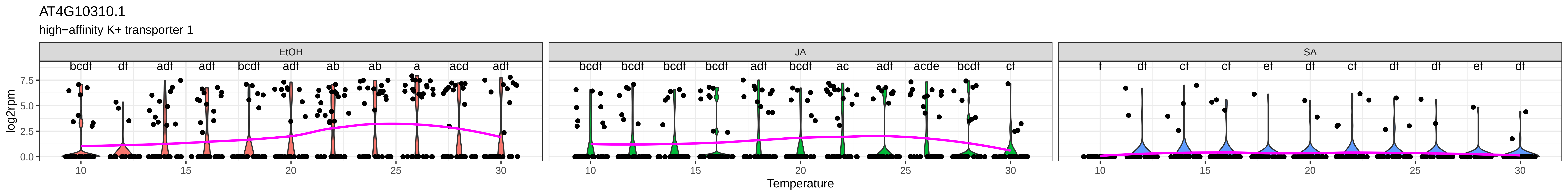
AT4G10310.1
high−affinity K+ transporter 1
EtOH
JA
SA
bcdf df adf adf bcdf adf ab ab a acd adf
bcdf bcdf bcdf bcdf adf bcdf ac adf acde bcdf cf
f df cf cf ef df cf df df ef df
log2rpm
7.5
5.0
2.5
0.0
10 15 20 25 30
Temperature

AT3G16350.1

Homeodomain−like superfamily protein

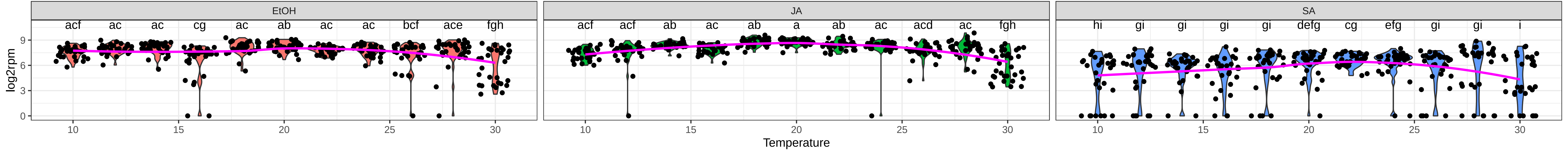

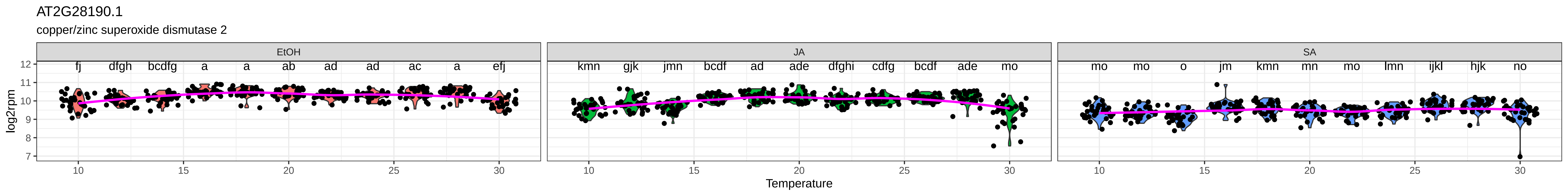

AT2G28190.1
copper/zinc superoxide dismutase 2
EtOH
JA
SA
fj dfgh bcdfg a a ab ad ad ac a efj
kmn gjk jmn bcdf ad ade dfghi cdfg bcdf ade mo
mo mo o jm kmn mn mo lmn ijkl hjk no
log2rpm
12
11
10
9
8
7
10
15
20
25
30
Temperature

AT1G23720.2

Proline−rich extensin−like family protein

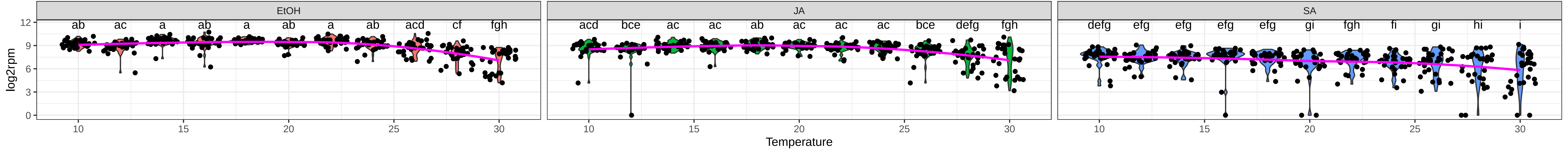

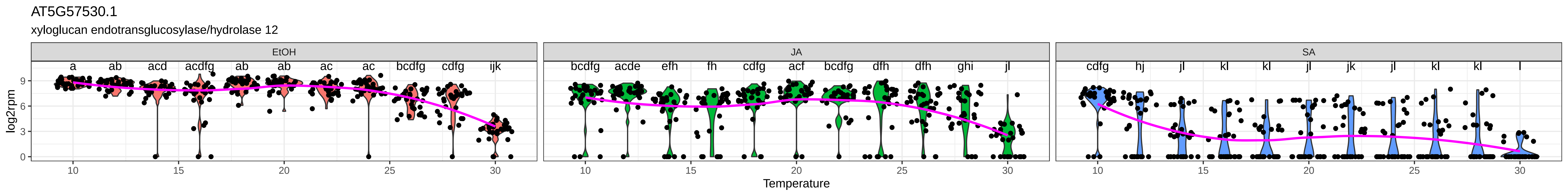
AT5G57530.1
xyloglucan endotransglucosylase/hydrolase 12
EtOH
JA
SA
a ab acd acdfg ab ab ac ac bcdfg cdfg ijk
bcdfg acde efh fh cdfg acf bcdfg dfh dfh ghi jl
cdfg hj jl kl kl jl jk jl kl kl l
log2rpm
0
3
6
9
10
15
20
25
30
Temperature

AT5G13480.3

Transducin/WD40 repeat−like superfamily protein

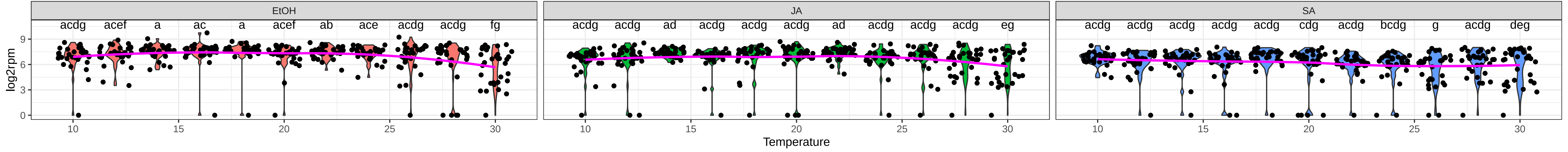

AT2G38120.2

Transmembrane amino acid transporter family protein

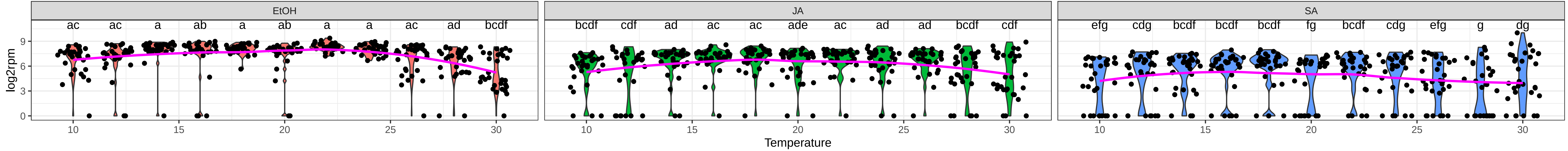

AT4G00710.1

BR−signaling kinase 3

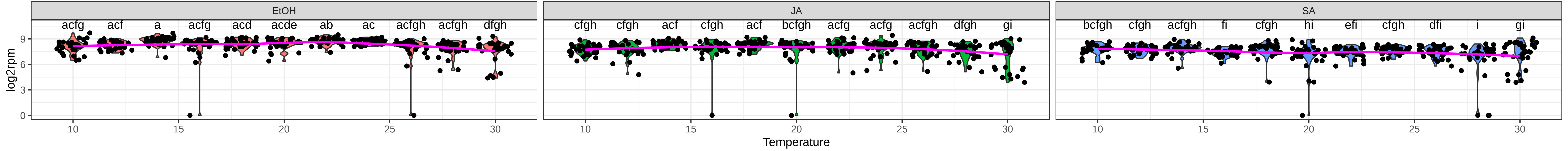

AT4G04885.1

PCF11P−similar protein 4

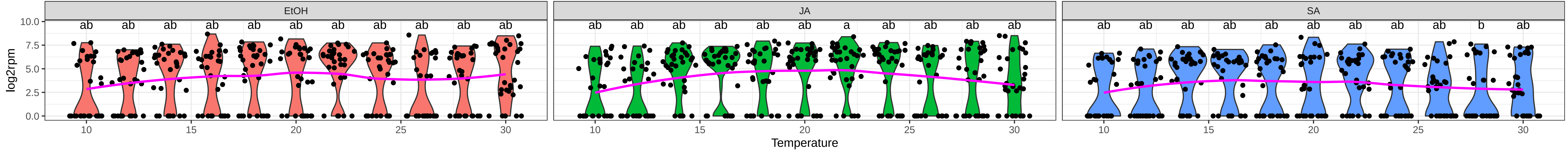

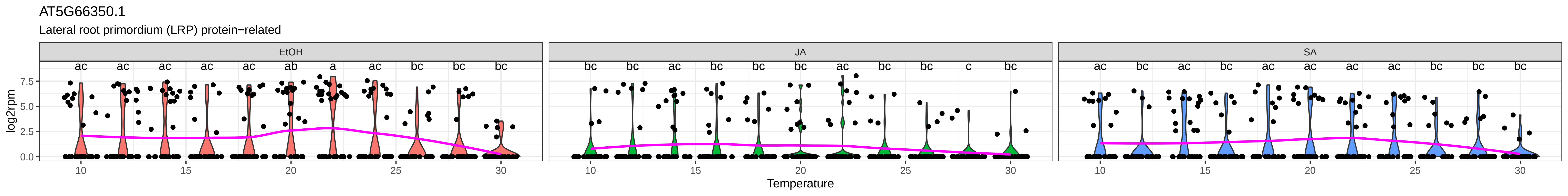
AT5G66350.1
Lateral root primordium (LRP) protein−related
EtOH
JA
SA
ac ac ac ac ac ab a ac bc bc bc
bc bc ac bc bc bc ac bc bc c bc
ac bc ac bc ac ac ac ac bc bc bc
log2rpm
7.5
5.0
2.5
0.0
10
15
20
25
30
Temperature

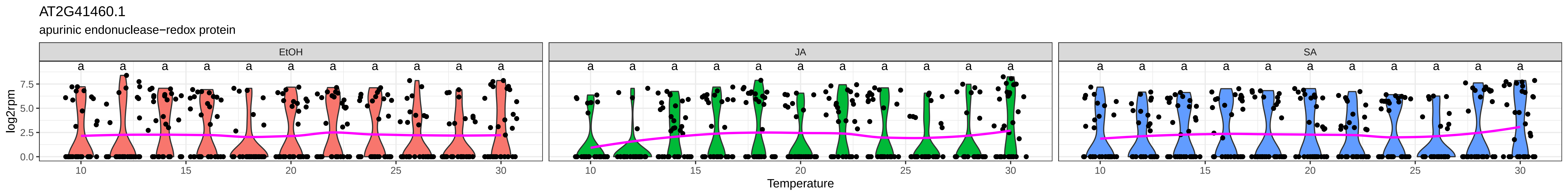

AT2G41460.1
apurinic endonuclease−redox protein
EtOH
JA
SA
a
log2rpm
0.0
2.5
5.0
7.5
10
15
20
25
30
Temperature

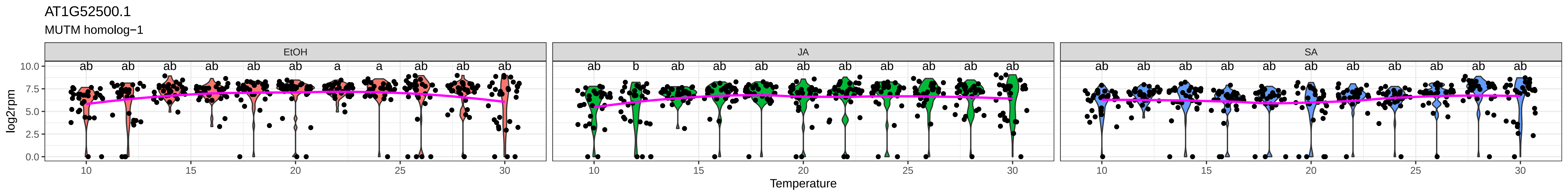
AT1G52500.1
MUTM homolog−1
EtOH
JA
SA
log2rpm
Temperature
ab b a

AT3G19840.1

pre−mRNA−processing protein 40C

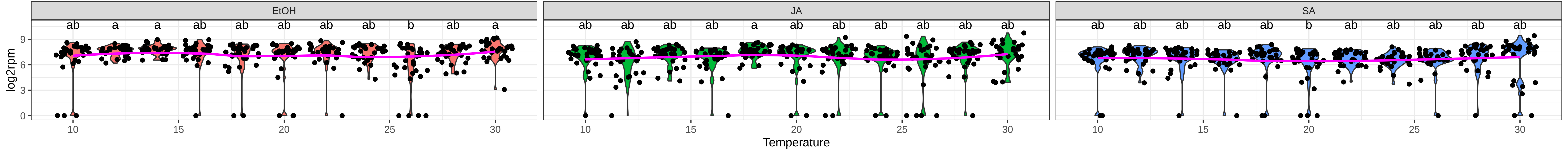

AT2G28290.2

P−loop containing nucleoside triphosphate hydrolases superfamily protein

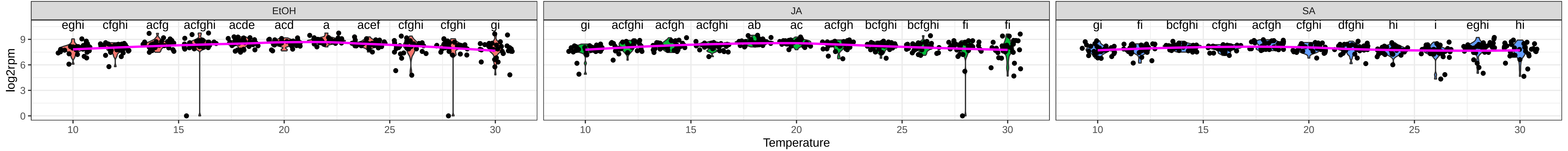

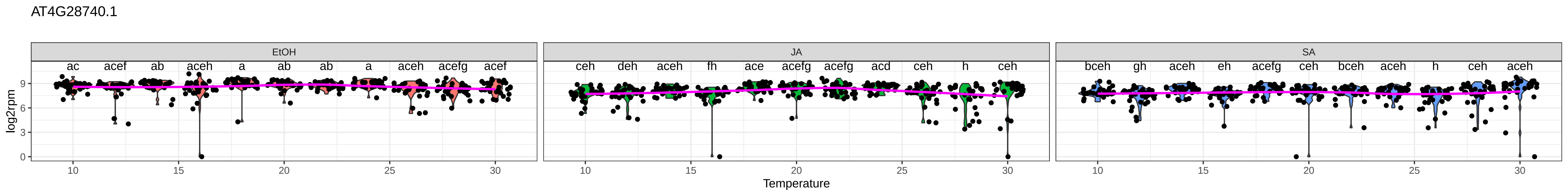
AT4G28740.1
EtOH
JA
SA
ac acef ab aceh a ab ab a aceh acefg acef
ceh deh aceh fh ace acefg acefg acd ceh h ceh
bceh gh aceh eh acefg ceh bceh aceh h ceh aceh
log2rpm
0
3
6
9
10
15
20
25
30
Temperature
