# Supplementary material for: DeLTa-Seq: direct-lysate targeted RNA-Seq from crude tissue lysate
Source: Plant Methods. 2022 Aug 6;18:99. doi: 10.1186/s13007-022-00930-x (PMC9356424; doi:10.1186/s13007-022-00930-x)

AT5G44420.1

plant defensin 1.2

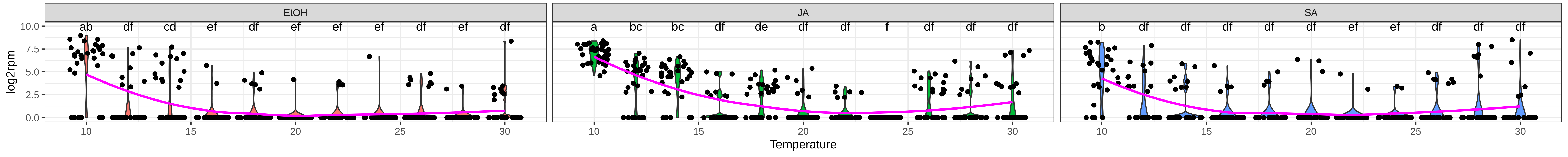

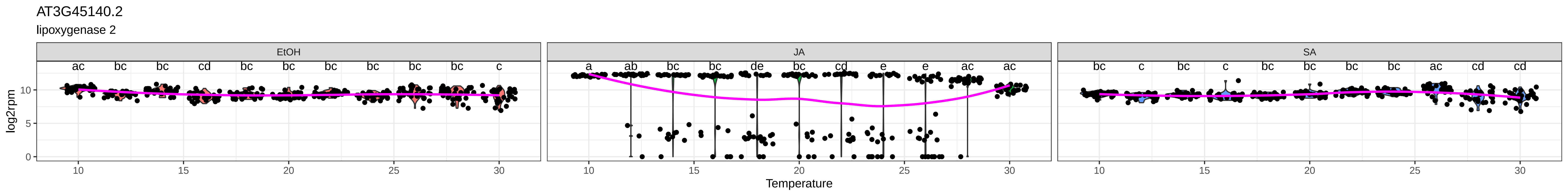
AT3G45140.2
lipoxygenase 2
EtOH
JA
SA
ac bc bc cd bc bc bc bc bc bc c
a ab bc bc de bc cd e e ac ac
bc c bc c bc bc bc bc ac cd cd
log2rpm
0
5
10
10
15
20
25
30
Temperature

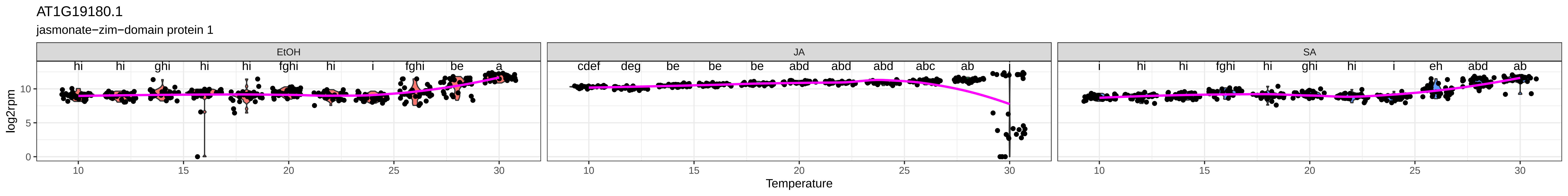
AT1G19180.1
jasmonate−zim−domain protein 1
EtOH
JA
SA
hi hi ghi hi hi fghi hi i fghi be a
cdef deg be be be abd abd abd abc ab i
i hi hi fghi hi ghi hi i eh abd ab
log2rpm
0
5
10
10 15 20 25 30
Temperature

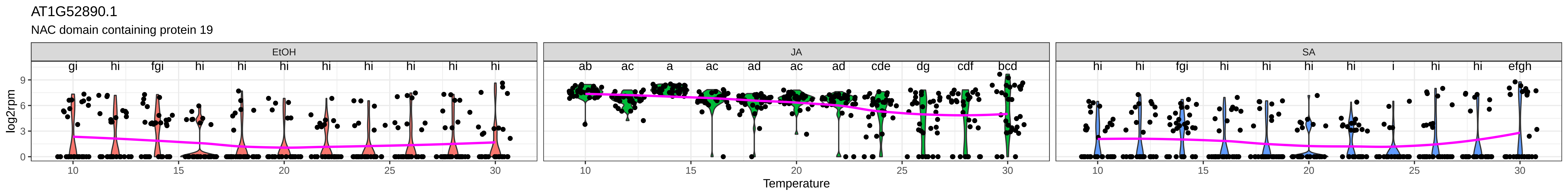
AT1G52890.1
NAC domain containing protein 19
EtOH
JA
SA
gi hi fgi hi hi hi hi hi hi hi hi
ab ac a ac ad ac ad cde dg cdf bcd
hi hi fgi hi hi hi hi i hi hi efgh
log2rpm
0
3
6
9
10
15
20
25
30
Temperature

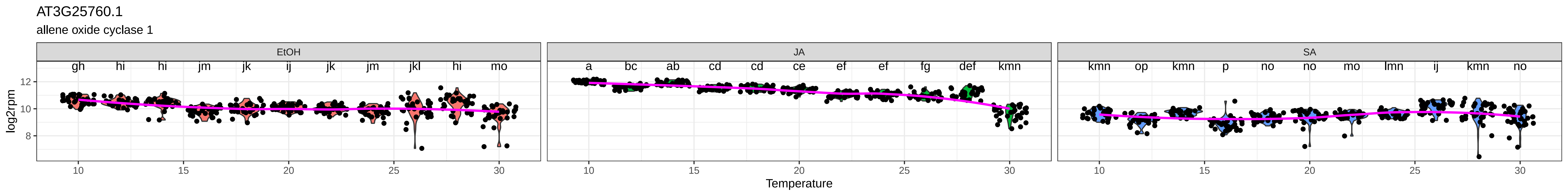
AT3G25760.1
allene oxide cyclase 1
EtOH
JA
SA
gh
hi
hi
jm
jk
ij
jk
jm
jkl
hi
mo
a
bc
ab
cd
cd
ce
ef
ef
fg
def
kmn
kmn
op
kmn
p
no
no
mo
lmn
ij
kmn
no
log2rpm
12
10
8
10
15
20
25
30
Temperature

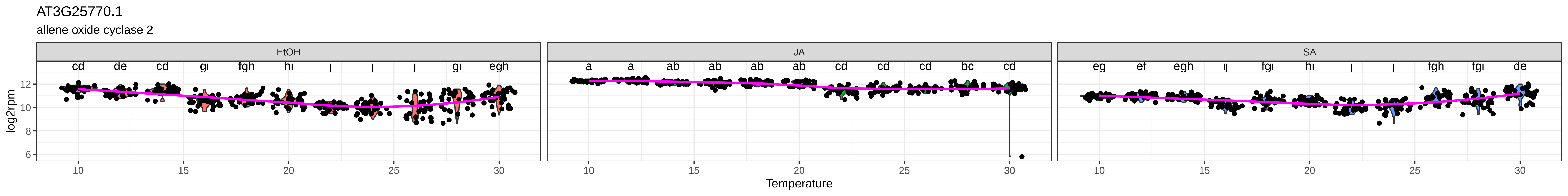
AT3G25770.1
allene oxide cyclase 2
EtOH
JA
SA
cd de cd gi fgh hi j j j gi egh
a a ab ab ab ab cd cd cd bc cd
eg ef egh ij fgi hi j j fgh fgi de
log2rpm
12
10
8
6
10
15
20
25
30
Temperature

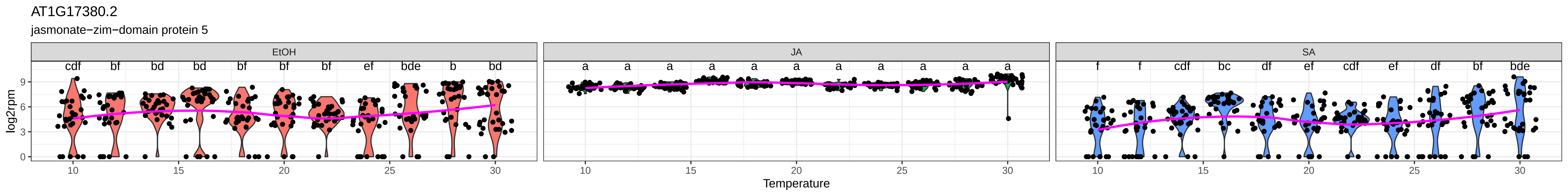

AT1G17380.2
jasmonate−zim−domain protein 5
EtOH
JA
SA
cdf bf bd bd bf bf bf ef bde b bd
a a a a a a a a a a a
f f cdf bc df ef cdf ef df bf bde
log2rpm
0
3
6
9
10
15
20
25
30
Temperature

AT5G24780.1

vegetative storage protein 1

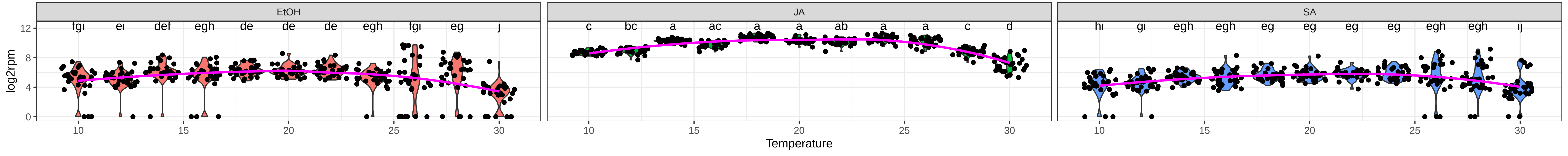

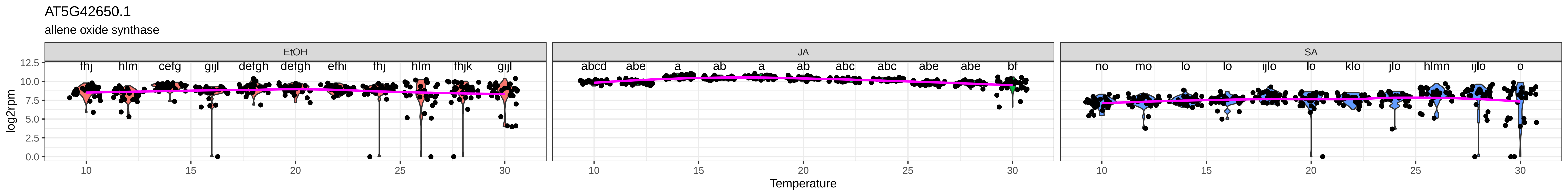
AT5G42650.1
allene oxide synthase
EtOH
JA
SA
fhj
hlm
cefg
gijl
defgh
defgh
efhi
fhj
hlm
fhjk
gijl
abcd
abe
a
ab
a
ab
abc
abc
abe
abe
bf
no
mo
lo
lo
ijlo
lo
klo
jlo
hlmn
ijlo
o
log2rpm
12.5
10.0
7.5
5.0
2.5
0.0
10
15
20
25
30
Temperature

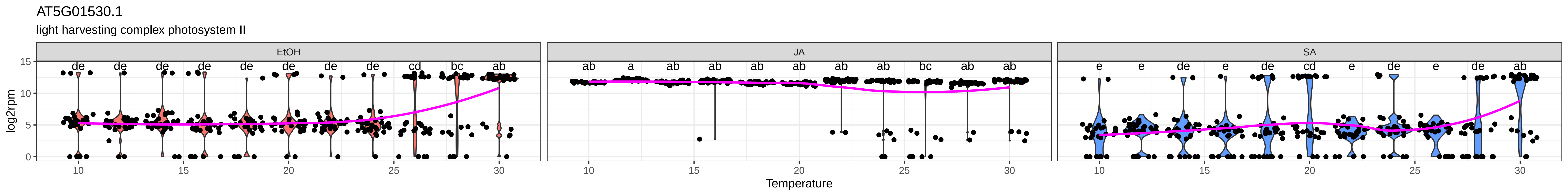

AT5G01530.1
light harvesting complex photosystem II
EtOH
JA
SA
de de de de de de de de cd bc ab
ab a ab ab ab ab ab ab bc ab ab
e e de e de cd e de e de ab
log2rpm
0
5
10
15
10
15
20
25
30
Temperature

AT2G27690.1

cytochrome P450, family 94, subfamily C, polypeptide 1

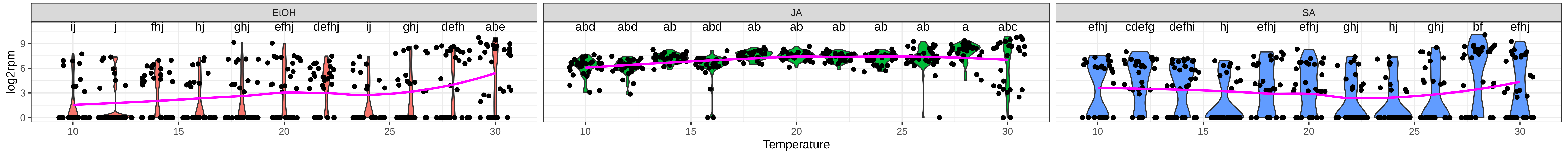

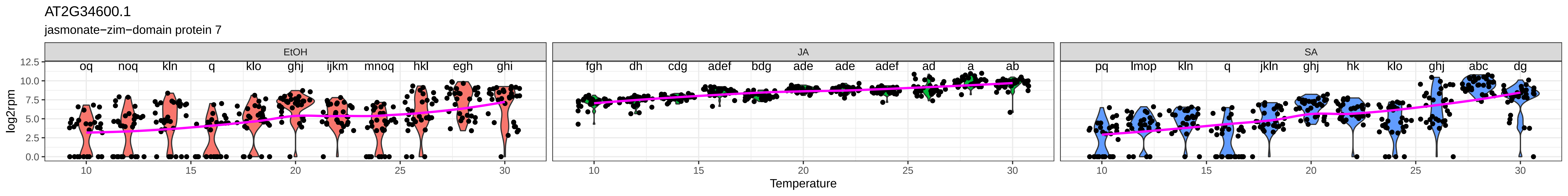

AT2G34600.1
jasmonate−zim−domain protein 7
EtOH
JA
SA
oq noq kln q klo ghj ijkm mnoq hkl egh ghi
fgh dh cdg adef bdg ade ade adef ad a ab
pq lmop kln q jkln ghj hk klo ghj abc dg
log2rpm
0.0
2.5
5.0
7.5
10.0
12.5
10
15
20
25
30
Temperature

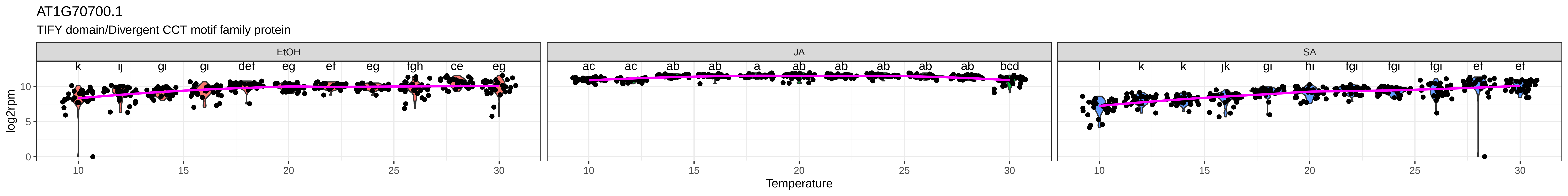

AT1G70700.1
TIFY domain/Divergent CCT motif family protein
EtOH
JA
SA
k
ij
gi
gi
def
eg
ef
eg
fgh
ce
eg
ac
ac
ab
ab
a
ab
ab
ab
ab
ab
bcd
l
k
k
jk
gi
hi
fgi
fgi
fgi
ef
ef
log2rpm
0
5
10
10
15
20
25
30
Temperature

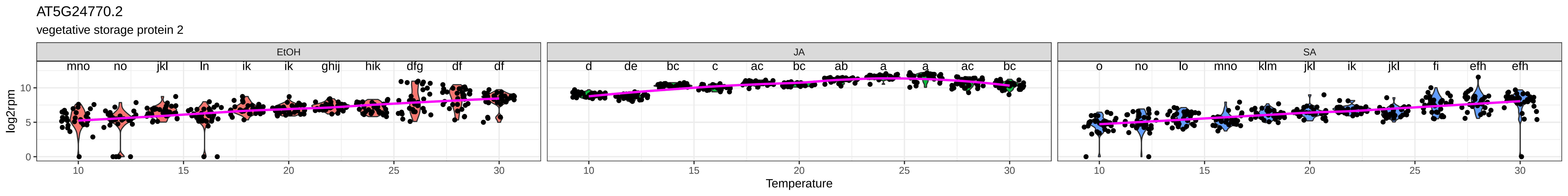

AT5G24770.2
vegetative storage protein 2
EtOH
JA
SA
mno
no
jkl
ln
ik
ik
ghij
hik
dfg
df
df
d
de
bc
c
ac
bc
ab
a
a
ac
bc
o
no
lo
mno
klm
jkl
ik
jkl
fi
efh
efh
log2rpm
0
5
10
10
15
20
25
30
Temperature

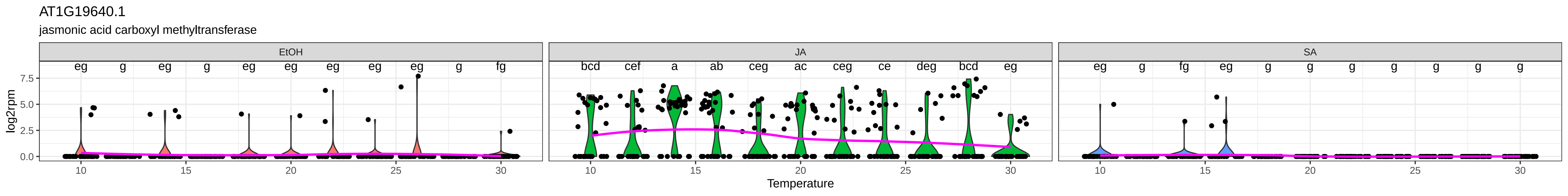

AT1G19640.1
jasmonic acid carboxyl methyltransferase
EtOH
JA
SA
eg g eg g eg eg eg eg eg g fg
bcd cef a ab ceg ac ceg ce deg bcd eg
eg g fg eg g g g g g g g
log2rpm
0.0
2.5
5.0
7.5
10 15 20 25 30
Temperature

AT4G27410.2

NAC (No Apical Meristem) domain transcriptional regulator superfamily protein

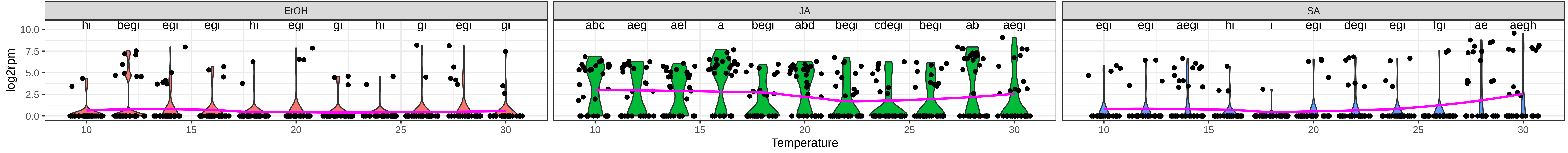

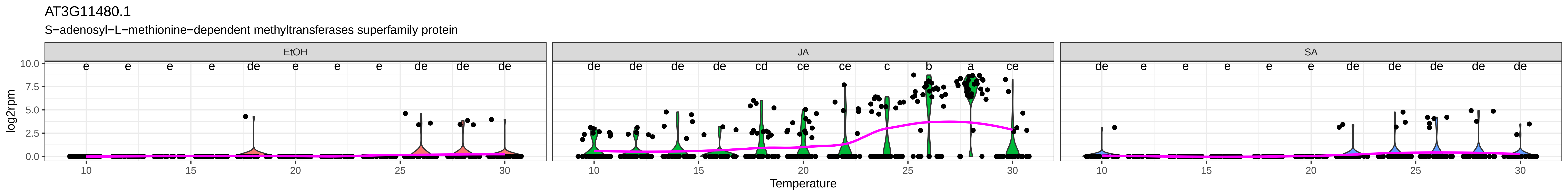

AT3G11480.1
S−adenosyl−L−methionine−dependent methyltransferases superfamily protein
EtOH
JA
SA
e e e e de e e e de de de
de de de de cd ce ce c b a ce
de e e e e e de de de de de
log2rpm
0.0
2.5
5.0
7.5
10.0
10
15
20
25
30
Temperature

AT5G13220.6

jasmonate−zim−domain protein 10

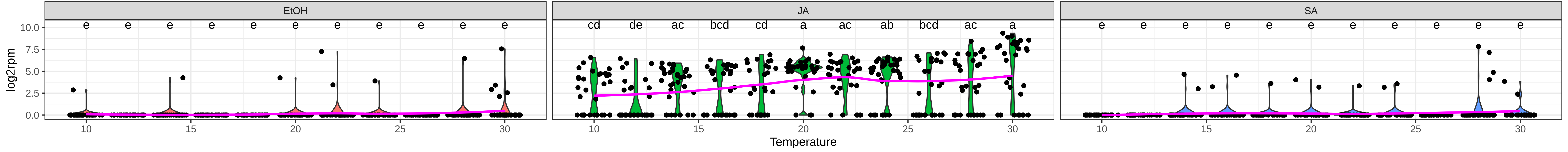

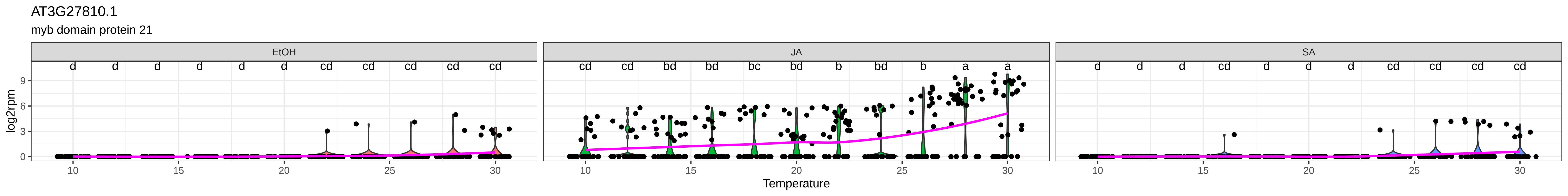

AT3G27810.1
myb domain protein 21
EtOH
JA
SA
d d d d d d cd cd cd cd cd
cd cd bd bd bc bd b bd b a a
d d d cd d d d cd cd cd cd
log2rpm
0
3
6
9
10
15
20
25
30
Temperature

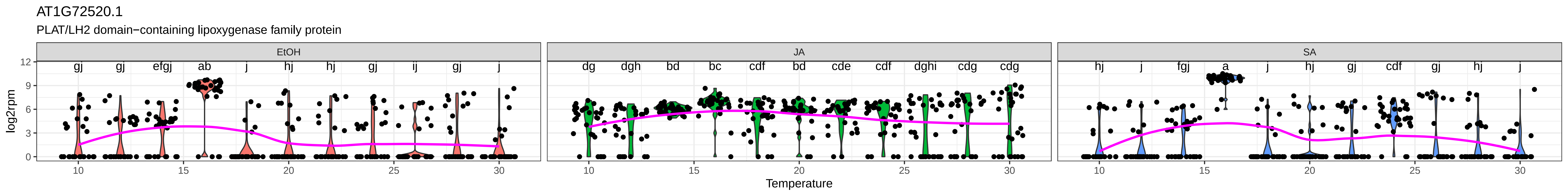

AT1G72520.1
PLAT/LH2 domain−containing lipoxygenase family protein
EtOH
JA
SA
log2rpm
Temperature
gj gj efgj ab j hj hj gj ij gj j
dg dgh bd bc cdf bd cde cdf dghi cdg cdg
hj j fgj a j hj gj cdf gj hj j
0
3
6
9
12
10
15
20
25
30

AT1G17420.1

lipoxygenase 3

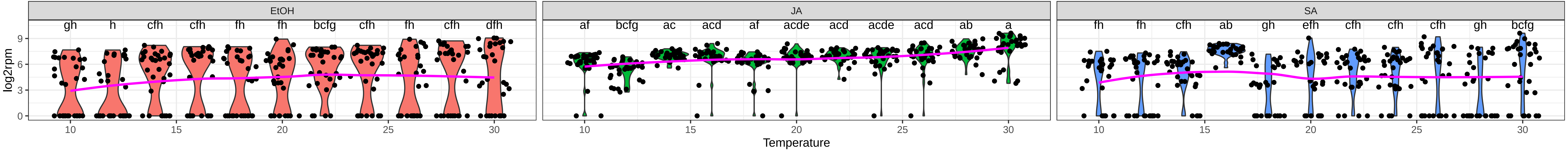

AT1G74950.2

TIFY domain/Divergent CCT motif family protein

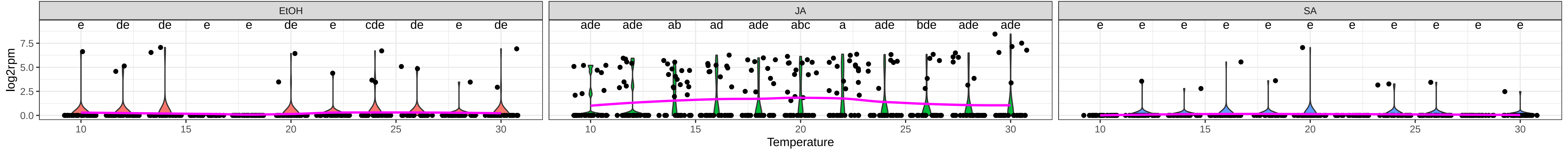

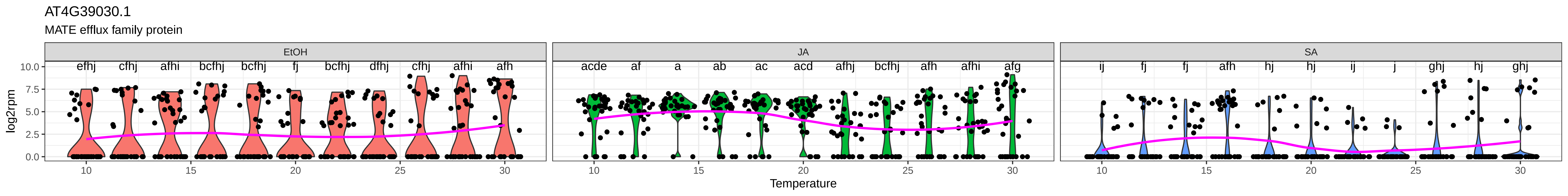

AT4G39030.1
MATE efflux family protein
EtOH
JA
SA
efhj cfhj afhi bcfhj bcfhj fj bcfhj dfhj cfhj afhi afh
acde af a ab ac acd afhj bcfhj afh afhi afg
ij fj fj afh hj hj ij j ghj hj ghj
log2rpm
0.0
2.5
5.0
7.5
10.0
10
15
20
25
30
Temperature

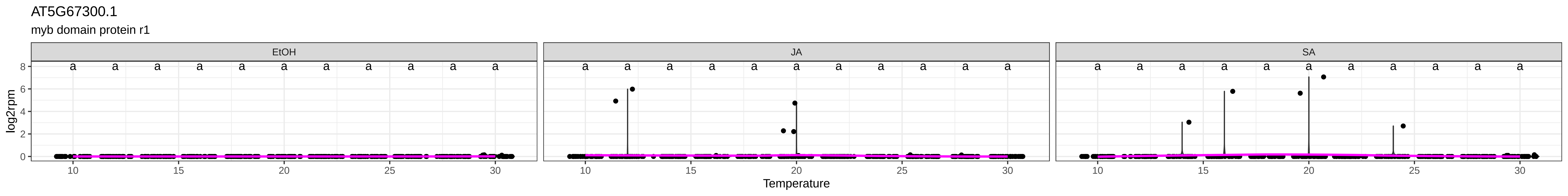

AT5G67300.1
myb domain protein r1
EtOH
JA
SA
log2rpm
Temperature

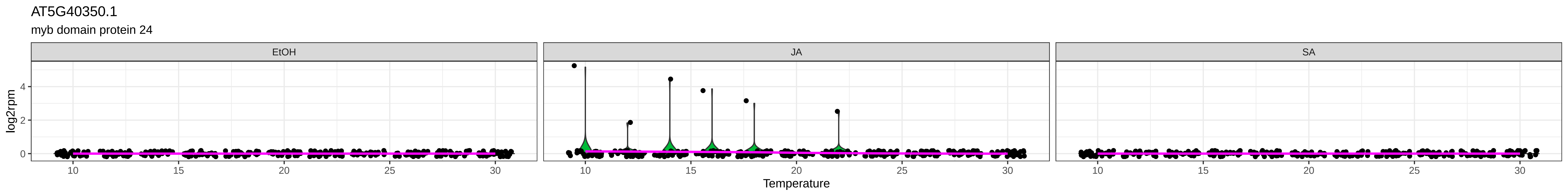
AT5G40350.1
myb domain protein 24
EtOH
JA
SA
log2rpm
0
2
4
10
15
20
25
30
Temperature

AT1G32640.1

Basic helix−loop−helix (bHLH) DNA−binding family protein

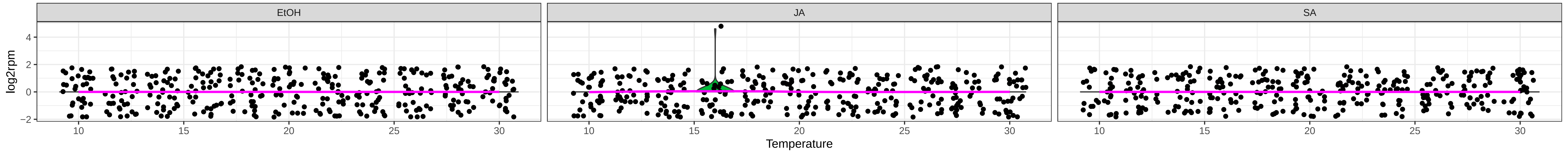

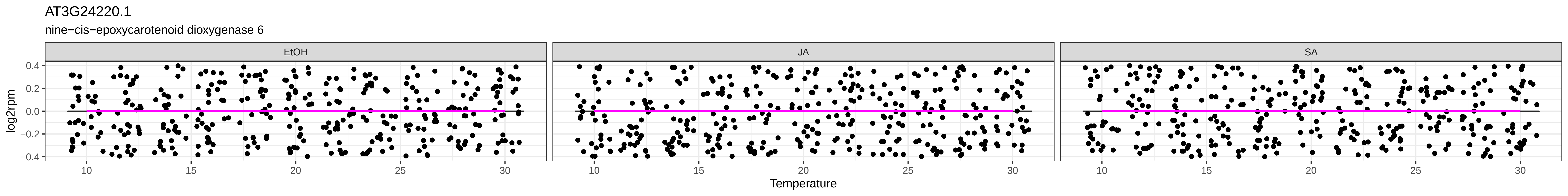
AT3G24220.1
nine−cis−epoxycarotenoid dioxygenase 6
EtOH
JA
SA
log2rpm
0.4
0.2
0.0
−0.2
−0.4
10
15
20
25
30
Temperature

AT2G44810.2

alpha/beta−Hydrolases superfamily protein

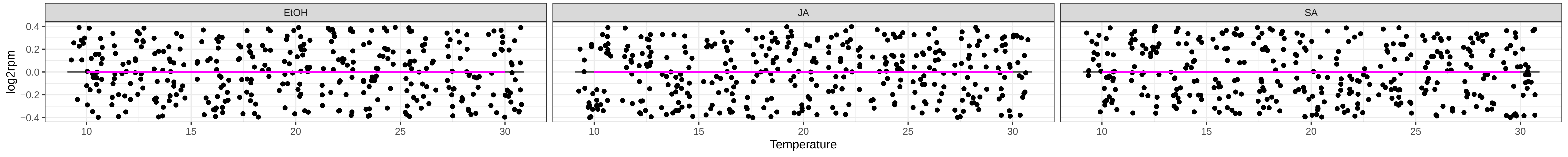

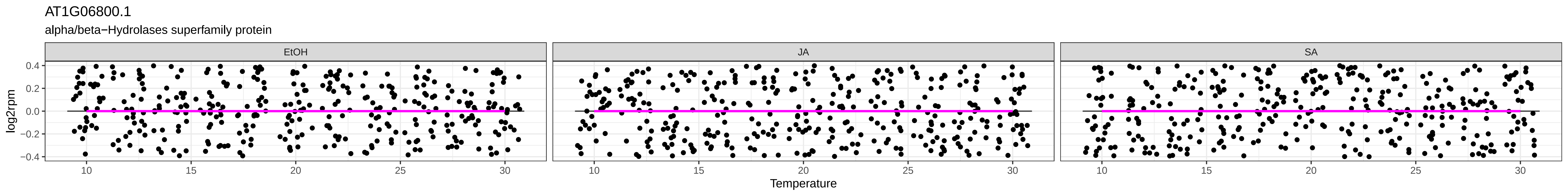
AT1G06800.1
alpha/beta−Hydrolases superfamily protein
EtOH
JA
SA
log2rpm
0.4
0.2
0.0
−0.2
−0.4
10
15
20
25
30
Temperature

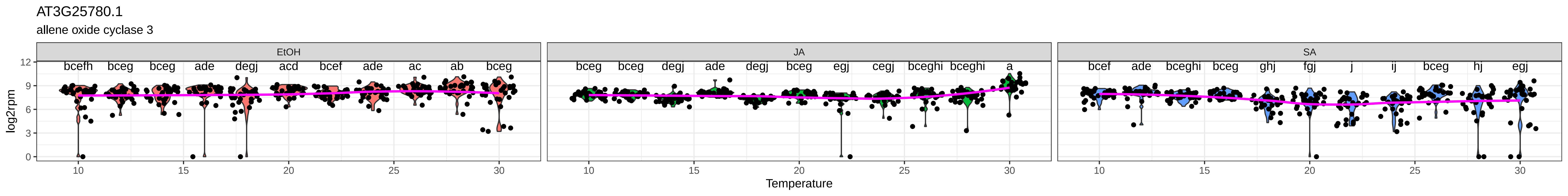

AT3G25780.1
allene oxide cyclase 3
EtOH
JA
SA
bcefh bceg bceg ade degj acd bcef ade ac ab bceg
bceg bceg degj ade degj bceg egj cegj bceghi bceghi a
bcef ade bceghi bceg ghj fgj j ij bceg hj egj
log2rpm
0
3
6
9
12
10
15
20
25
30
Temperature

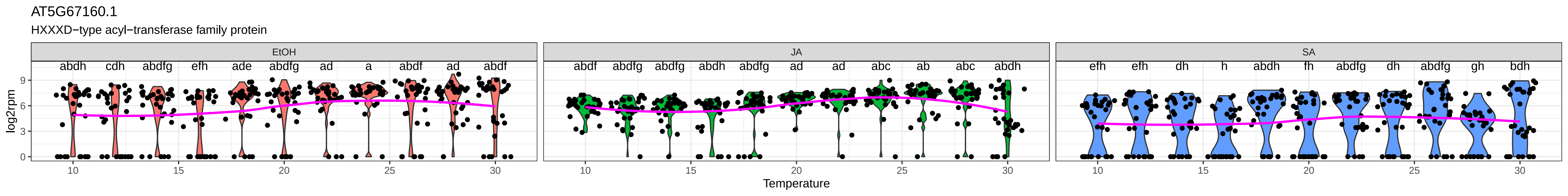
AT5G67160.1
HXXXD−type acyl−transferase family protein
EtOH
JA
SA
abdh cdh abdfg efh ade abdfg ad a abdf ad abdf
abdf abdfg abdfg abdh abdfg ad ad abc ab abc abdh
efh efh dh h abdh fh abdfg dh abdfg gh bdh
log2rpm
Temperature
0
3
6
9
10
15
20
25
30

AT1G13280.1

allene oxide cyclase 4

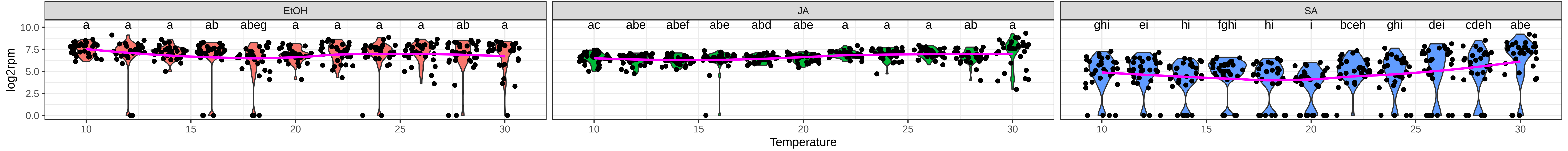

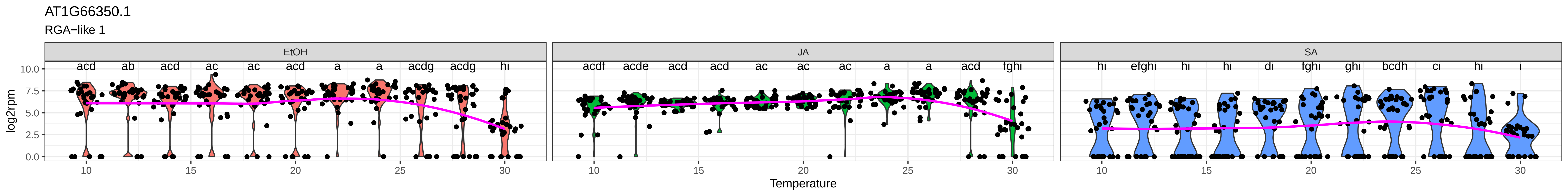

AT1G66350.1
RGA−like 1
EtOH
JA
SA
acd ab acd ac ac acd a a acdg acdg hi
acdf acde acd acd ac ac ac a a acd fghi
hi efghi hi hi di fghi ghi bcdh ci hi i
log2rpm
0.0
2.5
5.0
7.5
10.0
10 15 20 25 30
Temperature

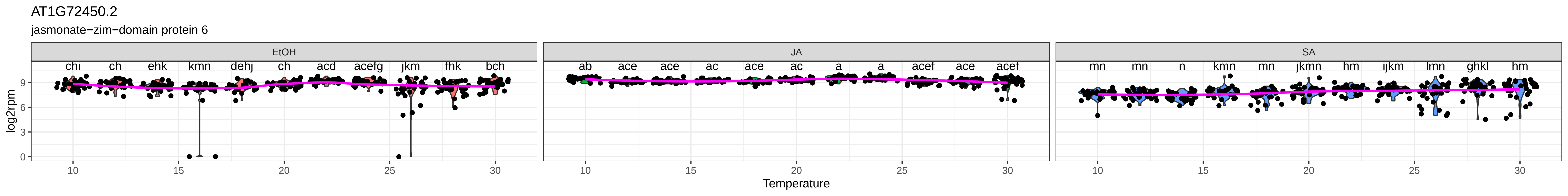
AT1G72450.2
jasmonate−zim−domain protein 6
EtOH
JA
SA
chi
ch
ehk
kmn
dehj
ch
acd
acefg
jkm
fhk
bch
ab
ace
ace
ac
ace
ac
a
a
acef
ace
acef
mn
mn
n
kmn
mn
jkmn
hm
ijkm
lmn
ghkl
hm
log2rpm
Temperature

AT1G06160.1

octadecanoid−responsive Arabidopsis AP2/ERF 59

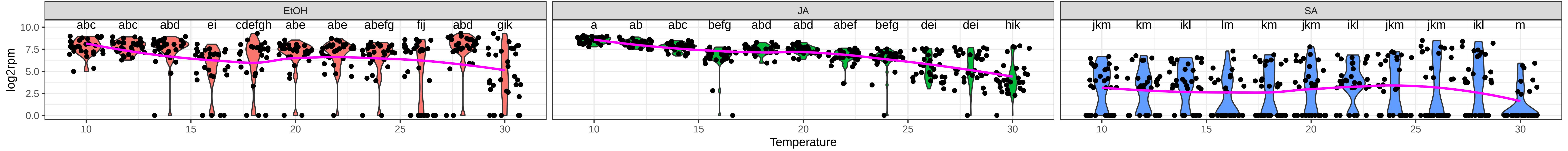

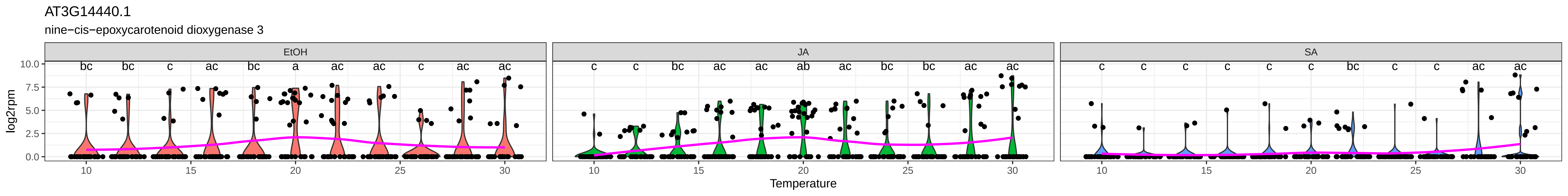

AT3G14440.1
nine−cis−epoxycarotenoid dioxygenase 3
EtOH
JA
SA
bc bc c ac bc a ac ac c ac ac
c c bc ac ac ab ac bc bc ac ac
c c c c c c bc c c ac ac
log2rpm
0.0
2.5
5.0
7.5
10.0
10
15
20
25
30
Temperature

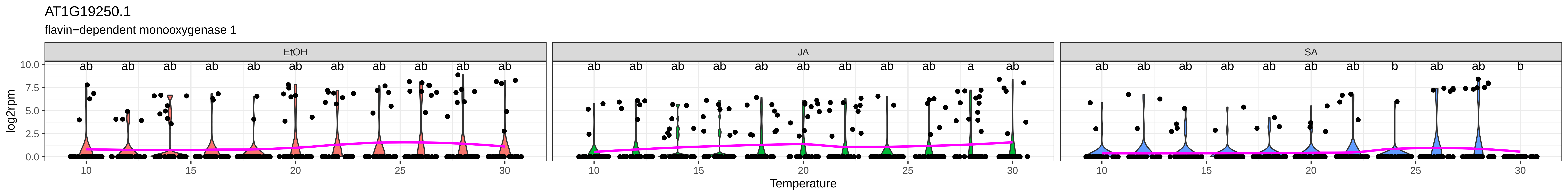
AT1G19250.1
flavin−dependent monooxygenase 1
EtOH
JA
SA
log2rpm
Temperature
10.0
7.5
5.0
2.5
0.0
10
15
20
25
30
ab ab ab ab ab ab ab ab ab ab ab
ab ab ab ab ab ab ab ab ab a ab
ab ab ab ab ab ab ab b ab ab b

AT1G67560.1

PLAT/LH2 domain−containing lipoxygenase family protein

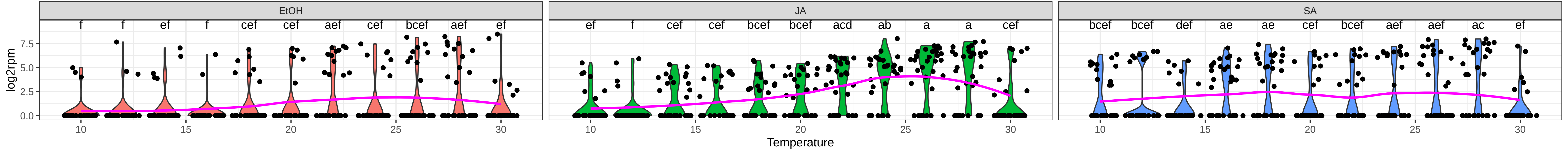

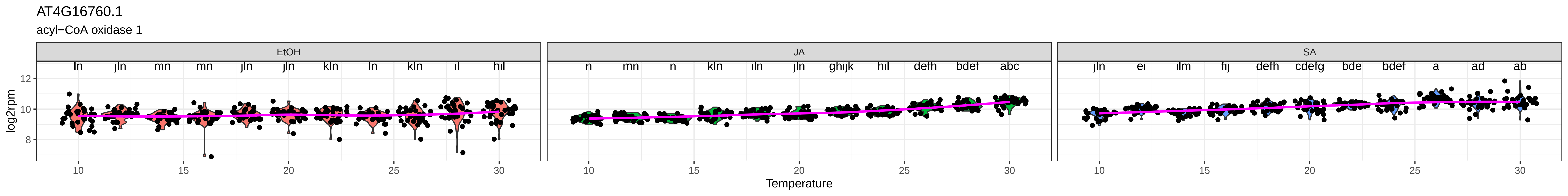

AT4G16760.1
acyl−CoA oxidase 1
EtOH
JA
SA
ln
jln
mn
mn
jln
jln
kln
ln
kln
il
hil
n
mn
n
kln
iln
jln
ghijk
hil
defh
bdef
abc
jln
ei
ilm
fij
defh
cdefg
bde
bdef
a
ad
ab
log2rpm
12
10
8
10
15
20
25
30
Temperature

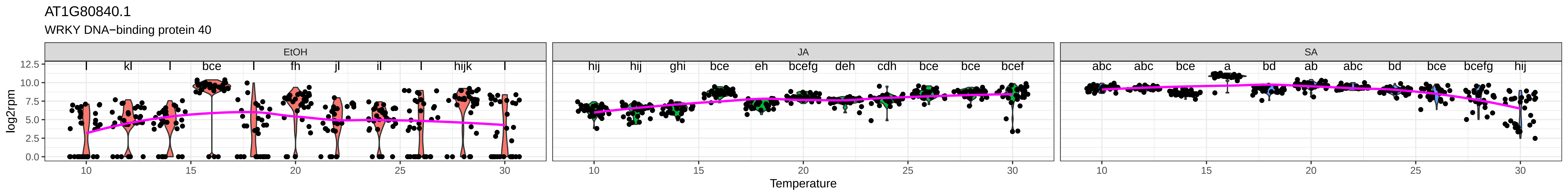

AT1G80840.1
WRKY DNA−binding protein 40
EtOH
JA
SA
l
kl
l
bce
l
fh
jl
il
l
hijk
l
hij
hij
ghi
bce
eh
bcefg
deh
cdh
bce
bce
bcef
abc
abc
bce
a
bd
ab
abc
bd
bce
bcefg
hij
log2rpm
12.5
10.0
7.5
5.0
2.5
0.0
10
15
20
25
30
Temperature

AT1G28480.1

Thioredoxin superfamily protein

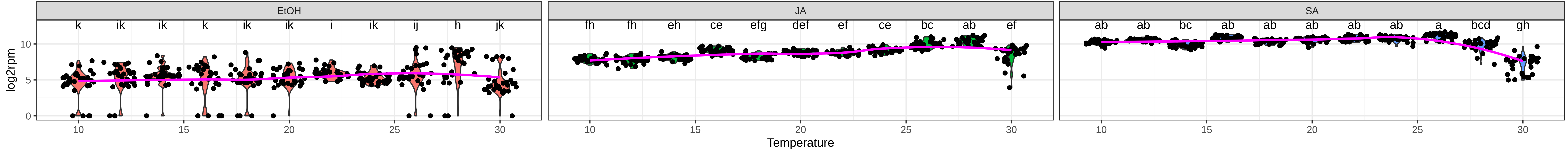

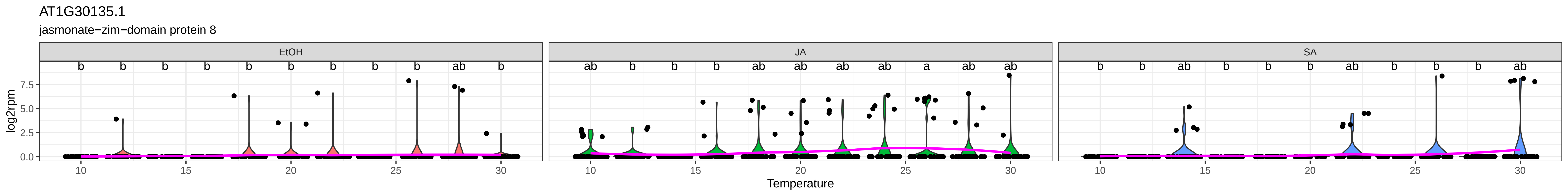
AT1G30135.1
jasmonate−zim−domain protein 8
EtOH
JA
SA
b b b b b b b b b ab b
ab b b b ab ab ab ab a ab ab
b b ab b b b ab b b b ab
log2rpm
7.5
5.0
2.5
0.0
10 15 20 25 30
Temperature

AT3G17860.2

jasmonate−zim−domain protein 3

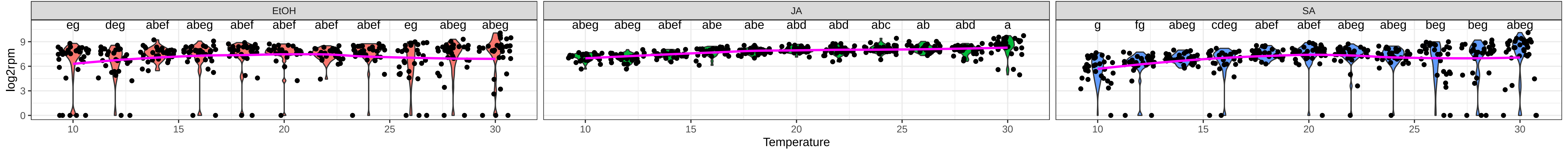

AT2G06050.1

oxophytodienoate−reductase 3

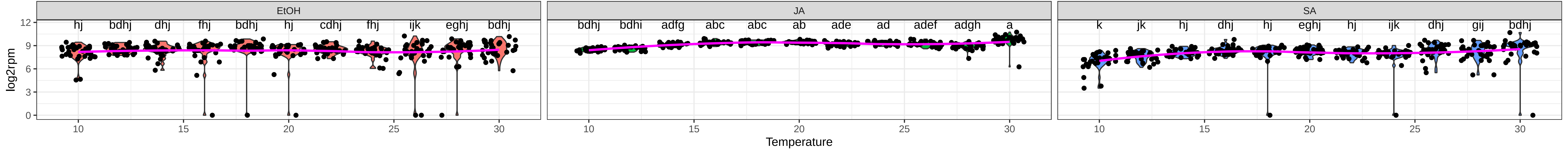

AT3G23240.1

ethylene response factor 1

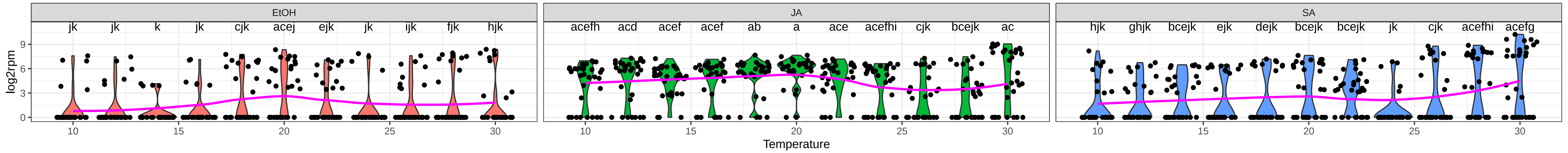

AT3G48520.1

cytochrome P450, family 94, subfamily B, polypeptide 3

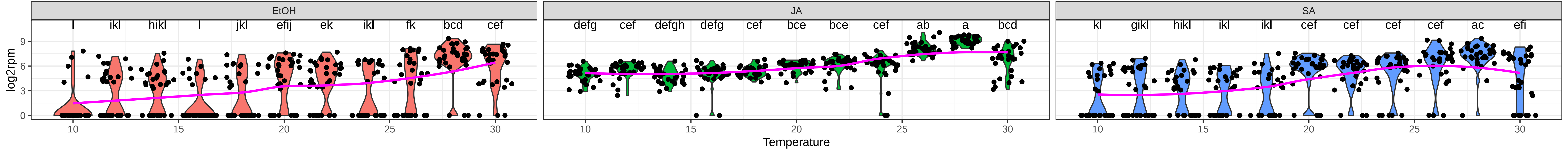

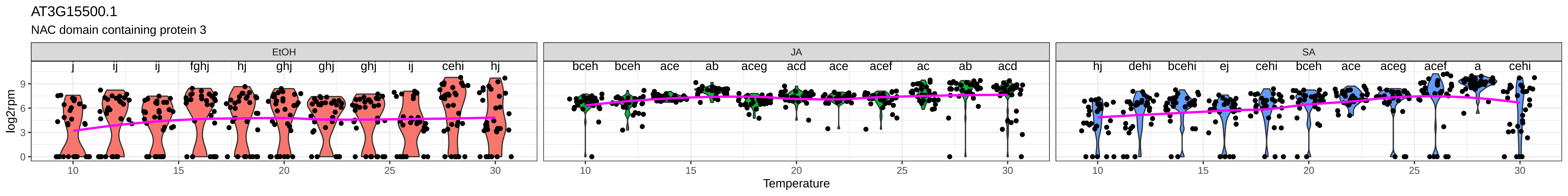
AT3G15500.1
NAC domain containing protein 3
EtOH
JA
SA
j ij ij fghj hj ghj ghj ghj ij cehi hj
bceh bceh ace ab aceg acd ace acef ac ab acd
hj dehi bcehi ej cehi bceh ace aceg acef a cehi
log2rpm
0
3
6
9
10
15
20
25
30
Temperature

AT5G52300.1

CAP160 protein

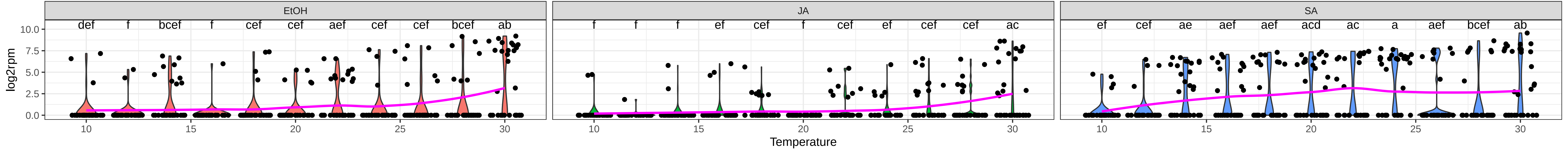

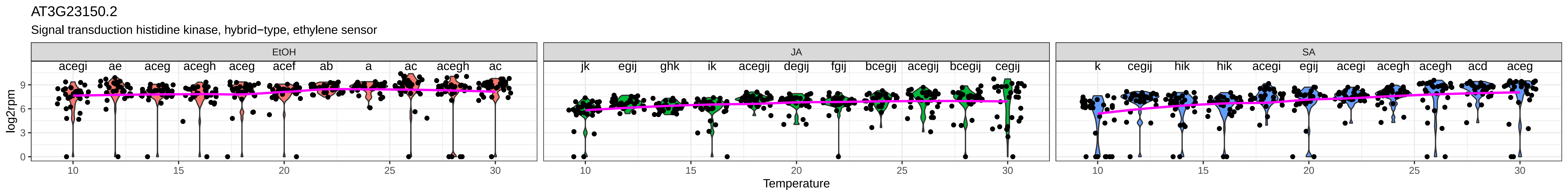

AT3G23150.2
Signal transduction histidine kinase, hybrid−type, ethylene sensor
EtOH
JA
SA
acegi ae aceg acegh aceg acef ab a ac acegh ac
jk egij ghk ik acegij degij fgij bcegij acegij bcegij cegij
k cegij hik hik acegi egij acegi acegh acegh acd aceg
log2rpm
0
3
6
9
10
15
20
25
30
Temperature

AT4G28910.2

novel interactor of JAZ

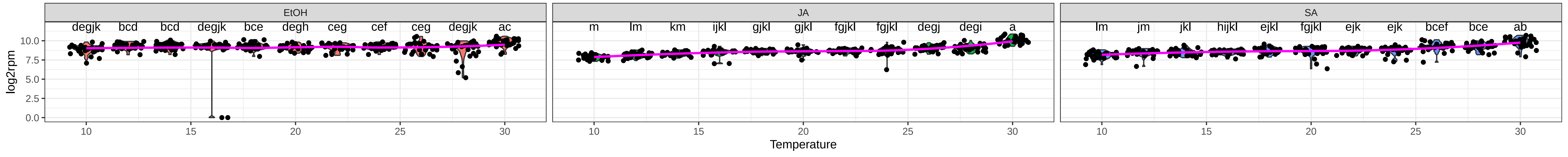

AT5G06950.5

bZIP transcription factor family protein

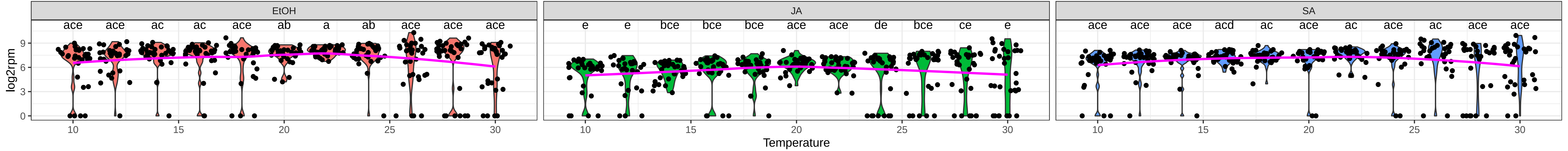

AT5G06960.1

OCS−element binding factor 5

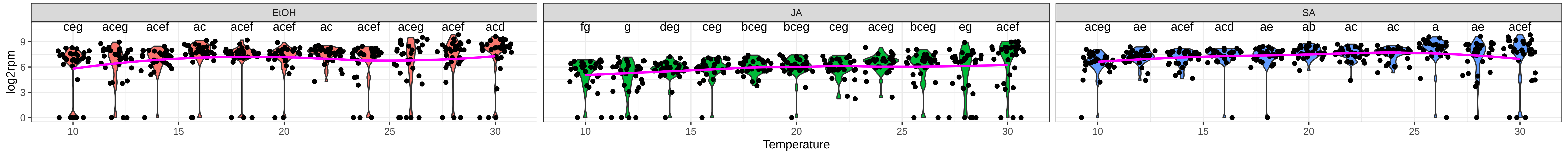

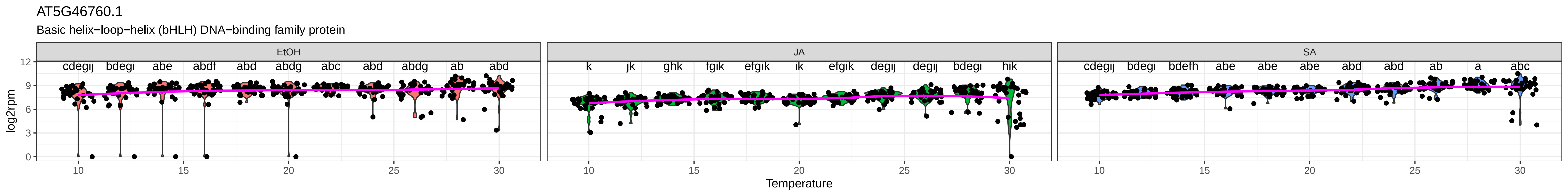

AT5G46760.1
Basic helix−loop−helix (bHLH) DNA−binding family protein
EtOH
JA
SA
log2rpm
Temperature
cdegij
bdegi
abe
abdf
abd
abdg
abc
abd
abdg
ab
abd
k
jk
ghk
fgik
efgik
ik
efgik
degij
degij
bdegi
hik
cdegij
bdegi
bdefh
abe
abe
abe
abd
abd
ab
a
abc

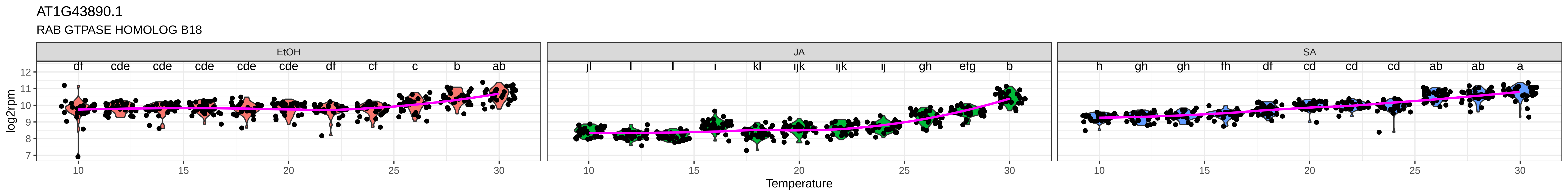

AT1G43890.1
RAB GTPASE HOMOLOG B18
EtOH
JA
SA
df cde cde cde cde cde df cf c b ab
jl l l i kl ijk ijk ij gh efg b
h gh gh fh df cd cd cd ab ab a
log2rpm
12
11
10
9
8
7
10
15
20
25
30
Temperature

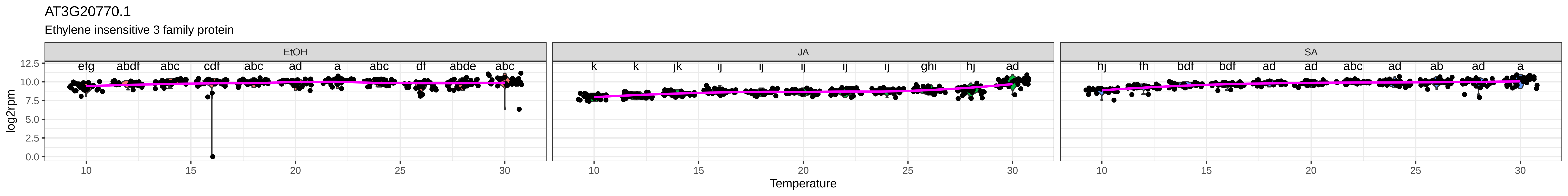
AT3G20770.1
Ethylene insensitive 3 family protein
EtOH
JA
SA
efg abdf abc cdf abc ad a abc df abde abc
k k jk ij ij ij ij ij ghi hj ad
hj fh bdf bdf ad ad abc ad ab ad a
log2rpm
12.5
10.0
7.5
5.0
2.5
0.0
10 15 20 25 30
Temperature

AT1G15750.3

Transducin family protein / WD−40 repeat family protein

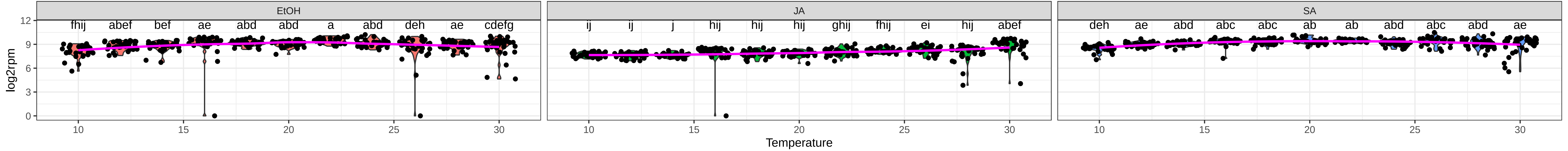

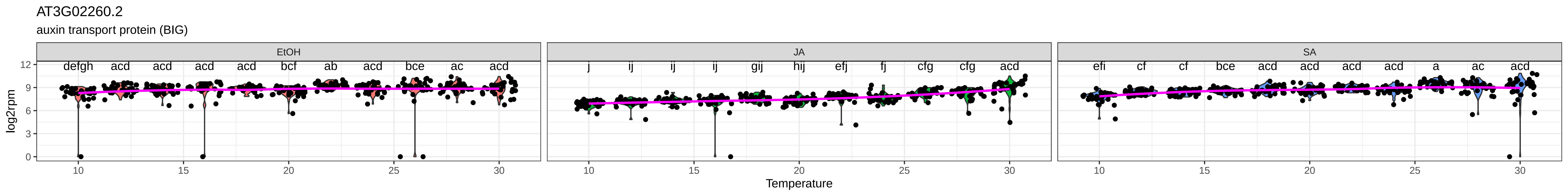

AT3G02260.2
auxin transport protein (BIG)
EtOH
JA
SA
defgh acd acd acd acd bcf ab acd bce ac acd
j ij ij ij gij hij efj fj cfg cfg acd
efi cf cf bce acd acd acd acd a ac acd
log2rpm
0
3
6
9
12
10
15
20
25
30
Temperature

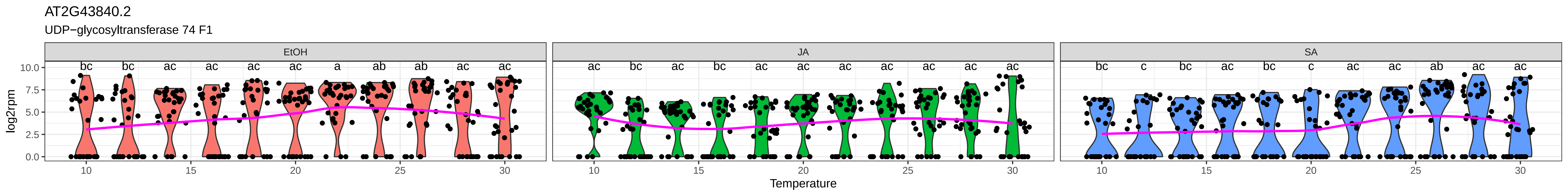

AT2G43840.2
UDP−glycosyltransferase 74 F1
EtOH
JA
SA
bc bc ac ac ac ac a ab ab ac ac
ac bc ac bc ac ac ac ac ac ac ac
bc c bc ac bc c ac ac ab ac ac
log2rpm
0.0
2.5
5.0
7.5
10.0
10
15
20
25
30
Temperature

AT1G73805.1

Calmodulin binding protein−like

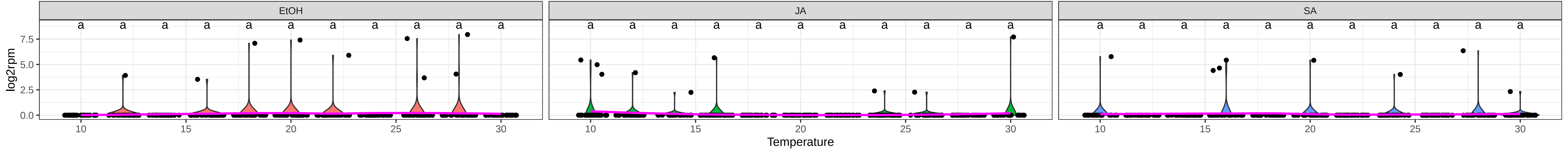

AT1G78390.1

nine−cis−epoxycarotenoid dioxygenase 9

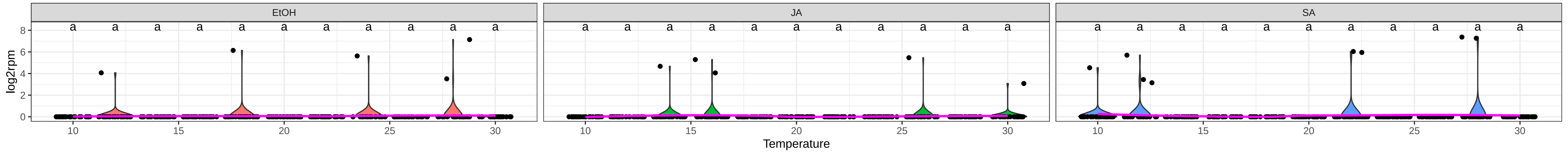

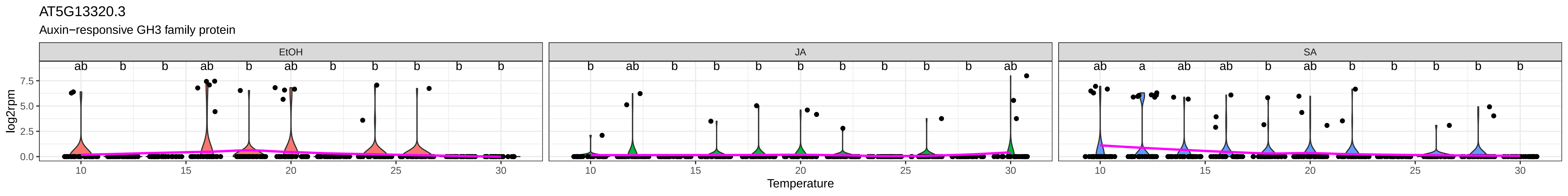

AT5G13320.3
Auxin−responsive GH3 family protein
EtOH
JA
SA
ab b b ab b ab b b b b b
b ab b b b b b b b b ab
ab a ab ab b ab b b b b b
log2rpm
0.0
2.5
5.0
7.5
10
15
20
25
30
Temperature

AT3G52430.1

alpha/beta−Hydrolases superfamily protein

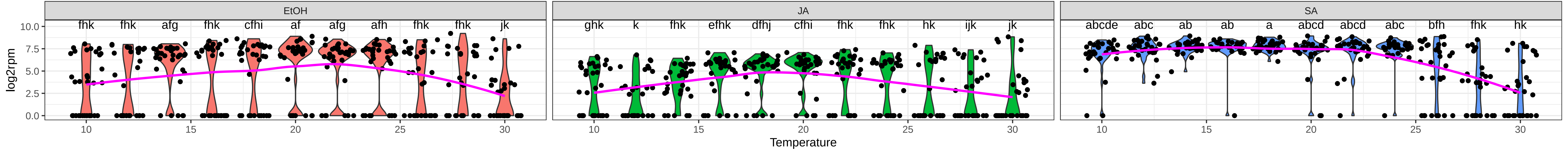

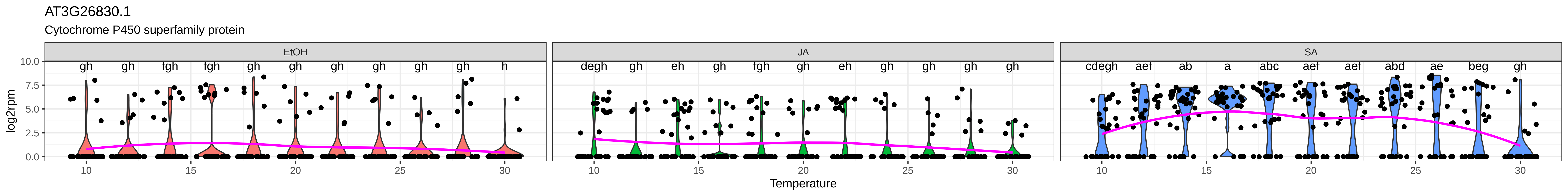

AT3G26830.1
Cytochrome P450 superfamily protein
EtOH
JA
SA
gh gh fgh fgh gh gh gh gh gh gh h
degh gh eh gh fgh gh eh gh gh gh gh
cdegh aef ab a abc aef aef abd ae beg gh
log2rpm
0.0
2.5
5.0
7.5
10.0
10
15
20
25
30
Temperature

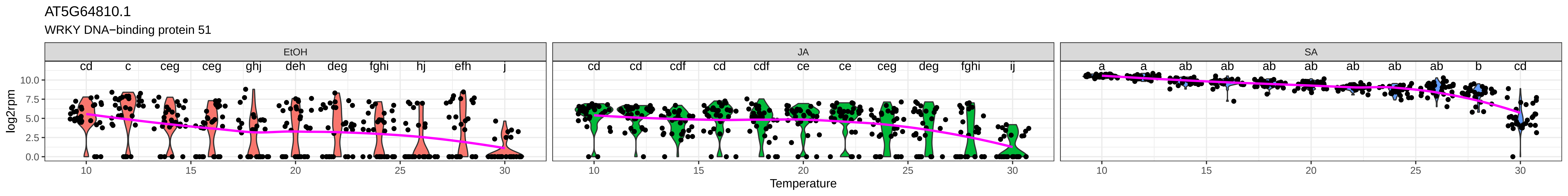

AT5G64810.1
WRKY DNA−binding protein 51
EtOH
JA
SA
cd c ceg ceg ghj deh deg fghi hj efh j
cd cd cdf cd cdf ce ce ceg deg fghi ij
a a ab ab ab ab ab ab ab b cd
log2rpm
0.0
2.5
5.0
7.5
10.0
10
15
20
25
30
Temperature

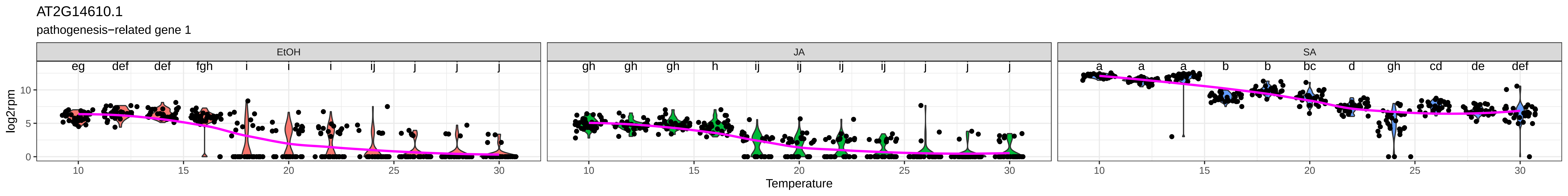
AT2G14610.1
pathogenesis−related gene 1
EtOH
JA
SA
eg
def
def
fgh
i
i
i
ij
j
j
j
gh
gh
gh
h
ij
ij
ij
ij
j
j
j
a
a
a
b
b
bc
d
gh
cd
de
def
log2rpm
0
5
10
10
15
20
25
30
Temperature

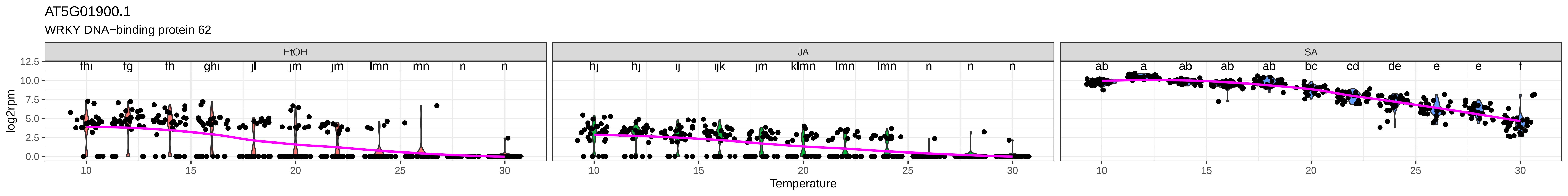
AT5G01900.1
WRKY DNA−binding protein 62
EtOH
JA
SA
fhi fg fh ghi jl jm jm lmn mn n n
hj hj ij ijk jm klmn lmn lmn n n n
ab a ab ab ab bc cd de e e f
log2rpm
12.5
10.0
7.5
5.0
2.5
0.0
10 15 20 25 30
Temperature

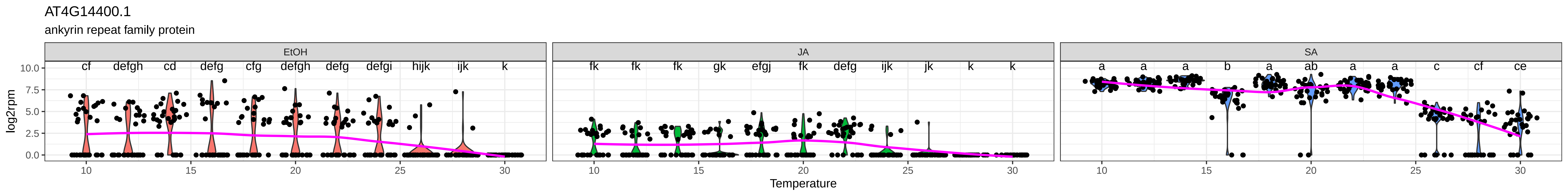
AT4G14400.1
ankyrin repeat family protein
EtOH
JA
SA
cf defgh cd defg cfg defgh defg defgi hijk ijk k
fk fk fk gk efgj fk defg ijk jk k k
a a a b a ab a a c cf ce
10.0
7.5
5.0
2.5
0.0
log2rpm
10 15 20 25 30
Temperature

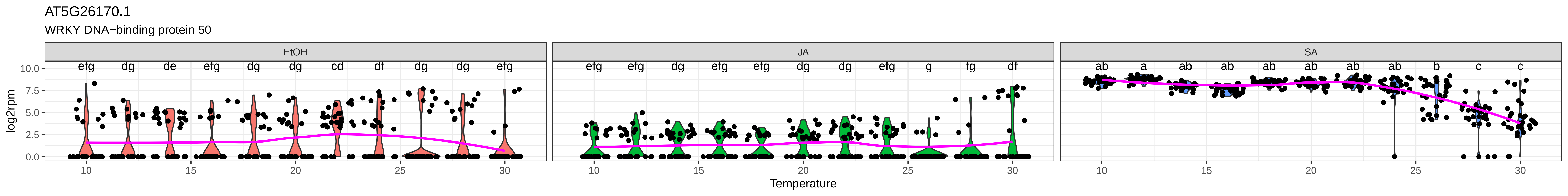

AT5G26170.1
WRKY DNA−binding protein 50
EtOH
JA
SA
efg dg de efg dg dg cd df dg dg efg
efg efg dg efg efg dg dg efg g fg df
ab a ab ab ab ab ab ab b c c
log2rpm
0.0
2.5
5.0
7.5
10.0
10 15 20 25 30
Temperature

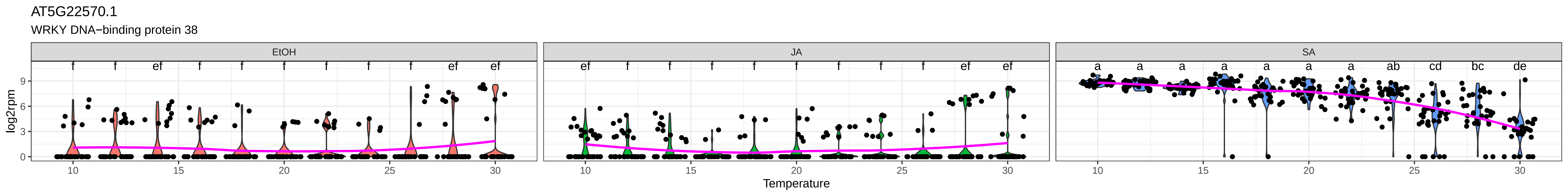
AT5G22570.1
WRKY DNA−binding protein 38
EtOH
JA
SA
f f ef f f f f f f ef ef
ef f f f f f f f f ef ef
a a a a a a a ab cd bc de
log2rpm
0
3
6
9
10
15
20
25
30
Temperature

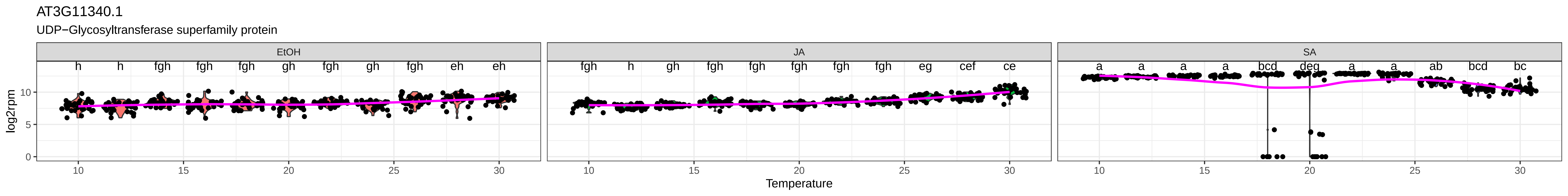
AT3G11340.1
UDP−Glycosyltransferase superfamily protein
EtOH
JA
SA
h
h
fgh
fgh
fgh
gh
fgh
gh
fgh
eh
eh
fgh
h
gh
fgh
fgh
fgh
fgh
fgh
eg
cef
ce
a
a
a
a
bcd
deg
a
a
ab
bcd
bc
log2rpm
10
5
0
10
15
20
25
30
Temperature

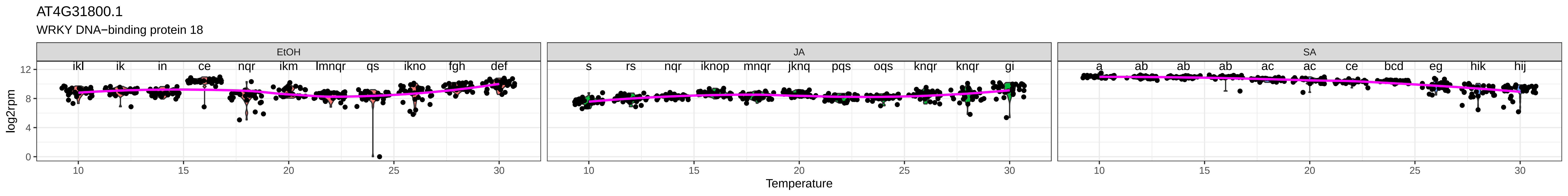

AT4G31800.1
WRKY DNA−binding protein 18
EtOH
JA
SA
ikl ik in ce nqr ikm lmnqr qs ikno fgh def
s rs nqr iknop mnqr jknq pqs oqs knqr knqr gi
a ab ab ab ac ac ce bcd eg hik hij
log2rpm
0
4
8
12
10
15
20
25
30
Temperature

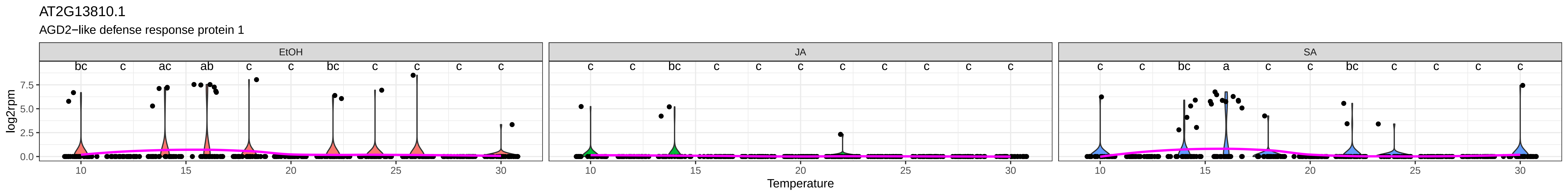

AT2G13810.1
AGD2−like defense response protein 1
EtOH
JA
SA
bc c ac ab c c bc c c c c
c c bc c c c c c c c c
c c bc a c c bc c c c c
log2rpm
0.0
2.5
5.0
7.5
10
15
20
25
30
Temperature

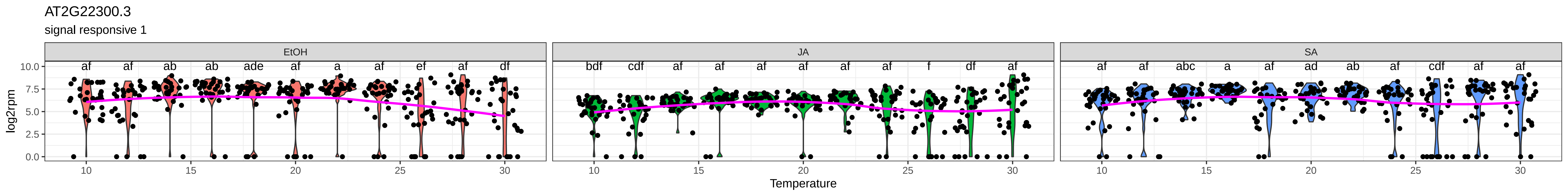

AT2G22300.3
signal responsive 1
EtOH
JA
SA
log2rpm
Temperature
af af ab ab ade af a af ef af df
bdf cdf af af af af af af f df af
af af abc a af ad ab af cdf af af

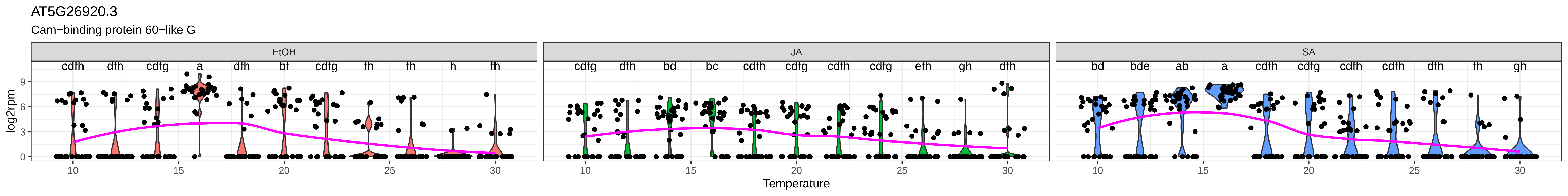

AT5G26920.3
Cam−binding protein 60−like G
EtOH
JA
SA
cdfh dfh cdfg a dfh bf cdfg fh fh h fh
cdfg dfh bd bc cdfh cdfg cdfh cdfg efh gh dfh
bd bde ab a cdfh cdfg cdfh cdfh dfh fh gh
log2rpm
0
3
6
9
10
15
20
25
30
Temperature

AT5G47230.1

ethylene responsive element binding factor 5

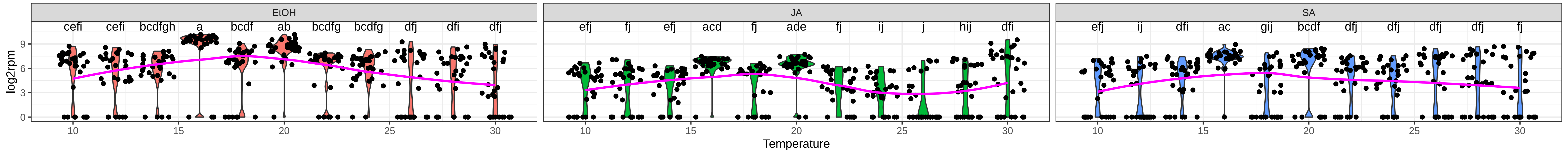

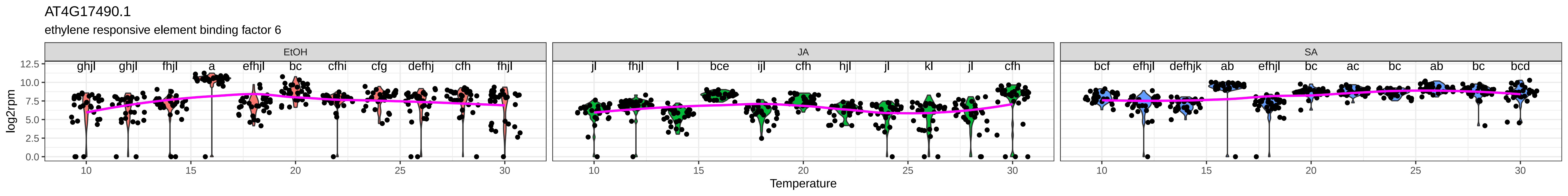
AT4G17490.1
ethylene responsive element binding factor 6
EtOH
JA
SA
ghjl ghjl fhjl a efhjl bc cfhi cfg defhj cfh fhjl
jl fhjl l bce ijl cfh hjl jl kl jl cfh
bcf efhjl defhjk ab efhjl bc ac bc ab bc bcd
log2rpm
12.5
10.0
7.5
5.0
2.5
0.0
10 15 20 25 30
Temperature

AT2G38470.1

WRKY DNA−binding protein 33

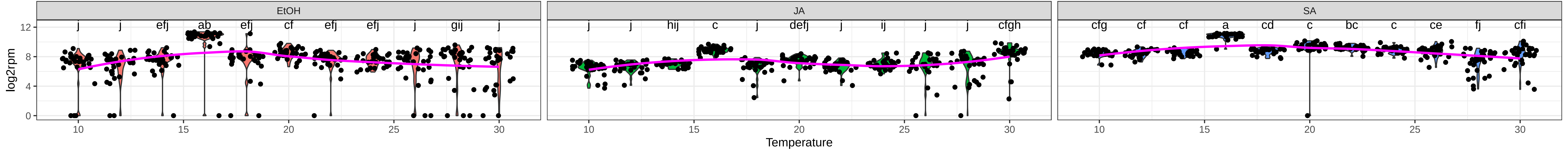

AT1G44575.2

Chlorophyll A−B binding family protein

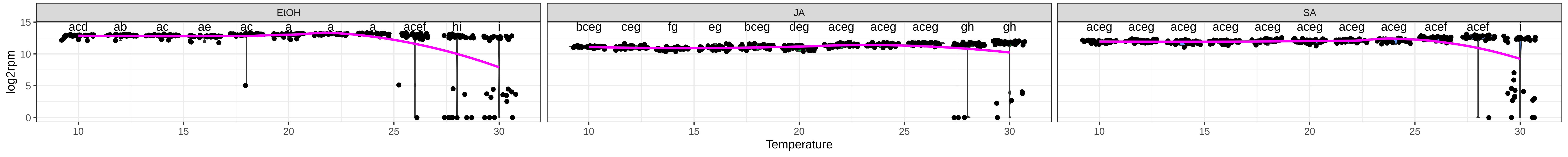

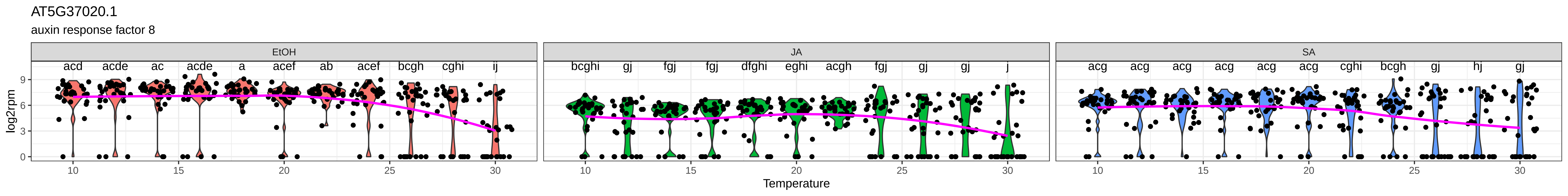
AT5G37020.1
auxin response factor 8
EtOH
JA
SA
acd acde ac acde a acef ab acef bcgh cghi ij
bcghi gj fgj fgj dfghi eghi acgh fgj gj gj j
acg acg acg acg acg acg cghi bcgh gj hj gj
log2rpm
0
3
6
9
10
15
20
25
30
Temperature

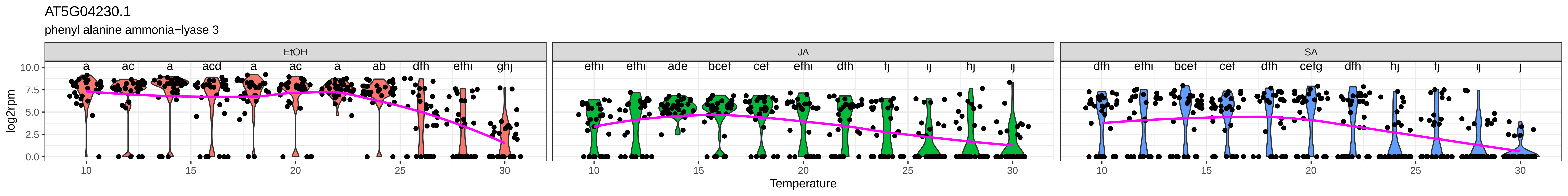

AT5G04230.1
phenyl alanine ammonia−lyase 3
EtOH
JA
SA
a
ac
a
acd
a
ac
a
ab
dfh
efhi
ghj
efhi
efhi
ade
bcef
cef
efhi
dfh
fj
ij
hj
ij
dfh
efhi
bcef
cef
dfh
cefg
dfh
hj
fj
ij
j
log2rpm
0.0
2.5
5.0
7.5
10.0
10
15
20
25
30
Temperature

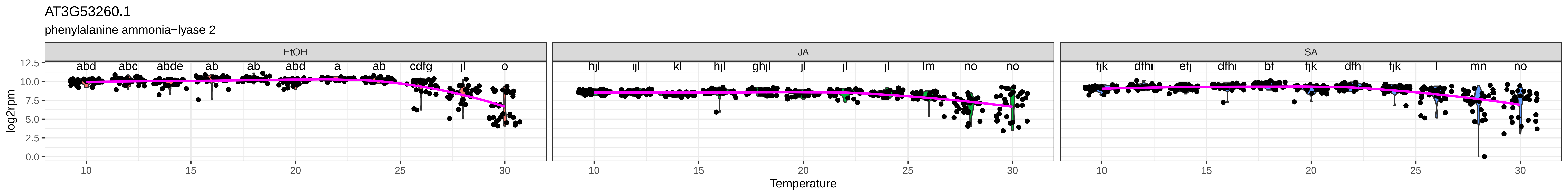
AT3G53260.1
phenylalanine ammonia−lyase 2
EtOH
JA
SA
abd abc abde ab ab abd a ab cdfg jl o
hjl ijl kl hjl ghjl jl jl jl lm no no
fjk dfhi efj dfhi bf fjk dfh fjk l mn no
12.5
10.0
7.5
5.0
2.5
0.0
log2rpm
10 15 20 25 30
Temperature

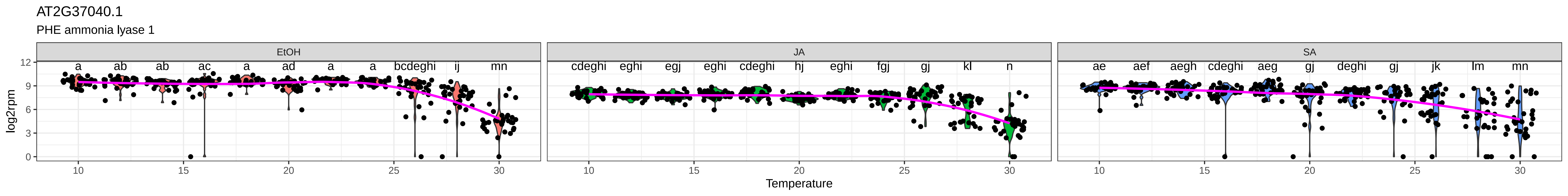
AT2G37040.1
PHE ammonia lyase 1
EtOH
JA
SA
a ab ab ac a ad a a bcdeghi ij mn
cdeghi eghi egj eghi cdeghi hj eghi fgj gj kl n
ae aef aegh cdeghi aeg gj deghi gj jk lm mn
log2rpm
0 3 6 9 12
10 15 20 25 30
Temperature

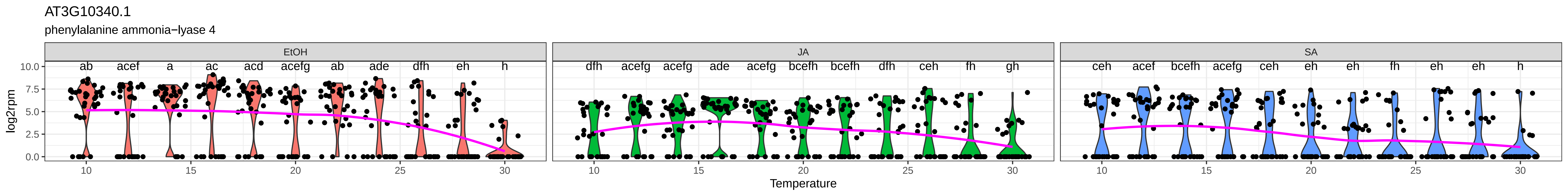
AT3G10340.1
phenylalanine ammonia−lyase 4
EtOH
JA
SA
log2rpm
Temperature
ab acef a ac acd acefg ab ade dfh eh h
dfh acefg acefg ade acefg bcefh bcefh dfh ceh fh gh
ceh acef bcefh acefg ceh eh eh fh eh eh h
0.0
2.5
5.0
7.5
10.0
10 15 20 25 30

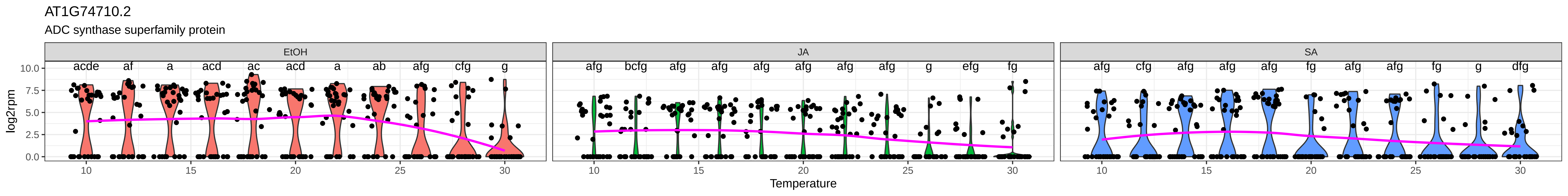

AT1G74710.2
ADC synthase superfamily protein
EtOH
JA
SA
acde af a acd ac acd a ab afg cfg g
afg bcfg afg afg afg afg afg afg g efg fg
afg cfg afg afg afg fg afg afg fg g dfg
log2rpm
10.0
7.5
5.0
2.5
0.0
10 15 20 25 30
Temperature

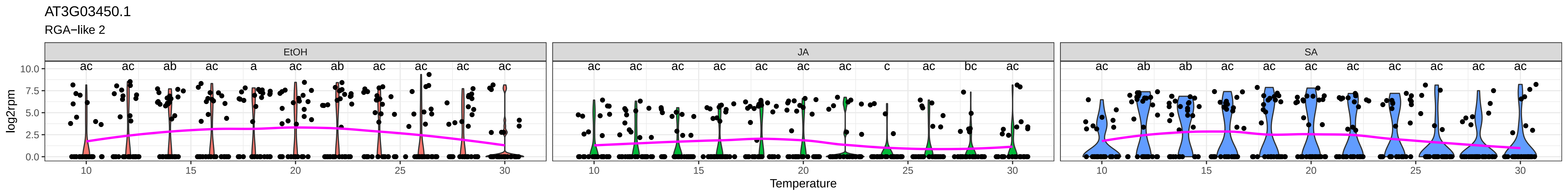
AT3G03450.1
RGA−like 2
EtOH
JA
SA
ac ac ab ac a ac ab ac ac ac ac
ac ac ac ac ac ac ac c ac bc ac
ac ab ab ac ac ac ac ac ac ac ac
log2rpm
0.0
2.5
5.0
7.5
10.0
10
15
20
25
30
Temperature

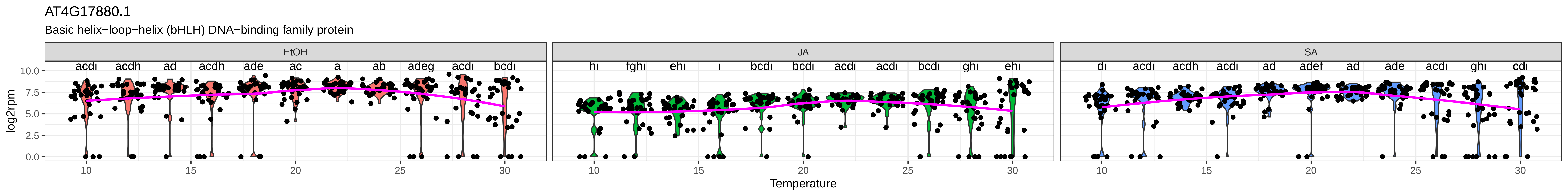

AT4G17880.1
Basic helix−loop−helix (bHLH) DNA−binding family protein
EtOH
JA
SA
acdi acdh ad acdh ade ac a ab adeg acdi bcdi
hi fghi ehi i bcdi bcdi acdi acdi bcdi ghi ehi
di acdi acdh acdi ad adef ad ade acdi ghi cdi
log2rpm
0.0
2.5
5.0
7.5
10.0
10 15 20 25 30
Temperature

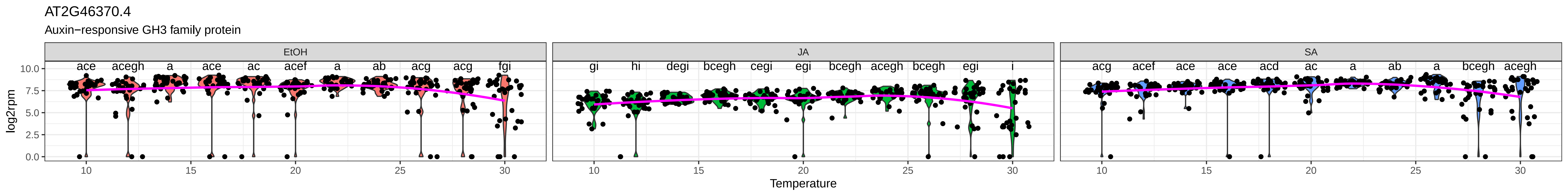

AT2G46370.4
Auxin−responsive GH3 family protein
EtOH
JA
SA
ace
acegh
a
ace
ac
acef
a
ab
acg
acg
fgi
gi
hi
degi
bcegh
cegi
egi
bcegh
acegh
bcegh
egi
i
acg
acef
ace
ace
acd
ac
a
ab
a
bcegh
acegh
log2rpm
10.0
7.5
5.0
2.5
0.0
10
15
20
25
30
Temperature

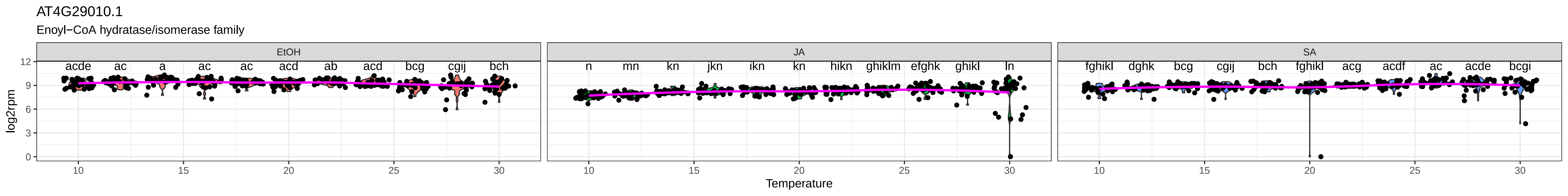
AT4G29010.1
Enoyl−CoA hydratase/isomerase family
EtOH
JA
SA
acde ac a ac ac acd ab acd bcg cgij bch
n mn kn jkn ikn kn hikn ghiklm efghk ghikl ln
fghikl dghk bcg cgij bch fghikl acg acdf ac acde bcgi
log2rpm
0
3
6
9
12
10 15 20 25 30
Temperature

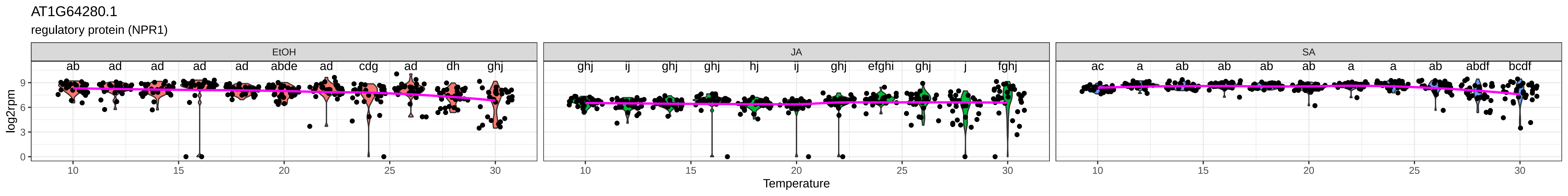

AT1G64280.1
regulatory protein (NPR1)
EtOH
JA
SA
ab ad ad ad ad abde ad cdg ad dh ghj
ghj ij ghj ghj hj ij ghj efghi ghj j fghj
ac a ab ab ab ab a a ab abdf bcdf
log2rpm
0
3
6
9
10
15
20
25
30
Temperature

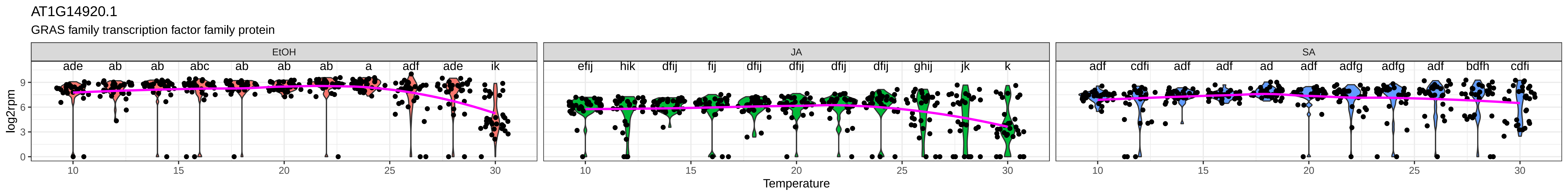

AT1G14920.1
GRAS family transcription factor family protein
EtOH
JA
SA
ade ab ab abc ab ab ab a adf ade ik
efij hik dfij fij dfij dfij dfij dfij ghij jk k
adf cdfi adf adf ad adf adfg adfg adf bdfh cdfi
log2rpm
0
3
6
9
10
15
20
25
30
Temperature

AT4G19100.1

Protein of unknown function (DUF3464)

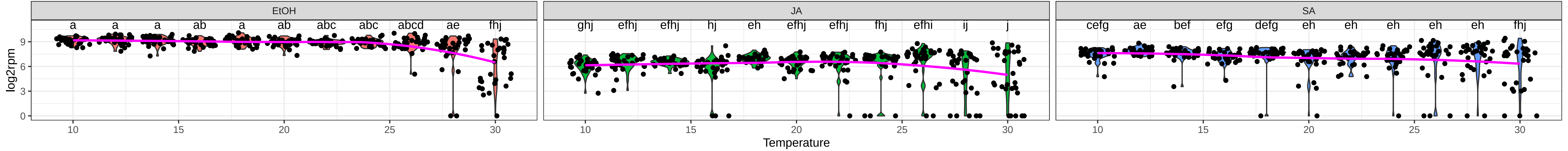

AT2G01570.1

GRAS family transcription factor family protein

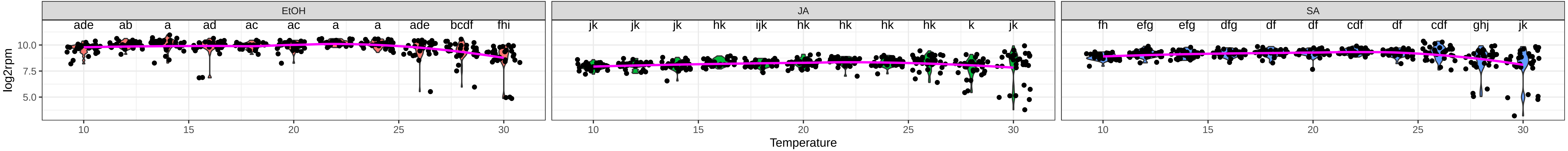

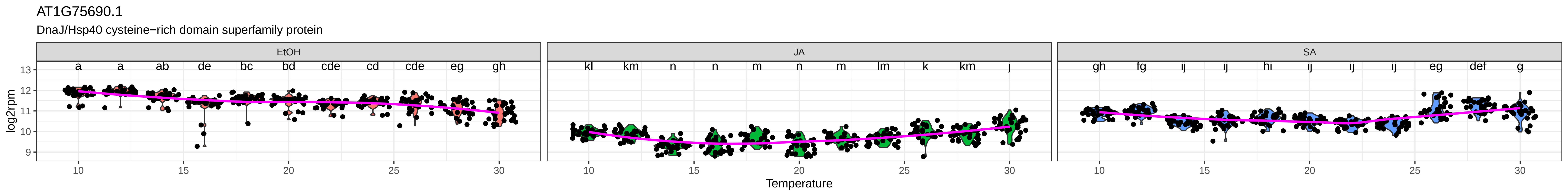

AT1G75690.1
DnaJ/Hsp40 cysteine−rich domain superfamily protein
EtOH
JA
SA
a a ab de bc bd cde cd cde eg gh
kl km n n m n m lm k km j
gh fg ij ij hi ij ij ij eg def g
log2rpm
13
12
11
10
9
10 15 20 25 30
Temperature

AT1G30330.3

auxin response factor 6

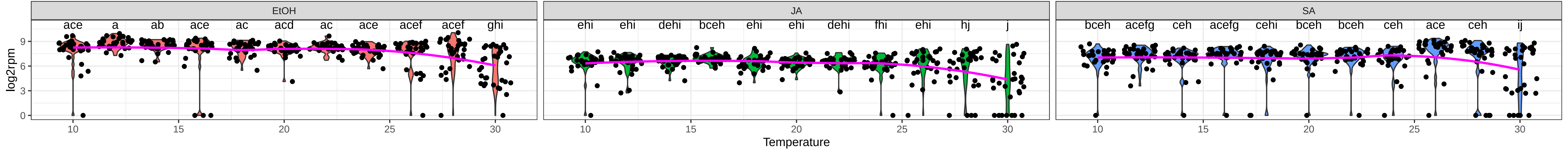

AT3G50660.2

Cytochrome P450 superfamily protein

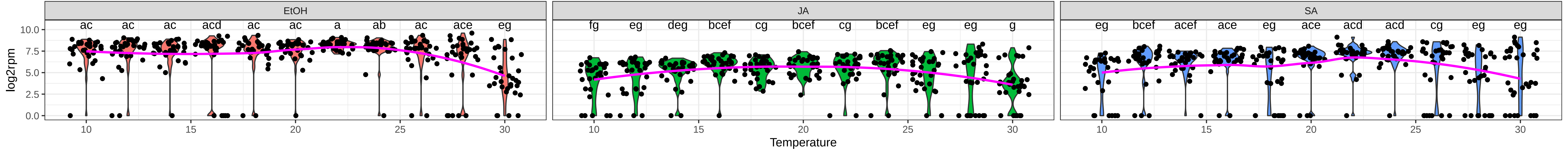

AT4G35760.2

NAD(P)H dehydrogenase (quinone)s

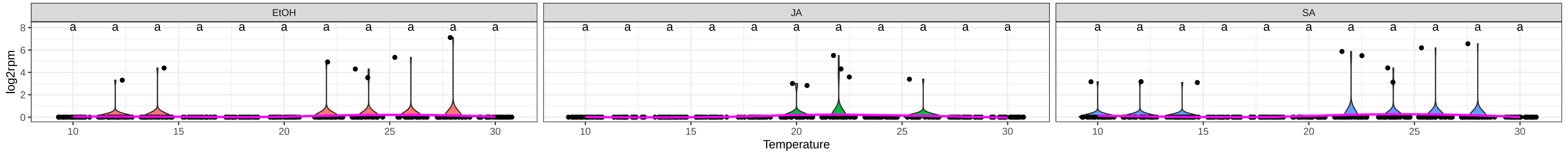

AT1G51190.1

Integrase−type DNA−binding superfamily protein

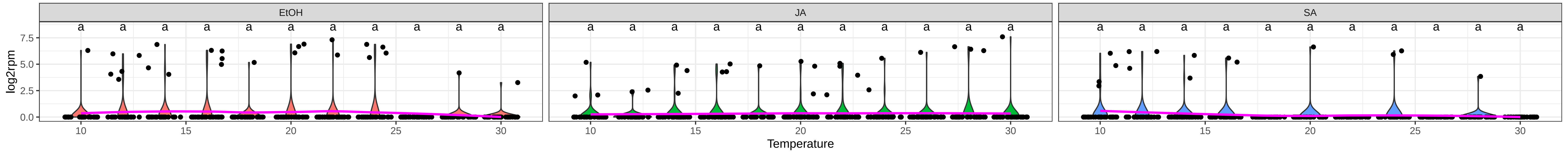

AT3G20840.1

Integrase−type DNA−binding superfamily protein

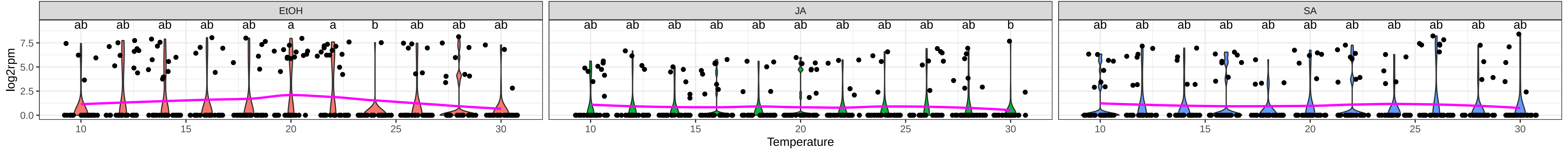

Supplement: Supplementary file 2 — Additional file 2: Data 2 Expressions of gene set 2 in EtOH-, jasmonic-acid-, and salicylic-acid-treated A. thaliana seedlings. Magenta lines represent trend curves of each target log2 RPM + 1 with the ambient temperatures. [file 13007_2022_930_MOESM2_ESM.pdf]
