# Supplementary material for: DeLTa-Seq: direct-lysate targeted RNA-Seq from crude tissue lysate
Source: Plant Methods. 2022 Aug 6;18:99. doi: 10.1186/s13007-022-00930-x (PMC9356424; doi:10.1186/s13007-022-00930-x)
Supplement: Supplementary file 4 — Additional file 4: Fig. S1. Performance of previously reported buffers for mammalian cultured cells in direct-lysate reverse transcription of A. thaliana, O. sativa, and D. rerio. Fig. S2. Comparison of RNA-Seq results of purified RNA and lysate of S. cerevisiae and D. rerio. Fig. S3. UMI conversion efficiencies of 96 technical replicates of non-targeted and targeted RNA-Seq. [file 13007_2022_930_MOESM4_ESM.docx]

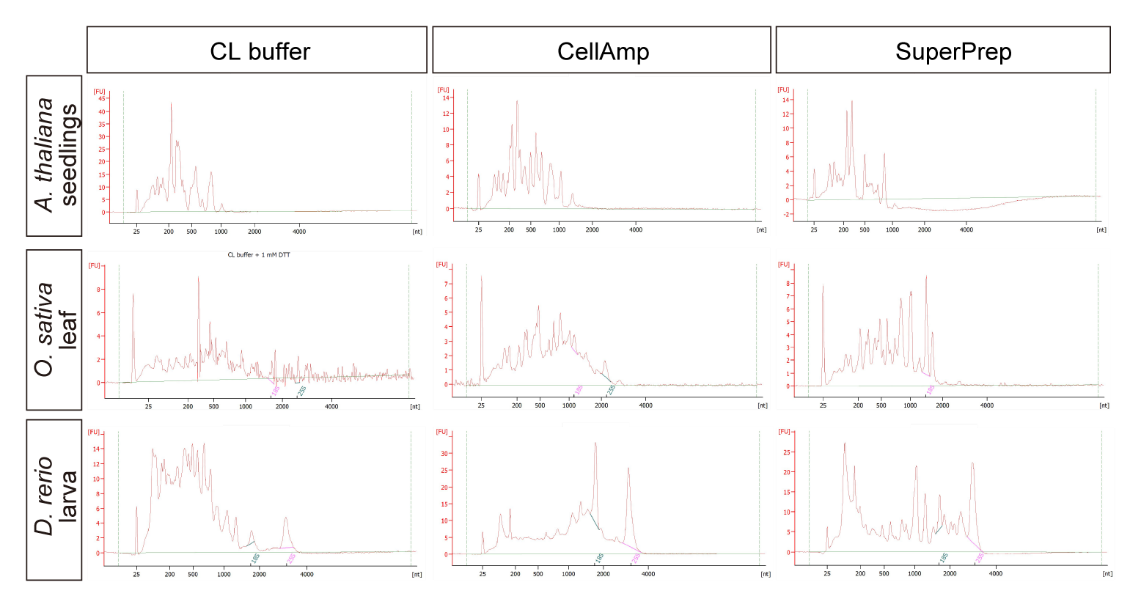


**Supplemental Figure 1 Performance of previously reported buffers for mammalian cultured cells in direct-lysate reverse transcription of *A. thaliana*, *O. sativa*, and *D. rerio*.**


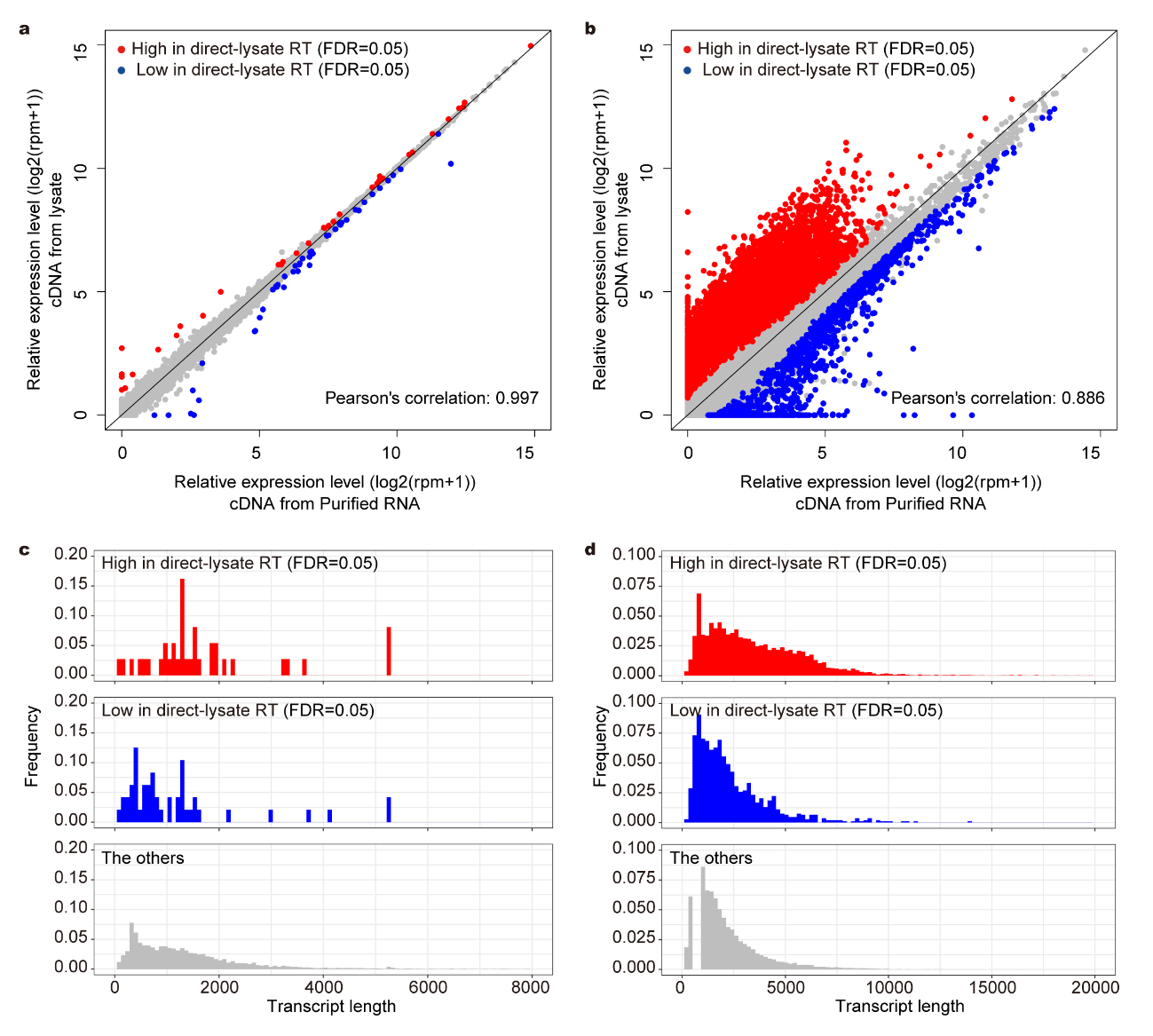


**Supplemental Figure 2 Comparison of RNA-Seq results of purified RNA and lysate of *S. cerevisiae* and *D. rerio*.**

The results of 3′ RNA-Seq of purified RNA and lysate of *S. cerevisiae* (a, c) and *D. rerio* (b, d) larva. (a, b) Scatter plot of log2 RPM+1 of each gene. Differentially quantified genes (DQGs) between purified RNA and lysate were detected (FDR = 0.05, n=6). (c, d) Transcript length distribution of DQGs with higher rpm values in the method using lysate and the other genes. The histograms show the results from 0 nt to 8000 nt in (c) and from 0 nt to 20000 nt in (d).


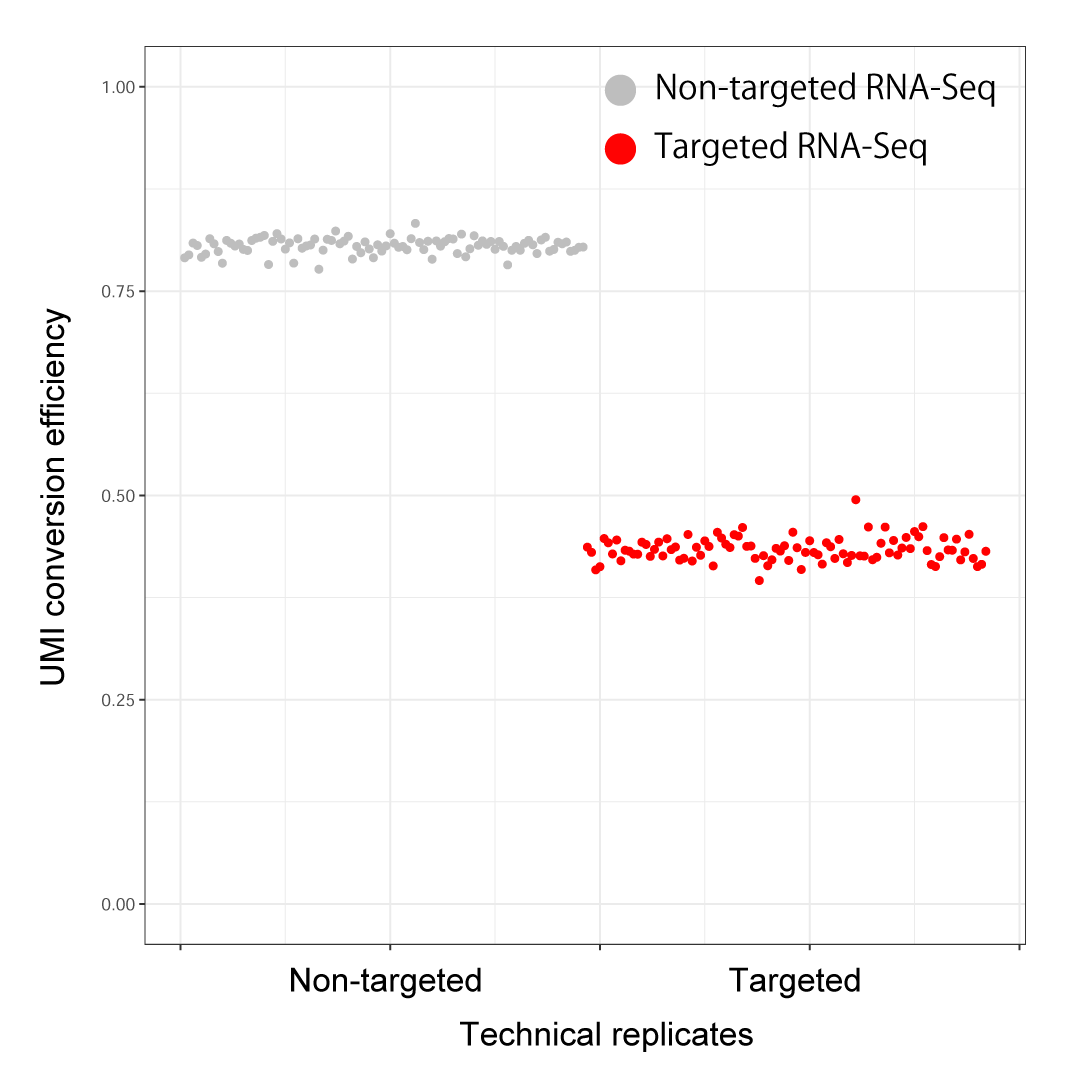


**Supplemental Figure 3 UMI conversion efficiencies of 96 technical replicates of non-targeted and targeted RNA-Seq.**

Plot of UMI conversion efficiencies of each technical replicate of non-targeted and targeted RNA-Seq.
